# Structural Origins of Voltage Hysteresis in the Na-Ion Cathode P2–Na_0.67_[Mg_0.28_Mn_0.72_]O_2_: A Combined Spectroscopic and Density Functional Theory Study

**DOI:** 10.1021/acs.chemmater.1c00248

**Published:** 2021-06-21

**Authors:** Euan N. Bassey, Philip J. Reeves, Michael A. Jones, Jeongjae Lee, Ieuan D. Seymour, Giannantonio Cibin, Clare P. Grey

**Affiliations:** † Department of Chemistry, University of Cambridge, Lensfield Road, Cambridge CB2 1EW, United Kingdom; ‡ School of Earth and Environmental Sciences, Seoul National University, Seoul 08826, Korea; § Department of Materials, Imperial College London, South Kensington Campus, London SW7 2AZ, United Kingdom; ∥ Diamond Light Source, Harwell Science and Innovation Campus, Didcot OX11 0DE, United Kingdom

## Abstract

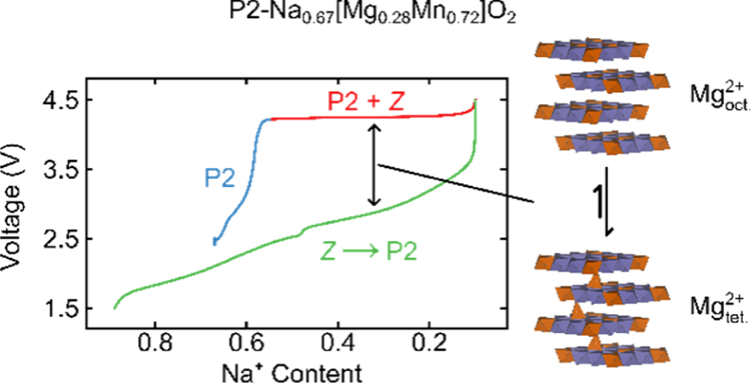

P2-layered sodium-ion battery (NIB) cathodes are a promising class of Na-ion electrode materials with high Na^+^ mobility and relatively high capacities. In this work, we report the structural changes that take place in P2–Na_0.67_[Mg_0.28_Mn_0.72_]O_2_. Using *ex situ* X-ray diffraction, Mn *K*-edge extended X-ray absorption fine structure, and ^23^Na NMR spectroscopy, we identify the bulk phase changes along the first electrochemical charge–discharge cycle—including the formation of a high-voltage “*Z* phase”, an intergrowth of the OP4 and O2 phases. Our *ab initio* transition state searches reveal that reversible Mg^2+^ migration in the *Z* phase is both kinetically and thermodynamically favorable at high voltages. We propose that Mg^2+^ migration is a significant contributor to the observed voltage hysteresis in Na_0.67_[Mg_0.28_Mn_0.72_]O_2_ and identify qualitative changes in the Na^+^ ion mobility.

## Introduction

Sodium-ion batteries (NIBs) are a more sustainable and significantly cheaper energy storage alternative to lithium-ion batteries (LIBs) and as such are poised to play a vital role in future grid-based energy storage.^1−4^ To date, the capacities of NIBs—limited by the cathode—are too low for many real-world applications, and the sources of NIB degradation have not received the attention that LIBs have.^1,5,6^ If we are to address the energy storage problem and improve the electrochemical performance of NIB cathodes, we need to understand the sources of these capacity losses in terms of the structural changes these cathodes undergo during charge and discharge.

**Figure 1:**
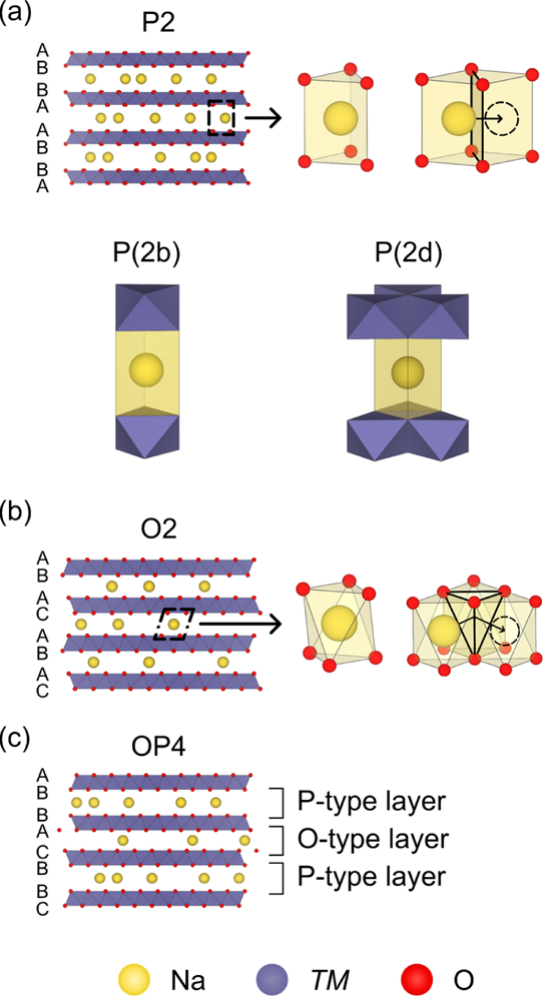
Structure of layered Na_*x*_TMO_2_ cathode materials. (a) Structure of P2 cathodes, with the local coordination environment of the P(2b) and P(2d) Na^+^ sites indicated, (b) structure adopted by O2 cathodes, and (c) structure adopted by OP4 cathodes. For the P2 and O2 materials, the local prismatic and octahedral Na^+^ coordination environments are shown, respectively, as well as the route taken by Na^+^ as it hops between adjacent sites. The TS for each Na^+^ hopping route is highlighted with a black outline.

Many studies have focused on layered transition metal (TM) oxides, Na_*x*_TMO_2_, as cathodes due to their high volumetric capacities and energy densities.^7^ The structures adopted by Na_*x*_TMO_2_ may be described using Delmas et al.’s notation,^8^ where a letter denotes the local coordination environment of Na^+^ ions (typically P or O for prismatic or octahedral coordination, respectively) and a number describes the number of TMO_2_ or Na^+^ layers per unit cell. Among the different structure types, the P2 structure is perhaps the most promising for cathodes:^7^ the prismatic Na^+^ sites—known as P(2d), where sites share edges with the TMO_6_ octahedra, and P(2b), for sites which share faces with TMO_6_ octahedra (Figure 1a)—are separated by large, open faces, leading to low energy barriers for Na^+^ hopping and enabling higher-rate cycling as compared to that of O-type compounds.^5^ Such O-type phases generally have a larger number of Na^+^ sites but a lower Na^+^ mobility due to the high-energy tetrahedral transition state (TS) associated with Na^+^ hopping [Figure 1b].^5,9^

On desodiating (charging) Na_*x*_TMO_2_, the TMO_2_ layers undergo layer gliding to minimize electrostatic repulsion between the eclipsed O^2–^ ions in adjacent layers and to stabilize Na^+^ vacancies.^10−12^ For example, P2 materials can undergo layer shearing to produce an O2 structure by shearing every layer (Figure 1b), or an OP4 structure can be formed by shearing alternate layers (giving alternating P-type and O-type Na^+^ layers) (Figure 1c).

To ensure long-term cyclability, Na_*x*_TMO_2_ should ideally either undergo fully reversible phase transformations or should not undergo phase transformations at all during cycling. Several studies have therefore focused on increasing the composition range over which the pristine structure is retained,^13−17^ particularly in the promising P2-type Na_*x*_MnO_2_ materials.^18−21^ An effective method is to dope the TMO_2_ layer with the electrochemically inactive species such as Li, Zn, and Mg,^22−25^ which replace some of the Jahn–Teller-active Mn^3+^ centers known to cause several of the phase transformations in P2-type Na_*x*_MnO_2_.^26^ As a result, smoother electrochemical profiles, longer lifetimes, and higher rate capabilities are observed.^25^ Inspired by the successes of these doped materials, we examined Na_0.67_[Mg_0.28_Mn_0.72_]O_2_ (henceforth termed NMMO), a high-capacity P2 NIB cathode material.

Previous studies have shown that the phase behavior of the Na_0.67_[Mg_*y*_Mn_1–*y*_]O_2_ family during cycling depends on the concentration of Mg^2+^. At low Mg^2+^ concentrations (*y* = 0.05 and 0.10), Mg^2+^ ions are distributed randomly over the TMO_2_ layer. The same transformation from P2 to OP4, as reported for Na_0.67_MnO_2_,^24^ was observed, but Mg^2+^ doping improved the reversibility of this transformation, resulting in increased cyclability, greater capacity retention, and a smoother voltage profile.^24,25^ At higher Mg^2+^ concentrations (*i.e.*, *y* = 0.28 for NMMO), Mg^2+^ ions undergo partial honeycomb ordering over the pristine material’s TM sublattice (as described in more detail below), and a P2-to-O2 phase transformation during charge was reported. In addition, a large reversible capacity was seen at high voltages (beyond 4.2 V vs Na^0/+^), which was attributed to oxygen redox.^27,28^ As with other O-redox-active cathode materials, NMMO is also plagued with a large voltage hysteresis, whose origin has yet to be explained.^28^

In this work, we show using X-ray diffraction (XRD) and extended X-ray absorption fine structure (EXAFS) that at high states of charge, NMMO undergoes a phase transformation from P2 to a mixture of OP4 and O2. We suggest that the mixture of OP4 and O2 phases may be described as a “*Z*-phase”:^29,30^ an intergrowth of OP4 and O2 phases, with little long-range order over neighboring Na^+^ and TMO_2_ layers. Using first principles calculations, we identify an energetically favorable process involving Mg^2+^ migration to the tetrahedral sites in the O-type layers in the OP4 and O2 phases. We calculate the Mg^2+^ diffusion pathway and activation energy barriers through single-ended TS searches and identify this migration as a source of the observed voltage hysteresis. The evolution of the local structure of NMMO is revealed through *ex situ*^23^Na NMR spectroscopy, and we assign these spectra using *ab initio* calculations of the ^23^Na NMR shifts. Finally, a description of the evolution of the structure of NMMO during charge and discharge, which is consistent with the bulk and local structural investigations, is presented.

## Experimental Section

### Synthesis

The synthetic route used was broadly similar to the previously reported routes (see the Supporting Information for more details).^27,28^ NMMO was synthesized in 1–2 g batches *via* a high-temperature solid-state reaction (10 h at 1073 K under flowing O_2_ gas, followed by a second step where the sample is heated to 973 K under flowing Ar and immediately quenched) between Na_2_CO_3_, MgO, and Mn_2_O_3_.^28^ All batches were synthesized using the same initial mixture of precursors. The final product is moisture-sensitive and must be stored in a dry atmosphere.

Electrodes of NMMO were made inside an Ar-filled glovebox by casting a slurry of NMMO, carbon super P (TIMCAL), and a PVDF binder (Kynar homopolymer) dispersed in *N*-methyl-2-pyrrolidone (Sigma-Aldrich, anhydrous, 99.5%) using an active material/carbon/binder mass ratio of 8:1:1 and an active material loading of 1.5–9.5 mg cm^–2^. Circular electrodes (13 mm diameter) were punched out and subsequently dried at 120 °C for 12 h under dynamic vacuum.

The electrolyte used throughout this work was 1.0 M NaPF_6_ (Acros Organics, 98.5+%; dried at 120 °C for 12 h under dynamic vacuum) dissolved in propylene carbonate (Solvionic, anhydrous). All cells in this work were half cells, with Na metal discs as anodes; these discs were punched from the Na metal (Sigma-Aldrich, 99.0%). All procedures described below (sample preparation, coin cell assembly and disassembly, and packing of NMR, XRD, and EXAFS samples) were performed in an Ar-filled glovebox with water and oxygen levels below 1 ppm.

### Electrochemistry

All electrochemical measurements were conducted using NMMO/Na half cells in 2032 stainless-steel coin cells. One cathode (13 mm diameter), one glass fiber separator (GF/B, Whatman; 16 mm diameter) soaked with 150 μL of the electrolyte, and one Na metal disc (13 mm diameter) were stacked and assembled into the cell.

A galvanostat/potentiostat (BioLogic) with EC laboratory software was used to perform electrochemical experiments for electrochemical assessment, *ex situ* NMR spectroscopy, and *ex situ* XRD. For *ex situ* Mn *K*-edge EXAFS samples, electrochemical experiments were carried out using an Arbin galvanostat/potentiostat. All half cells were cycled at a rate of 10 mA g^–1^, corresponding to approximately *C*/19, for a theoretical *C* rate relative to that of the pristine material, where this C rate has been calculated by assuming that *x* is 0.67 in the as-synthesized material Na_*x*_[Mg_0.28_Mn_0.72_]O_2_ and that *x* can vary between 0 and 1 on cycling. The actual (experimentally determined) values of *x* at different states of charge are then calculated from the time elapsed and current applied, assuming that no parasitic reactions occur and that all material is equally desodiated—*i.e.*, no Na^+^ ion concentration gradients build up.

### Powder X-ray Diffraction

In all cases, laboratory powder X-ray diffraction (PXRD) patterns were recorded using a PANalytical Empyrean diffractometer (Cu Kα radiation, λ = 1.541 Å). The samples were either sealed in a borosilicate glass capillary tube (for the pristine powder) or in air-tight sample holders (for *ex situ* cathodes) during the experiment. For the pristine material, 14 diffraction patterns over the range 2θ = 5–90° were collected and then summed, while patterns for *ex situ* samples were recorded over the range 2θ = 5–80°. In both cases, the step size was 0.02° and the scanning speed 0.02° s^–1^. All Rietveld^31,32^ refinements and analysis of XRD data reported in this article were carried out using the TOPAS Academic 6 structure refinement software package.^33,34^

### Scanning Electron Microscopy

Samples were loaded onto the scanning electron microscopy (SEM) stage of the transfer module (Kammrath and Weiss, type CT0) under an inert atmosphere without exposure to air. SEM images were acquired with a Tescan MIRA3 FEG-SEM instrument at an acceleration voltage of 2.0 kV.

### *Ex Situ* Sample Preparation

All *ex situ* samples were prepared by cycling a cathode to a given cutoff voltage and allowing the cell to rest for at least 1 h; see the Supporting Information for the open circuit voltages recorded after the rest period (Table S1). The cell was opened inside an Ar-filled glovebox and the cathode extracted, washed in dimethyl carbonate (approximately 1 cm;^3^ Sigma-Aldrich, 99%, anhydrous), and dried *in vacuo* for at least 20 min. The cathode was then either scraped off the Al foil current collector and cut up (for NMR measurements) or peeled off the Al foil current collector intact (for XRD and EXAFS).

### *Operando* X-ray Diffraction

*Operando* XRD measurements were carried out using a PANalytical Empyrean diffractometer (Cu Kα radiation, λ = 1.541 Å). The diffraction patterns were recorded at ambient temperature in the Bragg–Brentano geometry. All experiments were performed using an in-house *operando* cell. One cathode (with the Al foil peeled from the back), one glass microfiber separator (19 mm diameter, Whatman, Grade GF/B) soaked in the electrolyte (150 μL; despite the larger separator size than that of the coin cells, the electrolyte still wetted the separator adequately), and one Na metal disc (13 mm diameter) were stacked and assembled into the cell. The cell was left to rest for at least 1 h before beginning measurements. Diffraction patterns were recorded over the range 2θ = 5–50°, with a step size of 0.02° and a scanning speed of 0.02° s^–1^.

### Mn *K*-Edge Extended X-ray Absorption Fine Structure

*Ex situ* EXAFS was performed at beamline B18 at the Diamond Light Source. Mn *K*-edge data were recorded at ambient temperature in the transmission mode above and below the absorption edge of 6539 eV. Samples were loaded into an in-house (Diamond) transfer chamber with transparent polyimide (Kapton) film windows. Three spectral scans were recorded for each sample; no changes were observed for any sample between the first and last measurements. To fit the EXAFS data, Feff in the Artemis software package was used to calculate the contributions of different scattering paths to the observed data, with these paths fit using the in-built fitting algorithm in Artemis.^35,36^

### Nuclear Magnetic Resonance

*Ex situ* cycled cathodes were packed into 1.3 mm diameter ZrO_2_ magic angle spinning (MAS) rotors in an Ar-filled glovebox; no rotor spent longer than 10 min outside of the glovebox before being inserted into the magnet under a protective atmosphere of flushing nitrogen gas. ^23^Na NMR spectra were referenced to solid NaCl at 7.21 ppm. NMR spectra were acquired on a Bruker AVANCE III (11.7 T) using a Bruker 1.3 mm MAS probe, an MAS frequency of 60 kHz, and an effective π/2 pulse length of 0.59 μs (this corresponds to π/6, which is longer than the selective π/8 pulse to ensure that all quadrupolar ^23^Na centers are in the quadrupolar liquid limit;^37^ we therefore selected a compromise between the linear quadrupolar regime and maximizing the signal intensity). A rotor-synchronized Hahn-echo pulse sequence (90° – τ–180° – τ-acquire) was used. Spectra were scaled according to the mass of the sample and the number of residuals recorded. The recycle delay (25 ms; at least 5T_1_) was set such that the bulk, paramagnetically shifted signal was recorded quantitatively, while the diamagnetic signal due to electrolyte decomposition products was suppressed. Projection magic angle turning phase-adjusted sideband separation (pjMATPASS) experiments were also recorded to separate the isotropic resonances from the overlapping spinning sideband manifold.^38^

### First-Principles Calculations of NMR Hyperfine and Quadrupolar Shifts

To simplify calculations and account for the partial Na occupancies in NMMO, a model system, P2–Na_2/3_[Mg_1/3_Mn_2/3_]O_2_, was constructed to determine the approximate ^23^Na shifts expected for NMMO. Throughout these calculations, (2 × 1 × 2) supercells were used to account for both inter- and intralayer magnetic exchange interactions between Mn centers.

The ^23^Na shifts of each Na site were calculated using methods described previously.^39−42^ An initial geometry optimization was performed using the VASP code,^43−45^ employing the projector-augmented wave method.^46,47^ These calculations used the spin-polarized Perdew–Burke–Ernzerhof exchange–correlation functionals, applying the Hubbard *U* model^48,49^ within the rotationally invariant formalism proposed by Liechstenstein et al.,^50^ to correct for known deficiencies of pure functionals for highly localized 3*d* states.^51^ The plane-wave energy cutoff was set to 520 eV, and an effective Hubbard *U* parameter for Mn, *U*_eff_ = *U* – *J* = 3.9 eV, where *U* and *J* are the effective on-site Coulomb and exchange parameters (*J* = 1 eV), respectively, was chosen, in line with the previous work on the parent material, Na_*x*_MnO_2_.^93^ SCF cycles were converged with an energy tolerance of 10^–5^ eV. The Brillouin zone was sampled with a Monkhorst–Pack^52^*k*-point mesh with a mesh density of <0.5 Å^–1^.

Periodic spin-polarized density functional theory (DFT) calculations of the hyperfine and quadrupole-induced shifts were performed in CRYSTAL.^53^ Hyperfine parameters were calculated with B3LYP^54,55^ and a modified B3LYP hybrid functional containing 20 and 35% Hartree–Fock exchange, referred to as Hyb20 and Hyb35, respectively. These weights were chosen based on the success of these functionals in calculating the properties of TM compounds and have been previously reported to provide upper and lower bounds on experimental shifts.^39−41^

The calculations employed two basis sets: a smaller basis set for geometry optimizations (denoted BS-I) and a more extended set for the single-point hyperfine calculations (BS-II). The BS-I sets were taken—without modification—from solid-state studies by Catti et al.,^56−59^ while the BS-II sets comprised bases from the Ahlrichs set for metal ions^60^ and the IGLO-III basis set for O.^61^ Additional computational details, including the number of Gaussian primitives and the contraction scheme used for each basis set, alongside details of convergence criteria used, are provided in the Supporting Information.^62^

### Mg^2+^ Migration Energy Barrier Calculation and TS Searching

The searches were carried out using a method similar to that in refs (58) and (59). The migration of Mg^2+^ ions from the octahedrally coordinated TM layers to tetrahedral sites in the (vacant) Na^+^ layers was investigated using a hybrid eigenvector-following approach in the OPTIM code,^64−66^ with energies and gradients taken from an interface with VASP. The convergence parameters used in the minimization of the Rayleigh–Ritz ratio and tangent space minimization were the same as those found in ref (63). To initiate the TS search, the geometry-optimized structures of each system (containing octahedrally coordinated Mg^2+^) were modified by moving Mg^2+^ into the same plane as that of the O^2–^ ligands between the octahedral and tetrahedral sites.

The initial geometry optimization of the reactant structure (*i.e.*, Mg^2+^ in the octahedral site in the TMO_2_ layer) was carried out in VASP.^43−45^ The migrating Mg^2+^ center was then displaced so that it was coplanar with the O centers coordinating the O-type Na^+^ layer into which Mg^2+^ migrates to produce a “guessed” TS. Then, OPTIM was used to move the atomic positions, based on energies and gradients calculated in VASP with DFT + *U* and using the parameters outlined above. To model the van der Waals interactions in each model compound, calculations were repeated with increasingly accurate corrections and functionals: initially, a DFT-D3 correction was applied,^67^ followed by the OPT86B functional^68−71^ and finally the SCAN *meta*-GGA functional.^72−74^ The same effective Hubbard *U* parameter as above was used, but the plane-wave energy cutoff was set to 1000 eV when using the *meta*-GGA functional to ensure convergence to within 1 meV per formula unit.

TS searches for O2 and OP4 model systems were performed under fixed volume conditions in supercells of Na_10_Mg_5_Mn_13_O_36_ and Na_8_Mg_4_Mn_14_O_36_ for the O2 model system and Na_18_Mn_27_Mg_9_O_72_ for the OP4 model system. The unit cell parameters were fixed to those of geometry-optimized cells. Gamma-centered *k*-point grids were used to sample the reciprocal space for both systems, with a *k*-point sampling of <0.5 Å^–1^ in the Brillouin zone. The energy from single-point calculations was converged to 10^–6^ eV, while the root-mean-square forces were converged to below 10^–5^ eV Å^–1^.

## Results

### XRD of the Pristine Material

**Figure 2:**
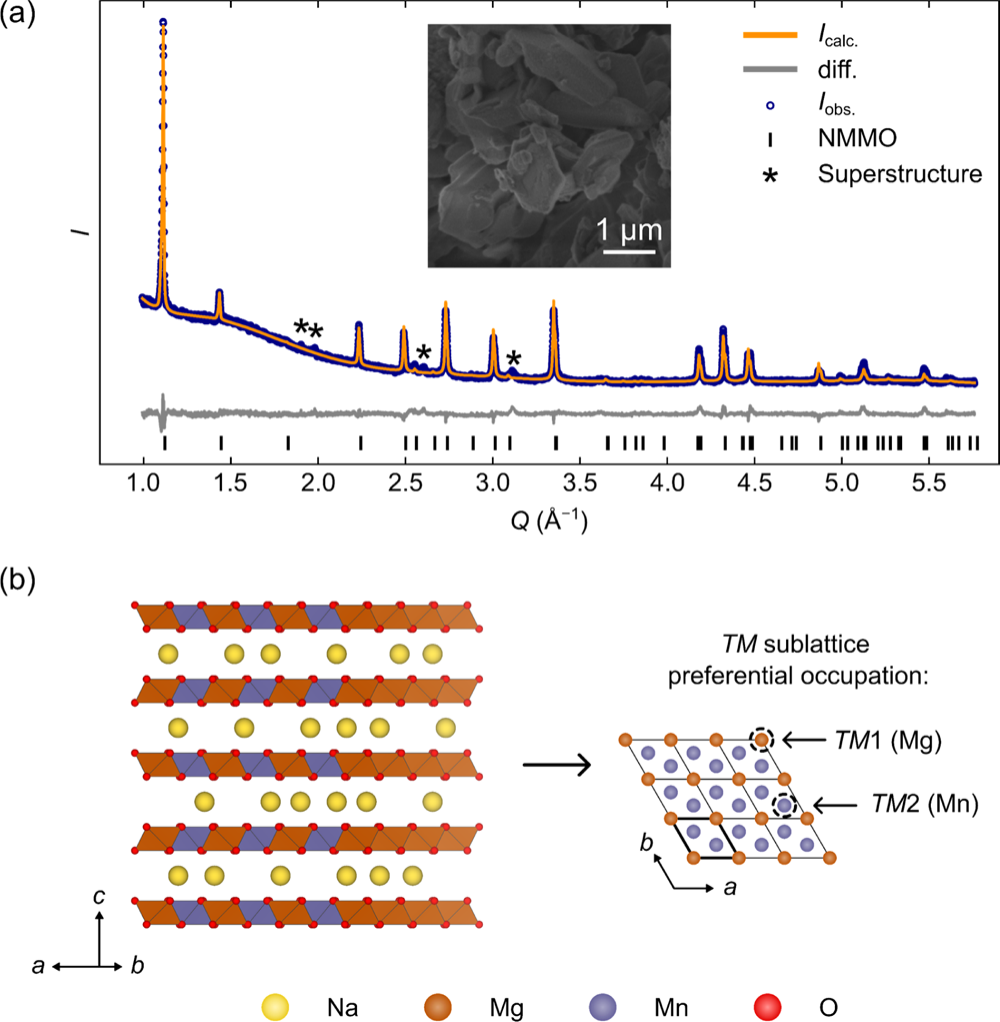
(a) Rietveld refinement of the PXRD pattern collected for pristine NMMO at room temperature (*R*_wp_ = 4.47%), with the inset showing an SEM image of pristine NMMO. Asterisks indicate reflections modeled by the larger (2 × 2 × 1) superstructure (see Figure S2). (b) Structure of NMMO: the left image highlights the stacking of layers in the structure, with the preferential occupation of the TM sublattice shown on the right (the unit cell is denoted with a bold outline).

The PXRD pattern for pristine NMMO was refined against a previously reported structural model,^31,32^ in which Mg and Mn are distributed across the TM sublattice and Na^+^ ions are distributed across the edge- and face-sharing Na^+^ prismatic sites (Figure 2a);^28^ no impurities were detected. Both the refined lattice parameters and site occupancies were consistent with previous reports (Table 1);^27,28^ the refined occupancies indicated an almost equal occupation of Mg and Mn at the TM(1) site (at the corners of the unit cell, Figure 2b) and preferential occupation of the TM(2) site by Mn (Figure 2b), suggesting partial honeycomb ordering over the TM sublattice. The refinement also revealed that the Na P(2b) sites had a higher occupancy than the Na P(2d) sites.

**Table 1:** Rietveld-Derived Lattice Parameters and Site Occupancies, Based on Laboratory PXRD Data Collected for Pristine NMMO Powder, *R*_w.p._ = 4.47%; Standard Errors Are Given in Parentheses^a^

space group			*P*6_3_/*mcm*		
*a* (Å)	5.0239(5)		α (°)	90	
*b* (Å)	5.0239(5)		β (°)	90	
*c* (Å)	11.2019(12)		γ (°)	120	
					
	site	*x*	*y*	*z*	Occ.
Mn(1)/Mg(1)	2*b*	0	0	0	0.51(10)/0.49(10)
Mn(2)/Mg(2)	4*d*	0.33333	0.66667	0	0.82(2)/0.18(10)
O	12*k*	0.354	0.354	0.08	1
Na(P(2d))	6*g*	0.301	0	0.25	0.35(10)
Na(P(2b))	4*c*	0.33333	0.66667	0.25	0.49(10)

a Note that the Na, Mg, and Mn site occupancies were constrained such that the stoichiometry of NMMO was fixed to be Na_0.67_[Mg_0.28_Mn_0.72_]O_2_; the occupancies of each TM site was also fixed to a total of 1; the atomic coordinates were fixed to those in ref (28) (reproduced with permission from Springer-Nature).

Overall, while the fit to the data is adequate, some peaks (*Q* = 1.90, 1.98, 2.60, and 3.11 Å^–1^; see Figure S1) could not be indexed with the current structure, and several reflection intensities were less well-modeled. We were, however, able to index all the additional peaks to a superstructure involving a (2 × 2 × 1) expansion of the unit cell. A Pawley refinement confirmed that this superstructure was able to model all of the reflections (Figure S2), suggesting that the poorer fit to some of the reflections using the smaller unit cell shown in Figure 2 can be ascribed to the additional ordering that creates this superstructure. Previous authors have identified superstructure reflections in this *Q* region to an ordering of Na^+^ ions and vacancies known as the “long zig-zag” (LZZ);^30,75,76^ indeed, the LZZ ordering has also been identified as a ground state ordering for P2 cathodes with a Na^+^ ion content of *x* = 0.67 (e.g., P2–Na_0.67_CoO_2_).^77,78^ On this basis, we attribute the superstructure to an LZZ ordering.

Additional refinements of the PXRD pattern to structural models incorporating stacking faults and/or a mixture of phases with honeycomb-ordered and random TM occupancy on the Mg/Mn sublattice gave poorer fits to the data, suggesting that the “random” TM distribution/honeycomb model (which showed preferential occupancies of Mg and Mn on the two TM sites) is a more accurate description of the pristine material structure. TEM and selected area electron diffraction images also confirmed the presence of partial honeycomb ordering over the TM sublattice (Figure S3). A more detailed analysis of both the honeycomb and (2 × 2 × 1) superstructures is beyond the scope of this work—in part because neutron diffraction measurements are required for a full analysis—but will be explored in a subsequent study.

### Electrochemistry

**Figure 3:**
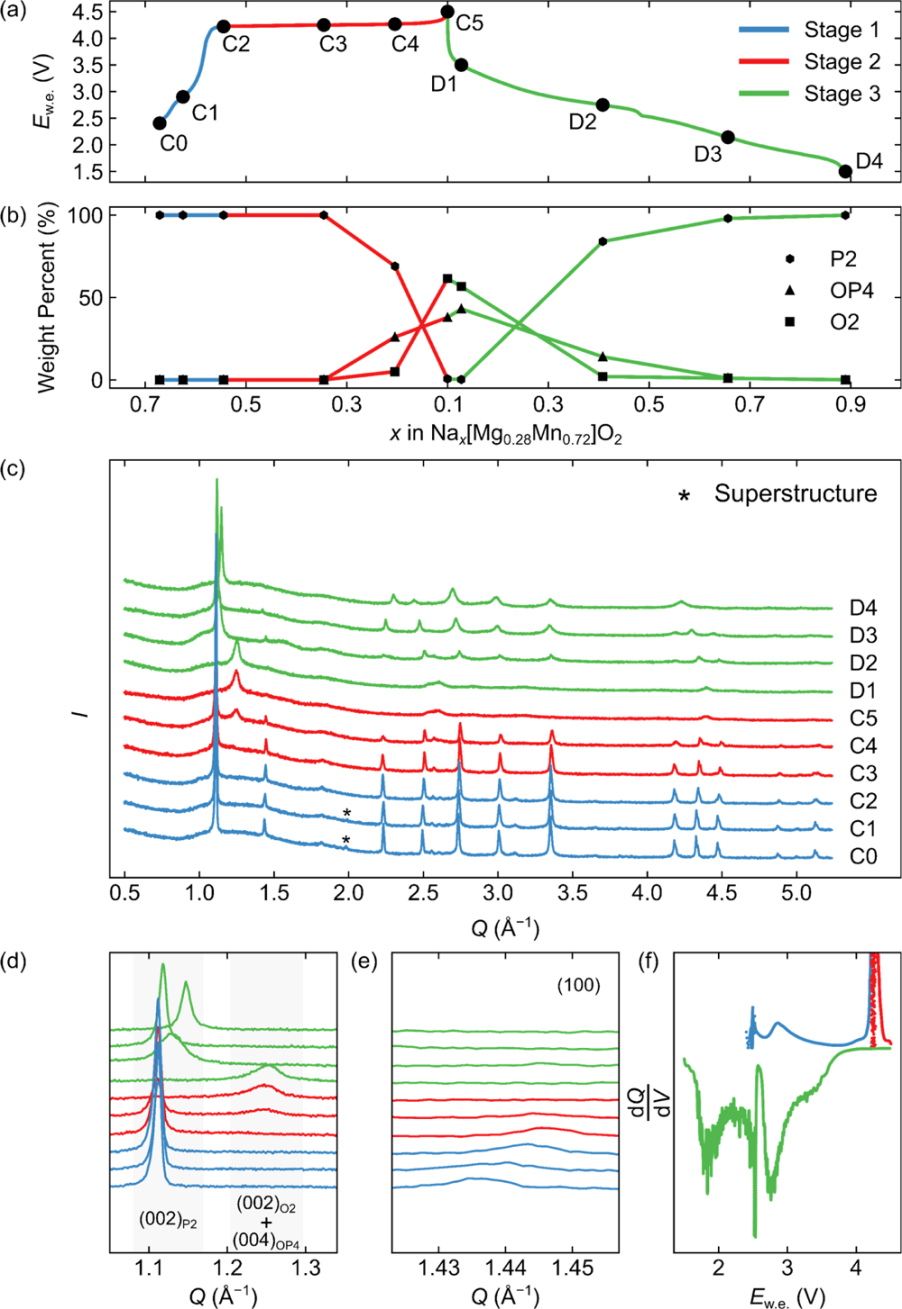
Electrochemical and *ex situ* XRD analysis of NMMO. The voltage profile for the first charge–discharge cycle of NMMO is shown in (a), with the changes in phase proportions during the first cycle plotted in (b) (note that the error bars are smaller than the marker size). (c) *Ex situ* laboratory XRD patterns recorded during the first cycle; the pristine P2 superstructure peaks, where observed, are indicated with asterisks, and expansions of two low-*Q* regions of the diffraction patterns are shown in (d,e). (d) corresponds to the (002) and (004) reflections for P2, O2, and OP4 phases of NMMO, while (e) shows the region of the *Q*-space corresponding to the (100) reflection for P2-NMMO. In (f), the differential voltage–capacity curve is shown for the first charge–discharge cycle.

The voltage profile for the first charge–discharge cycle of NMMO can be broken down into (at least) three distinct stages (Figure 3a). Stage 1 comprises the sloping region from the open circuit voltage to approximately 4.22 V, corresponding to approximately 20% of the total charge capacity. Stage 2 is the voltage plateau during charge; stage 3 is the discharge. Subsequent electrochemical cycles were observed to become more sloping—on both charge and discharge—with a smaller voltage hysteresis (Figure S4). The distinct voltage profiles for the first charge and discharge—observed with a large voltage hysteresis—indicates different sodiation and desodiation mechanisms in NMMO. This hysteresis is more clearly seen in the differential voltage–capacity curves (Figure 3f), where peaks in the differential voltage–capacity curve are observed at different potentials.

To understand the origin of this hysteresis and examine the bulk structural changes that take place during electrochemical cycling, we carried out *operando* and *ex situ* XRD (Figures 3c and S5–S15). Although quantitative Rietveld refinement of the *operando* diffraction data was not possible, useful qualitative information about the bulk structural evolution of NMMO can be obtained, where the phase transitions manifest themselves in dramatic changes to the observed pattern. For each *ex situ* XRD refinement, the background was fit with a 10-term Chebychev polynomial; the lattice parameters of all phases were allowed to vary freely, and the occupancies of Na, Mg, and Mn were allowed to vary while fixing the composition of each state of charge.

During stage 1, the diffraction patterns remain broadly similar, save small changes in peak positions and intensities, indicating Na^+^ extraction from a single phase, as expected from the sloping voltage profile. The superstructure peaks seen in pristine NMMO disappear by point C2, indicating a loss of the superstructure, likely caused by both the increasing Na^+^ ion disorder and oxidation of the Mn^3+^ ions to Mn^4+^.

During stage 2 (the charge plateau), a new low-*Q* peak (*ca.* 1.35 Å^–1^) appeared, suggesting the formation of a new phase. The appearance of the new low-angle peak was coincident with a broadening and decrease in intensity of the low-*Q* (002) peak from the P2 phase (Figure 3d). Previous studies assigned the new peak to an O2 phase,^27,28^ despite other members of the Na_*x*_[Mg_*y*_Mn_1–*y*_]O_2_ family forming an OP4 phase at high states of charge.^24^ Rietveld refinements^31,32^ of the *ex situ* diffraction patterns collected at high states of charge (points C4 and C5 in Figure 3) show that both OP4 and O2 phases are present along this charge plateau, the new low-*Q* peak corresponding to the (002) and (004) reflections of the O2 and OP4 phases, respectively. The data could not be modeled by using only P2 and O2 or only P2 and OP4 phases. The O2 and OP4 phases grow at the expense of the P2 phase (Figure 3b), suggesting that the P-type layers gradually transform to O-type on desodiation (presumably *via* layer slippage), as anticipated. At the end of the charge, both O2 and OP4 phases are present, but their low-*Q* reflections remain severely broadened and at similar *Q* values (4.87 and 4.91 Å^–1^ for (004)_OP4_ and (002)_O2_, respectively), indicating little long-range order in these phases.

While thermodynamically, only two phases should coexist during a two-phase reaction, the transformation from a P2 phase to an O phase is likely to be kinetically sluggish as it involves layer slippage. Many of the reflections from the OP4 and O2 phases were coincident, but some (such as the (006)_OP4_ and (105)_O2_) were distinct (Figures S10 and S11); we therefore propose that NMMO transforms from a P2 phase into what has been called a “*Z* phase” at high states of charge, as observed in other Mn-based layered NIB cathodes at high states of charge.^29,30^ This single (*Z*) phase can be conceptualized as an intergrowth of OP4 and O2 phases, with no long-range order between the O- and P-type layers and the concentrations of O layers within blocks of ordered OP4 regions increasing as Na is removed. In our Rietveld refinements, we refine the O2 and OP4 blocks (or phases) separately to account for the varying amounts of O- and P-type layers in this highly disordered phase.

As with other layered NIB cathodes known to exhibit a P2-to-*Z* phase transformation,^30,79^ the material at the end of the first charge is best described as an O2-like phase with residual P-type layers, in line with the previous assertion that an O2 phase forms at the end of the first charge.^27,28^ Rietveld refinements of the diffraction patterns at high states of charge against structural models with a random distribution of O- and P-type layers or refinements with variable numbers and probabilities of stacking faults were too computationally demanding and unsuccessful. Therefore, we will continue in this work to use the O2 and OP4 structures as models of the high-voltage phases but acknowledge that these are surrogates for the “true” *Z*-intergrowth phase.

Our refinements clearly showed the absence of Na^+^ in the O2-type layers. We then explored various models for the Mg^2+^ positions, including Mg^2+^ in either the tetrahedral or octahedral sites in the O-type Na^+^ layers of the O2 and OP4 phases and Mg^2+^ in the octahedral TMO_2_ sites (as in the pristine structure). All these refinements gave similar fits to the data (Figure S16), with a marginal improvement for Mg^2+^ migration out of the original TM sites. While we note that this migration is not conclusive based on the *ex situ* XRD results, the presence of Mg^2+^ in the tetrahedral sites of the O-type Na^+^ layers is consistent with the formation of a *Z* phase as it is hypothesized that *Z*-phase materials may be stabilized by TM migration^79^ and only form where P-type layers have been desodiated and undergo layer slippage to become O-type.^30^ This proposed Mg^2+^ migration is explored below *via* calculations.

The *operando* and *ex situ* diffraction patterns along stage 3 (discharge) initially remained essentially the same between points C5 and D1, suggesting no significant structural changes. However, the relative proportions of the O2 and OP4 phases changed slightly, with the OP4 phase growing at the expense of the O2 phase, perhaps indicating a “relaxation” of the structure, *via* layer slippage of some of the O-type layers back to P-type due to the small amount of Na^+^ (0.03 equivalents) inserted back into NMMO (Figure 3b).

After point D1, the low intensity (002) peak from the P2 phase grows in intensity and moves to a higher *Q*, toward the low-*Q* peaks of the OP4 and O2 phases (Figures 3c and S5). Eventually, the low-*Q* peaks of the O2, OP4, and P2 phases merge, with the P2 component growing at the expense of the O2 and OP4 components. At the end of discharge, the P2 phase dominates, with no OP4 and only a negligible amount of O2 present; we attribute any remaining O2 phase to small domains of the O2 phase trapped within a P2 matrix. All peaks in the diffraction pattern at the end of the first discharge are broader than those observed in the pristine material and in the early stages of charge, likely due to the decrease in crystallinity (loss of long-range ordering) and development of stacking faults induced by electrochemical cycling. Furthermore, the superstructure peaks seen in pristine NMMO do not return, indicating the loss of more long-range ordering beyond the P2 cell used in the structural refinements.

We also note that the (100) peak of the P2 phase—which indicates the presence of ordering across the Mg/Mn sublattice— significantly decreases in intensity (compared to the pristine material) on cycling, indicating that the loss of Mg/Mn ordering during charge is not regained on discharge; this is consistent with Mg^2+^ migration (Figures 3e and S4d).

### Extended X-ray Absorption Fine Structure

**Figure 4:**
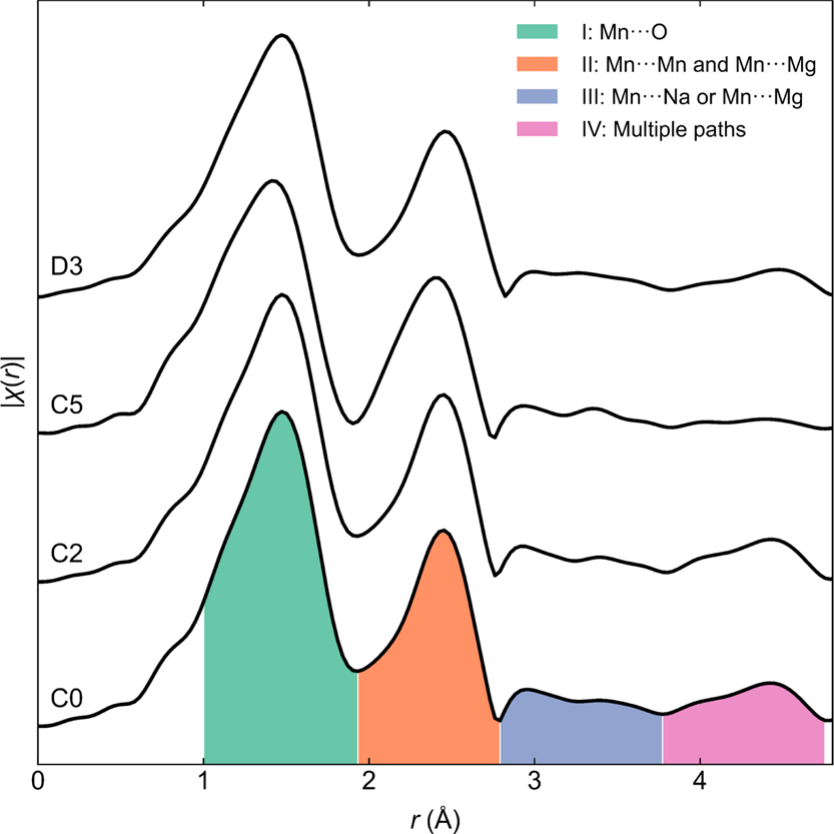
*Ex situ* Mn *K*-edge EXAFS data for NMMO at different states of charge, showing the four most prominent features in the data up to *r* = 4.8 Å.

To examine the changes in the structure during electrochemical cycling further, we carried out *ex situ* Mn *K*-edge EXAFS analysis on NMMO at points C0, C2, C5, and D3 along the voltage profile (Figure 4). The EXAFS plots show four significant features (I–IV, Figure 4), which were fitted to single- and double-scattering paths (Figures S17–S20). Data collected at the C0, C2, and D3 states were fitted against paths derived from a P2 structure only (with all Mg^2+^ in the octahedral sites in the TMO_2_ layers), while data from C5 were fitted against paths from both OP4 and O2 structures to reflect the dominant phase fractions at each of these states of charge. Again, we explored models for the OP4 and O2 phases, where Mg remained in its original location in the TMO_2_ layer or Mg^2+^ occupied either tetrahedral or octahedral sites in the O-type Na^+^ layers. In all cases, path calculations were carried out on (2 × 2 × 2) supercells of the dominant phase(s) at those states of charge; the lattice parameters for these supercells were taken from *ex situ* Rietveld refinements of the XRD data at the same state of charge. The fitted Debye–Waller factors and effective scattering distances for the dominant single-scattering paths from each element, X, (X = O, Mn, Mg, and Na), are presented in Table S1.

In all cases, feature I is dominated by scattering from O and feature II is dominated by scattering from Mn and Mg. Feature III is dominated by scattering from Na in all cases except at C5, where scattering from Mg^2+^ dominates. Feature IV is composed of a combination of several scattering paths.

Between C0 and C2, the effective Mn–O distance in NMMO decreases, as expected for Mn–O oxidation. In addition, the Mn–Mn distance increases between C0 and C2, likely due to the increase in the Coulombic repulsion between Mn centers (see the Discussion section). At the same time, the Mn–Mg distance decreases, likely due to the contraction of the Mn–O bonds induced by Mn oxidation.

On charging along the high-voltage plateau (*i.e.*, C2 to C5), the Mn–O distance continues to decrease in both the OP4 and O2 phases, again due to Mn–O oxidation. The intra-TMO_2_-layer Mn–Mn and Mn–Mg scattering distances also decrease in the OP4 and O2 phases, reflecting the shortened Mn–O bonds.

As can be seen in Figure S21, a noticeable improvement to the fit of feature III is seen on migration of Mg^2+^ into either the vacant tetrahedral or octahedral sites of the O-type Na^+^ layers. While not conclusive of Mg^2+^ migration, they provide additional support for the vacation of the TMO_2_ layers by Mg^2+^.

Finally, between the end of the first charge (C5) and the discharged (D3) states, the Mn–O distance returns to almost the same value as that of the pristine material, as expected for similar Na^+^ contents and Mn–O oxidation states. The Mn–Mn and Mn–Mg distances increase between C5 and D3 and, like the Mn–O distance, return to similar values to those in pristine NMMO.

In contrast to the other scattering paths, the Mn–Na scattering distance continually decreases from C0 to C2 to D3; we attribute this decrease to a change in the equilibrium Na^+^ position (see the Discussion section). At point C5, scattering paths involving Na make no contribution to the EXAFS data observed, reflecting the low Na^+^ content in NMMO. Furthermore, the Debye–Waller factor for paths involving Na is significantly larger than those for O, Mn, and Mg, which likely reflects the comparatively high mobility and disorder of the Na^+^ ions.

### NMR Spectroscopy

**Figure 5:**
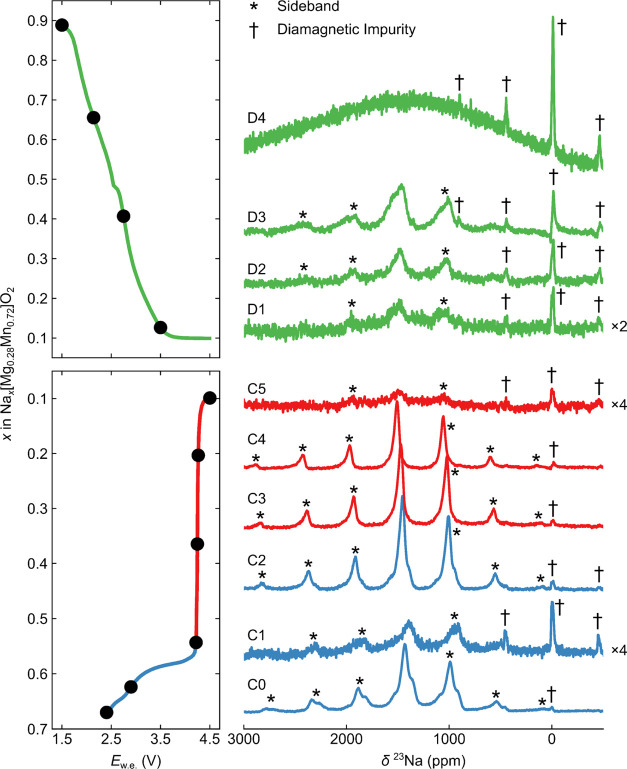
*Ex situ*^23^Na Hahn-Echo NMR spectra recorded for NMMO at different states of charge during the first charge–discharge cycle. Asterisks and daggers lie above or to the right-hand side of spinning sideband or diamagnetic impurity peaks, respectively. All spectra are normalized by the sample mass and number of scans; the intensities of the spectra at points C1, C5, and D1 have been increased to make the spectra more visible. The factor of these increases is shown to the right of these spectra; note that this has also increased the noise relative to other states of charge.

Having established the changes in the bulk structure during the first charge/discharge cycles of NMMO, we next went on to examine the local structural evolution using *ex situ*^23^Na NMR spectroscopy (Figure 5). Pristine NMMO exhibits three overlapping resonances at 1525, 1430, and 1360 ppm, confirmed in pjMATPASS experiments; this also confirmed that no additional resonances are hidden underneath the sidebands (Figures S22 and S23).

As depicted in the Supporting Information (Figures S28–S30), the hyperfine shifts calculated using the bond pathway analysis for the model P2 material Na_2/3_[Mg_1/3_Mn_2/3_]O_2_ (containing Mn^4+^ and no Mn^3+^) and the methodology outlined in previous publications^39−41^ range from −190 ppm to 3000 ppm, assuming a random arrangement of Mg^2+^ and Mn^4+^ ions, with these shifts depending on the number of Mg^2+^ and Mn^4+^ ions in the local coordination shells and on the Na–O–Mn bond angles (Figures S28, S29, and S30; Tables S3 and S4). Despite using the same basis sets and procedures as those run for similar layered NIB cathode materials,^80^ none of the calculated shifts of the P(2b) sites matched the observed resonances. The effect of second-order quadrupolar coupling on the shift, width, and shape of the peak was also accounted for: the calculated parameters lead to a width of approximately 20 ppm (noticeably smaller than the experimentally observed 80 ppm line width) and a shape that did not match the observed spectrum. The quadrupole-induced shifts, when added to the hyperfine shifts calculated, also did not improve the match to the shifts of the observed resonances (see Supporting Information; Table S2).

The first explanation for the discrepancy is that the Mg^2+^ and Mn^4+^ ions are at least partially ordered, as observed via XRD. This hypothesis was tested *via* a simple honeycomb-ordered model (see Figure S30, Table S4, and associated text), and now, only four resonances are generated at 1785–2108, 27–179, 591–713, and 1256–1247 ppm (the ranges resulting from the values generated when using different hybrid functionals in the calculations; see the Supporting Information). In this honeycomb-ordered model, the agreement with the experimental spectrum is better than the random model but still poor.

The second explanation for the discrepancies is that the resonances seen experimentally arise from Na^+^ ions exchanging between sites within the layers: as observed for other layered NIB and LIB cathodes,^80−82^ the line shape of the observed spectra for NMMO during charge and discharge will be strongly affected by Na^+^ ion mobility on the timescale of the NMR experiment (specifically the ^23^Na hyperfine interaction). Hopping during the NMR experiment has two effects: first, the broadening and coalescence of the resonances corresponding to individual ^23^Na sites and second, additional spin–spin (*T*_2_) relaxation effects when the hopping frequency and difference in resonant frequencies are similar in size. When hops between sites are fast compared to the difference in the resonant frequencies of these sites, a sharp, coalesced feature is seen at a shift which is the thermodynamic average of the shifts of the sites between which Na^+^ ions hop (the fast regime; the thermodynamic average accounts for the relative energies and energy barriers to Na^+^ ions hopping between two sites). When hops are slower, either a single, broadened, low-intensity feature is seen (the intermediate regime) or two features corresponding to the two sites between which Na^+^ hops are seen (the slow regime).

To account for this motional effect, we determined the weighted average of the calculated shifts of the four resonances generated in the ordered honeycomb model (with shifts and probabilities given in Table S6). This produced shifts between 1307 and 1501 ppm (the range again depending on the choice of the functional), which is in very good agreement with the shifts for the three overlapping resonances seen experimentally and provides further evidence for cation ordering within the TMO_2_ layers.

To demonstrate the effect of Na^+^ ion motion on the NMR timescale, we first attempted room-temperature two-dimensional NMR exchange spectroscopy experiments. These were not successful, likely due to the short relaxation times in this system. We, therefore, carried out variable-temperature ^23^Na NMR experiments (Figures S25 and S26), which, for the pristine material, revealed a gradual sharpening of the resonances at higher temperatures and broadening and decrease in intensity at lower temperatures, consistent with Na^+^ ions going from the fast regime (at 378 K) to the intermediate regime (at 288 K). Although changes in the relative intensity occur, the resonances do not coalesce at high temperatures, suggesting that not all of the Na^+^ ions are exchanged, perhaps because the different resonances correspond to different layers of Na^+^ ions, each with different distributions of Mg^2+^, Mn^3+^, and Mn^4+^ cations, for example, Na^+^ ions sitting in layers with the AA-honeycomb stacking of the nearby TMO_2_ layers; Na^+^ sitting in layers with the AB-honeycomb stacking; and Na^+^ in layers with a “random” arrangement of TM cations in the adjacent TMO_2_ layers. Alternatively, Na^+^ ions may be in a partially ordered array, perhaps related to the observed superstructure, with each resonance corresponding to three distinct modes of Na^+^ ion motion (which depends on the number of vacant Na^+^ ion sites nearby). Establishing exactly which sites Na^+^ ions hop between and the ordering of TM cations in the adjacent TMO_2_ layers—and therefore establishing which resonance corresponds to different Na^+^ ion hopping routes—is beyond the scope of this work because it requires detailed analysis and simulation of these variable-temperature spectra and analysis of the energetics of the different hop pathways between the multiple Na environments. Instead, we describe the changes in the *ex situ*^23^Na NMR spectra with the state of charge and use these spectra to infer changes in the mobility and local structure. The results are then related to the *ex situ* XRD and EXAFS data and the Mg^2+^ migration calculations in the Discussion section.

The general trend seen on charging from C0 to C4 is a gradual increase in the ^23^Na isotropic shift, consistent with other members of the Na_*x*_Mg_*y*_Mn_1–*y*_O_2_ family^24^ and suggestive of a gradual change to the electronic structure of NMMO during the charge (*i.e.*, Mn oxidation from Mn^3+^ to Mn^4+^) and/or a gradual change in the energies of the different Na sites within the layer (causing a gradual change in the exchange-averaged shift). In general, the observed resonances also became sharper during the charge. Exceptions to these trends occur at 2.90 V on the first charge (C1 in Figure 5) and at the end of the first charge (C5); the spectra for these points have been magnified in Figure 5 for the sake of clarity. Repeated measurements of all points along the charge–discharge curve were carried out; these also showed the same sharp resonances at C0, C2, C3, and C4 and broad resonances at C1 and C5. We therefore assign these broadened, low-intensity features to slower Na^+^ motion (C1 and C5) and/or low Na^+^ content (C5) rather than low-quality samples or spectral artefacts. The variable-temperature spectrum of C1 (Figure S26) shows that a sharp series of resonances emerges at 378 K, consistent with our proposal that the motion at room temperature is slower for this sample than that for C0 and C2–C4. The resonances seen at points C1 and C5 also show a slightly lower shift, likely due to a change in the energy barriers associated with Na^+^ ion hopping, which results in both different site occupancies and also Na^+^ ions that are not detected in these Hahn-echo experiments (see below).

**Figure 6:**
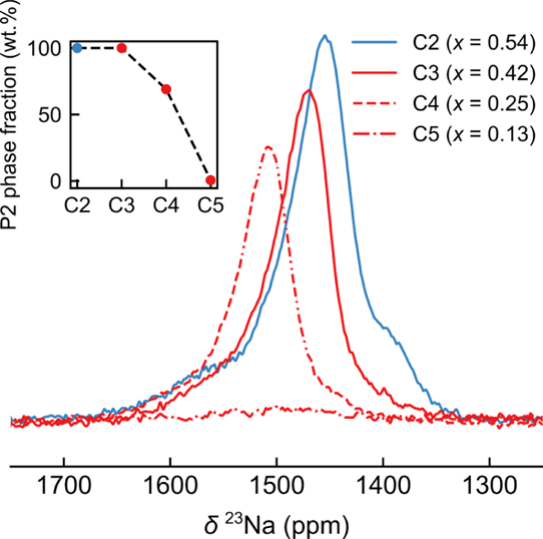
Variation in the intensity of the isotropic resonances in the ^23^Na NMR Hahn-echo spectrum of NMMO samples at the high-voltage charge plateau (the values of *x* in the legend correspond to the composition Na_*x*_[Mg_0.28_Mn_0.72_]O_2_), with the inset showing the variation in the P2 phase fraction along the charge plateau (obtained from Rietveld refinement of the *ex situ* XRD patterns). The spectra were acquired at the same spinning speed and using the same recycle delay, set to at least 5 times the longitudinal relaxation time (*i.e.*, *d*_1_ > 5*T*_1_), and spectra have been scaled by sample mass and the number of scans to allow a quantitative comparison of the intensities.

Unlike other members of the Na_*x*_Mg_*y*_Mn_1–*y*_O_2_ family, however, a distinct, broad resonance at 1100 ppm, corresponding to the O-type Na^+^ environments of the OP4 phase of Na_*x*_Mg_*y*_Mn_1–*y*_O_2_,^24^ was not observed for NMMO, suggesting that a distinct (ordered) OP4 phase does not form—which in turn suggests that the *Z* phase is a more appropriate description of the high-voltage phase. Furthermore, that no discrete peak is observed when OP4 starts to emerge (e.g., in C4) provides compelling evidence that no (or very few) Na^+^ ions occupy the O-type layers. It is possible that Na^+^ ion motion in the O-type layers of OP4-NMMO are in the intermediate regime, where no distinct resonances are seen, due to the severe broadening of the signal. However, the decrease in intensity seen on charging NMMO from C2 to C5 correlates with the decrease in the P2 phase fraction (Figure 6), so we assign this signal to Na^+^ ions in P-type layers, rather than O-type. Since the phase fraction of P2 is low at the end of first charge (*ca.* 1%), we attribute this ^23^Na NMR resonance to Na^+^ ions now in the P-type layers of the *Z* phase (or in our O2-OP4 surrogate model, the P-type layers of the OP4 phase).

The shoulders to higher and lower frequencies of the main signal in the ^23^Na NMR spectra decrease in intensity during the charge, while the main resonance sharpens and increases in intensity. This phenomenon suggests that there is either preferential Na^+^ extraction—with Na^+^ ions giving rise to the shoulder peaks being removed before Na^+^ ions which give rise to the central resonance—and/or an increase in the relative energies of these sites (leading to a lower population of these Na^+^ sites) or that the rate of exchange between the sites increases as the Na^+^ ions are extracted. Likely, all these phenomena occur. Of note, the position of the weak signal that remains in the spectrum of C1 coincides with the low-frequency shoulder seen in the C0 spectrum, suggesting that the Na^+^ ions corresponding to the high-frequency shoulder and central resonances have entered the intermediate motional regime. The ^23^Na signal at C2 is more intense than that at C1, a consequence of faster Na^+^ ion motion—*i.e.*, C1 is in the intermediate regime, while C2 is in the fast regime. The spectrum at C2 also contains weaker shoulder peaks, which weaken as more Na^+^ ions are removed. Since the structural changes between C0 and C1 are minor and the (2 × 2 × 1) superstructure reflections are still present in the diffraction patterns of C1 (Figure S7), the decrease in the ^23^Na signal intensity is tentatively associated with increased Na^+^/vacancy ordering—possibly enabled by the removal of the first few Na^+^ ions on charging—leading to slower overall Na^+^ ion motion. It is possible that the broadening of the spectra is also associated with the onset of a disruption of electronic (Mn^3+^/Mn^4+^) ordering that likely occurs around this composition.

On discharge, the isotropic resonances remain broad, suggesting slower Na^+^ motion on discharge than that on charge, possibly related to residual Mg^2+^ ions in the Na^+^ layers, as proposed based on diffraction results and explored computationally below. The features observed on discharge are also likely broadened due to the presence of stacking faults and increased structural disorder at short length scales, as observed in XRD. The observed resonances for these states of charge also remain at slightly higher shifts than those seen on charge, indicating different local environments and hopping barriers for Na^+^ ions, which perhaps correspond to Na^+^ ions in O-type layers with residual Mg^2+^ ions present and/or Na^+^ ions in P-type environments whose electronic structure differs from the original P2 phase.

### Calculations of Mg^2+^ Migration Barriers

**Figure 7:**
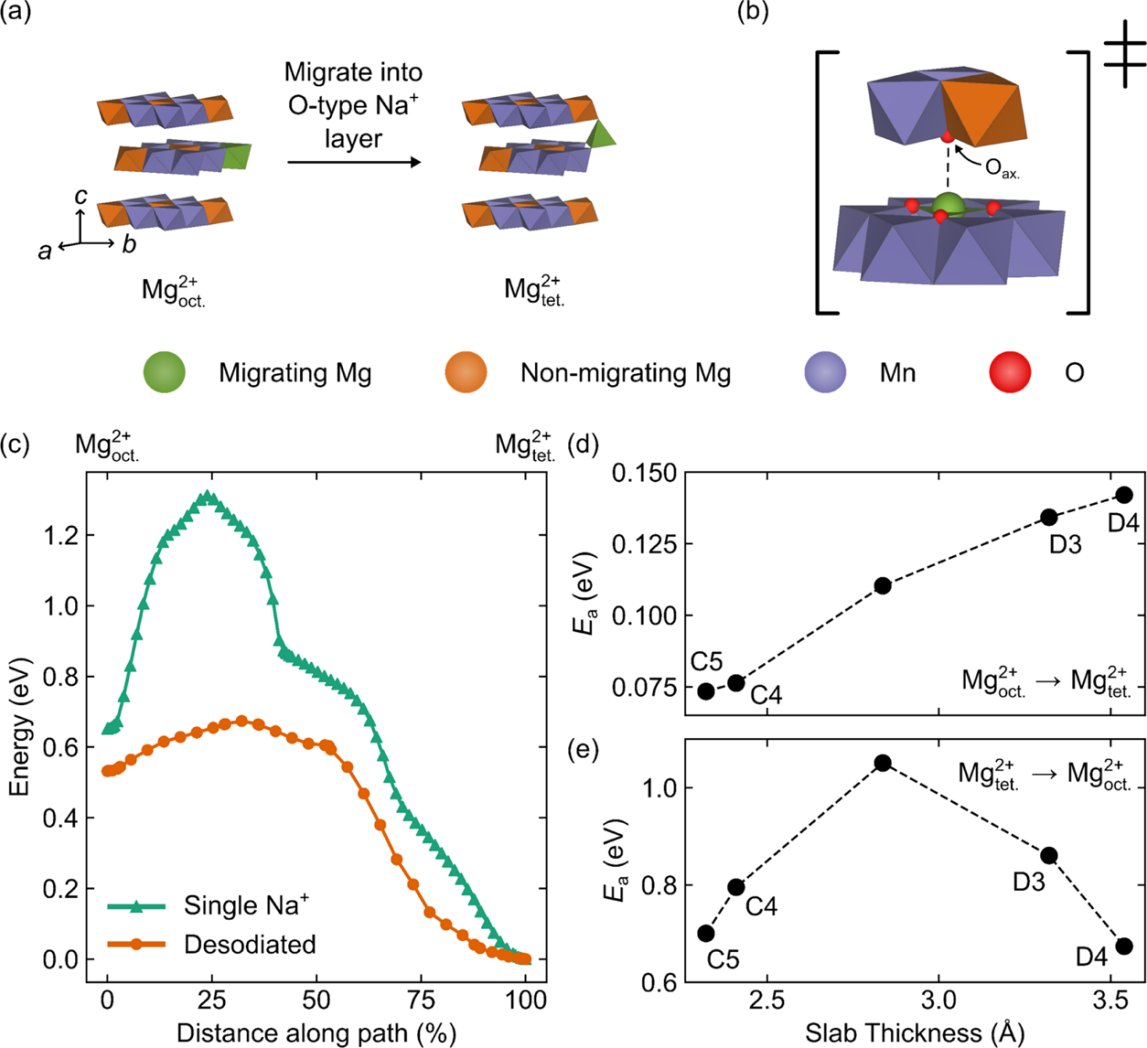
(a) Schematic Mg^2+^ migration mechanism in O2-NMMO, where Mg^2+^ moves between an octahedral site in the TMO_2_ layer (Mg^2+^_oct._) and a tetrahedral site in the O-type Na^+^ layer (Mg^2+^_tet._). (b) Expanded view of the TS of this mechanism, with the axially coordinated O^2–^ ion (which stabilizes the migrating Mg^2+^ in the TS), O_ax_, labeled. (c) Energy profiles for Mg^2+^ migration in O2-NMMO cells with one Na^+^ per O-type layer into which Mg^2+^ migrates (“single Na^+^”) and no Na^+^ (“desodiated”) in the O-type Na^+^ layer into which Mg^2+^ migrates. (d,e) Variation of the activation energy barriers for the migration of Mg^2+^ in O2-NMMO from Mg^2+^_oct._ to Mg^2+^_tet._ and from Mg^2+^_tet._ to Mg^2+^_oct._, respectively, as the O-type Na^+^ slab thickness changes. All but one cell size was based on the cell sizes obtained from *ex situ* XRD Rietveld refinements.

While both the *ex situ* XRD and EXAFS data are consistent with Mg^2+^ having migrated to tetrahedral sites in O-type layers, they do not provide conclusive proof. Thus, to identify whether this proposed migration is energetically (thermodynamically and kinetically) feasible, we next calculated the diffusion profiles and activation energy barriers for Mg^2+^ migration in O-type layers using single-ended TS searches for a series of O2 and OP4 models (Figures 7 and S31–S35). While the true structure of NMMO along the charge plateau is a *Z* phase, it was not possible to run TS searches over the length scale required to adequately define a *Z*-phase unit cell; therefore, we have again focused on the separate O2 and OP4 layer slabs which make up the *Z* phase by performing calculations on (2 × 2 × 1) supercells of the O2 and OP4 phases.

In all cases, the migration pathway consisted of Mg^2+^ moving between an octahedrally coordinated site in the TMO_2_ layer (Mg^2+^_oct._) and a tetrahedrally coordinated site in the O-type Na^+^ layer (Mg^2+^_tet._) *via* a TS in which Mg^2+^ occupies a trigonal planar (or approximately planar) coordination environment (Figure 7a,b). In all cases, our TS searches revealed that the Mg^2+^_tet._ configuration was considerably lower in energy than the Mg^2+^_oct._ configuration, akin to the selective occupation of tetrahedral Mg^2+^ sites in MgMn_2_O_4_ spinels.^61,83^ The barrier for Mg^2+^ migration was found to depend on the presence of Na^+^ near the migrating Mg^2+^, the lattice parameters of the cell and the presence of stacking faults.

In the absence of Na^+^, a typical barrier for Mg^2+^ migrating from Mg^2+^_oct._ to Mg^2+^_tet._ was extremely low: approximately 75 meV for the O2 phase and 70 meV for the OP4 phase (for a Na^+^ layer slab thickness of 2.3 Å; Figure 7d and S32c), indicating that Mg^2+^ migration is feasible when the O-type layer into which Mg^2+^ migrates is empty. The reverse process—Mg^2+^ migrating from Mg^2+^_tet._ to Mg^2+^_oct._—has a significantly higher energy barrier: approximately 700 meV for the O2 phase and 500 meV for the OP4 phase, primarily because the final state is higher in energy. When Na^+^ occupies the O-type layer (composition Na_0.56_[Mg_0.28_Mn_0.72_]O_2_), the barrier for Mg^2+^ migrating from Mg^2+^_oct._ to Mg^2+^_tet._ increases to at least 110 meV for the O2 phase (Figures 7c and S33) but is still well within the range of overpotentials applied to the material during cycling and results in an overall lowering of the energy of O2-NMMO by approximately 1 eV. The inflexion seen at approximately 50% along the reaction pathway for Mg^2+^ migrating into the O-type Na^+^ layer containing one Na^+^ (Figure 7c) corresponds to the movement of Na^+^ in the O-type layer from its octahedral site into a tetrahedral site (further from the migrated Mg^2+^), with the rest of the path corresponding to the movement of the same Na^+^ ion into a new octahedral site further from the migrated Mg^2+^. Most of the Na^+^ ion motion occurs after Mg^2+^ has migrated; the Na^+^ ion moves by less than 0.5 Å (*i.e.*, less than half of the ionic radius of Na^+^^84^) as Mg^2+^ migrates. The increase in both barriers (Mg^2+^_oct._ to Mg^2+^_tet._ and vice versa) arises from the increase in Coulombic repulsion between Mg^2+^ and the nearby Na^+^ in the TS: additional TS searches indicated that as the Mg^2+^–Na^+^ distance decreased, the barrier increased significantly (Figure S33). TS searches with Na^+^ in the O-type layer into which Mg^2+^ migrates could not be converged for the OP4 system, presumably due to the flat potential curve associated with Mg^2+^ migration in this system. Searches for the O2 system will also likely have relatively flat potential curves but not so flat that a TS could not be found.

To reflect the changes in the O2 and OP4 lattice parameters obtained from *ex situ* XRD (a consequence of Na^+^ deintercalation), we investigated the effect of cell size on the barriers to Mg^2+^ migration (Figure 7d,e). While we have plotted changes in the Na^+^ layer slab thickness in Figure 7d,e, we highlight that both the *a* and *c* lattice parameters were varied to understand how cell size affects the barriers (*i.e.*, for each cell size, a separate, fixed-cell calculation was performed). The *a* and *c* parameters used were taken from the Rietveld-refined parameters at points C4, C5, D3, and D4, with an extra point with *a* and *c* parameters at the average of D1 and D4 (to account for cell sizes between these points) for the O2 phase and points C5, D2, D3, and the average of C4 and D3 (again, chosen to account for the spread of cell sizes observed) for the OP4 phase; these states of charge are indicated on Figures 7, S31, and S32, where appropriate. Note that changes in *c* dominate changes in the cell size, and thus, our presentation of the results and subsequent discussion will proceed in terms of changes in the Na^+^ layer slab thickness for the sake of clarity. For the O2 and OP4 phases, as the Na^+^ slab thickness increases, the energy barrier to migration from Mg^2+^_oct._ to Mg^2+^_tet._ increases approximately linearly (Figure 7b). This likely arises from the loss of stabilization of the trigonally coordinated Mg^2+^ TS by the oxygen atom opposite Mg^2+^, O_ax_, due to the increase in the Mg–O_ax_ distance. For the thinnest slab, the Mg–O_ax_ distance in the Mg^2+^_tet._ configuration was 1.93 Å, while the thickest slab had a Mg–O_ax_ distance of 2.41 Å; for comparison, a typical Mg_tet_–O distance is 1.9–2.0 Å (Figure S31).^85,86^

The barrier to migration from Mg^2+^_tet._ to Mg^2+^_oct._ in the O2 phase initially increased as the Na^+^ layer slab thickness increased but then decreased for thicker slabs (Figure 7e). We attribute this to the increased stabilization of Mg^2+^_tet._ at intermediate Mg–O_ax_ bond lengths: shorter bonds increase electrostatic repulsion, while long bonds have a weaker spatial overlap of orbitals and therefore increase the overall electronic energy. The OP4 phase had thinner Na^+^ slabs than the O2 phase so that as the slab thickness increased, the barrier to migration decreased; since the thinner slabs have the TMO_2_ layers closer together, it is likely that the decrease in the barrier arises from an increase in the stability of the TS due to less electrostatic repulsion between the TMO_2_ layers (Figure S32). We anticipate similar behavior to that of the O2 phase when these slab sizes are comparable, but calculations on these states could not be converged, again likely due to the shallow potential curves for this system.

Finally, when AA/AB stacking faults are introduced (*i.e.*, when the Mg and Mn positions differ between layers), the activation energies in the O2 phase increase, while the activation energies in the OP4 phase remain approximately constant (Figure S34).

Thus, in the *Z* phase, which contains a mixture of O2 and OP4 domains, the barriers to Mg^2+^ migration are predicted to increase when Na^+^ ions occupy the O-type layer, the *c* axis extends, and when AA/AB stacking faults occur.

We also carried out TS searches on the migration process involving Mg^2+^ moving from the tetrahedral site in the O-type layer to an adjacent octahedral site in the same O-type layer (since this is the most likely route for Mg^2+^ to take to migrate from the octahedral site in the TMO_2_ layer to the octahedral site in the Na^+^ layer). A typical energy barrier for a hop from the tetrahedral site to an octahedral Na^+^ site was approximately 900 meV, and that of the reverse (from an octahedral Na^+^ site to a tetrahedral site) was approximately 50 meV (Figure S35), the difference reflecting the 860 meV higher energy associated with the occupancy of the octahedral site in comparison to that of the tetrahedrally coordinated Mg^2+^.

Therefore, our *ab initio* TS searches reveal that Mg^2+^ migration is thermodynamically favorable (*i.e.*, a lower energy structure is generated) and kinetically accessible (*i.e.*, it involves a single ion hop whose barrier is within the range of overpotentials applied along the charge plateau of NMMO). Clearly, the bulk and local structures of NMMO change during the first charge–discharge cycle, as seen in *operando* and *ex situ* XRD, *ex situ* EXAFS, and ^23^Na NMR spectra. These changes are the origin of the voltage hysteresis seen in NMMO.

## Discussion

A large voltage hysteresis was observed during the first charge–discharge cycle of NMMO. This voltage hysteresis represents a significant energy loss during electrochemical cycling, which can lead to low efficiencies and lifetimes. To provide strategies that might help mitigate this hysteresis, we have investigated both the bulk and local structural changes taking place in NMMO during the first charge–discharge cycle, where the hysteresis is the greatest; subsequent cycles showed a more sloping voltage profile with a smaller voltage hysteresis (Figure S3), consistent with many oxygen-redox-based materials.^87−91^ The large voltage hysteresis during the first charge–discharge cycle suggests the presence of different phases during charge and discharge, evidence for which is seen in XRD, Mn *K*-edge EXAFS, and ^23^Na NMR spectra.

### Bulk and Local Structure of Pristine NMMO

Pristine NMMO adopts a P2 structure, with Mg^2+^ cations showing a preference to sit at TM sites at the corners of the unit cell to form a honeycomb-like arrangement where they are surrounded by Mn^3+/4+^ ions only (in the TMO_2_ layers). All available Na^+^ sites in the Na layers are partially occupied, providing pathways and mechanisms for Na^+^ ion motion. The ^23^Na NMR spectrum was consistent with the honeycomb ordering and, furthermore, indicated fast Na^+^ ion hopping on the NMR timescale, with resonances observed at shifts which are the thermodynamic average of the shifts of the sites between which Na^+^ hops (see Supporting Information Section 9, Table S6). EXAFS also suggested high Na^+^ ion mobility due to the large Debye–Waller factors for Na-based scattering paths.

### Structural Evolution during Charge

**Figure 8:**
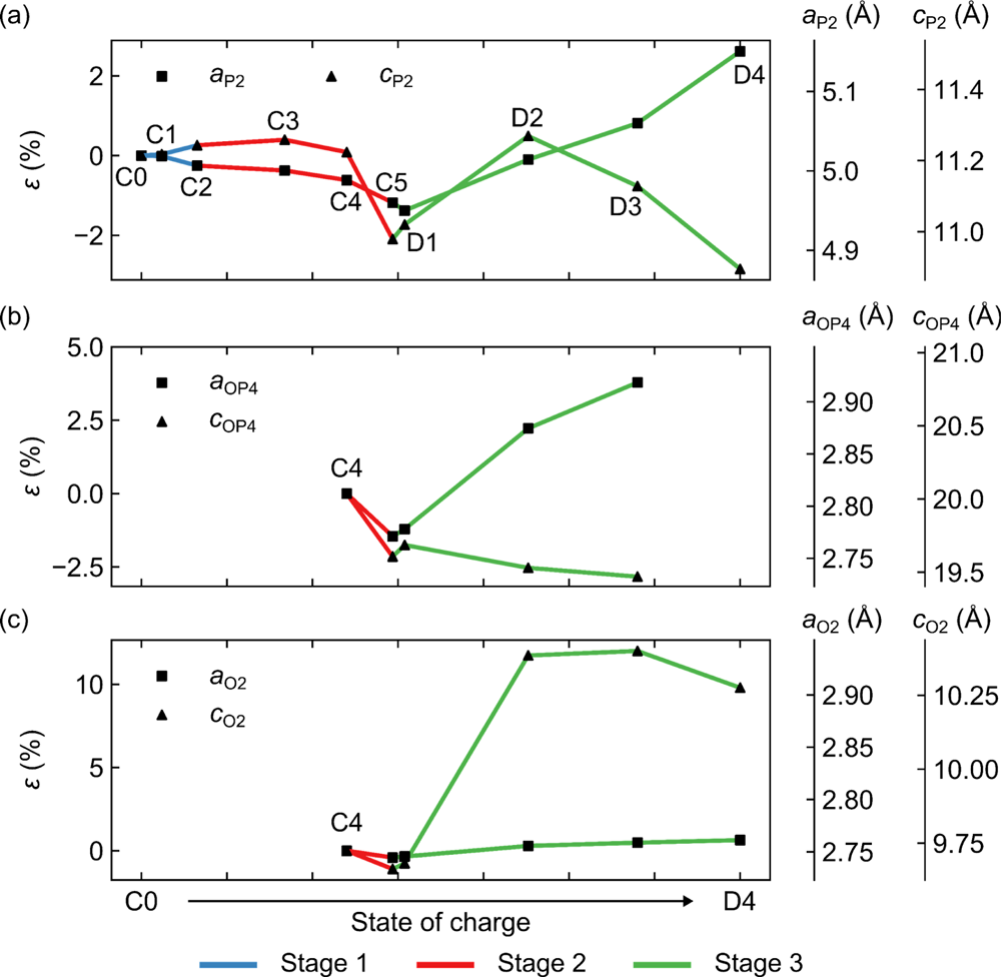
Rietveld-derived lattice parameters for (a) the P2 phase, (b) the OP4 phase, and (c) the O2 phase at different states of charge. Note that the OP4 and O2 phase do not appear until point C4 (highlighted) and the OP4 phase disappears after point D3.

During stage 1 (charge from pristine up to 4.22 V vs Na^0/+^), NMMO undergoes a single-phase reaction, with the lattice parameters responding dynamically to Na^+^ extraction: the *a* axis shortens due to Mn–O oxidation, while the *c* axis lengthens due to the loss of Na^+^ ions from between the TMO_2_ layers, resulting in greater electrostatic repulsion between the layers (Figure 8a). EXAFS data confirm the decrease in the Mn–O distance, which also leads to a decrease in the Mn–Mg distance. The Mn–Mn distance increases during stage 1, which we attribute to the increased Coulombic repulsion between Mn centers due to the increase in the average oxidation state.

The EXAFS data also indicate a decrease in the Mn–Na distance between points C0 and C2, despite the expansion in the *c* axis seen in XRD results. We attribute the decrease in this scattering distance to a change in the equilibrium position of Na^+^ with respect to the TMO_2_ layer. The distance between Mn and its nearest neighbor Na^+^ will vary according to the local environment. Each Na^+^ P(2d) and P(2b) site can have a different local environment: for example, Na^+^ can occupy a P(2b) site which shares two, one, or no faces with Mg^2+^, or it can occupy a P(2d) site with no, 1, 2, 3...up to 12 Mg-nearest neighbors. A decrease in the Mn–Na distance between C0 and C2 suggests that the equilibrium Na^+^ position moves closer to Mn.

While pristine NMMO shows sharp, distinct resonances in its ^23^Na NMR spectrum, when charged to 2.90 V (*i.e.*, point C1), a broad, low-intensity feature is seen, suggesting slower Na^+^ hopping compared to that of the pristine material, perhaps due to partial Na^+^/vacancy ordering. The composition at C1 is *x* ≈ 5/8. As NMMO has six Na^+^ sites per Na^+^ layer per unit cell, ordering schemes consistent with this composition correspond to a Na^+^/vacancy ordering schemes over four unit cells with 15 Na^+^ sites occupied, examples of which are illustrated in Figure S37. Once formed, the energy barrier to Na^+^ hopping will increase as any move away from the low-energy, ordered array of Na^+^ ions and vacancies (*i.e.*, the local thermodynamic minimum) will increase the total electrostatic repulsion between Na^+^ ions. This will lead to slower Na^+^ ion motion (*i.e.*, the intermediate regime) and broad ^23^Na NMR resonance. Indeed, vacancy ordering at *x* = 5/8 is known to occur readily in Na_*x*_MnO_2_ (the parent material).^92,93^ At this point, a sharp peak in the d*Q*/d*V* profile is also seen (Figure 3f), likely corresponding to Mn^3+^ oxidation, perhaps indicating (some) degree of Na^+^/vacancy ordering. No new peaks (corresponding to a new superstructure) were, however, observed in the *ex situ* and *operando* XRD data, but the pristine superstructure peaks remained.

On charging to point C2 (the beginning of the charge plateau), the composition reaches *x* ≈ 0.54, corresponding to 13 Na^+^ per layer of four unit cells; here, sharp resonances are seen, consistent with fast motion, due to the increase in the number of Na^+^ vacancies. The faster Na^+^ motion is also reflected in the Debye–Waller factors for the Mn–Na scattering paths, which increase between the beginning (C0) and the end (C2) of stage 1.

Stage 2 comprises the voltage plateau during charge. While a flat voltage region should reflect a two-phase reaction, evidence for this is not observed in *ex situ* or *operando* XRD spectra until over halfway along the plateau (point C4), where a new, broad peak is observed, corresponding to reflections from the TMO_2_ layers in the *Z* phase (a disordered intergrowth of the O2 and OP4 phases). In the *Z*-phase material, the O-type layers are Na^+^-deficient—as seen in the ^23^Na NMR spectra, which contain signals from the P-type layers only (Figure 6)—and, on the basis of the calculations, contain migrated Mg^2+^ ions in tetrahedral sites.

During stage 2, XRD refinement of the P2 phase reveals a decrease in the *a* axis (again consistent with Mn–O oxidation) and an initial increase in the *c* axis, followed by a sharp decrease at the end of charge (point C5). Likewise, the O2 and OP4 phases show a decrease in the *a* and *c* axes from point C4 to C5 (Figure 8b,c). The initial increase in *c* for the P2 phase continues the trend seen in stage 1: loss of Na^+^ decreases the shielding of negative charges on the TMO_2_ layers, resulting in greater electrostatic repulsion between the layers. The large decrease in *c* for the O2 and OP4 phases at the end of charge may reflect the same phenomenon as that seen in layered LIB materials, where a “collapse” of the TMO_2_ layers has been seen.^81,94,95^ However, here, an additional cause of the collapse in *c* at these high voltages is ascribed (at least in part) to Mg^2+^ migration as the charges on the TMO_2_ layers will be effectively shielded by Mg^2+^; we also anticipate that the small tetrahedral Mg^2+^ (compared to octahedral Na^+^) will facilitate layer collapse. Therefore, we expect that the high voltage capacity observed in NMMO is coupled with Mg^2+^ migration, analogous to metal cation migration seen in some O-redox-active LIB cathodes.^91,96^

The Mn *K*-edge EXAFS data also revealed a decrease in the Mn–O distance along the charge plateau, as well as the presence of Mg^2+^ in the O-type Na^+^ layers at the end of the first charge.

Despite the nucleation and growth of the *Z* phase along the high-voltage plateau, no new ^23^Na NMR resonances are observed, suggesting that the remaining Na^+^ ions in the *Z* phase reside primarily in the residual P-type layers, where the local environments remain essentially the same as those in P2-NMMO; this also confirms the observation that no Na^+^ scattering contributes to the EXAFS at point C5. The slight increase in the isotropic ^23^Na NMR shift seen along the charge plateau, as well as the sharpening of the resonances along this plateau, most likely arises from an increase in Na^+^ ion mobility in the P-type layers and residual Mn oxidation.

The ^23^Na NMR spectra showed a decrease in the intensity of the shoulder peaks during charge (C2 to C5), while the central resonance becomes sharper and more intense, suggesting that Na^+^ is preferentially extracted from the sites that give rise to the shoulder peaks and/or the remaining Na^+^ ions spend more time hopping between the sites which correspond to the central resonance. The shoulders could also arise from differences in the honeycomb stacking between the TMO_2_ layers (as seen in Li_2_MnO_3_, e.g.^97^), with Na^+^ ions being more readily extracted from some stacking sequences.

### Structural Evolution during Discharge

The beginning of discharge consists of a sharp decrease in voltage (from 4.5 V to approximately 3.8 V) with little change in Na^+^ content. We ascribe this drop in voltage to the reversal in the sign of the internal resistance (*iR* drop) and reversal of the sign of overpotentials between charge and discharge, as seen in additional galvanostatic intermittent titration technique (GITT) data (Figure S39), along with the onset of sodiation.

Between points C5 and D1 (the beginning of stage 3), very little change in the diffraction patterns and ^23^Na NMR spectra are seen; the O2 and OP4 lattice parameters also remain approximately constant, but the phase fraction of OP4 increases at the expense of the O2 phase, suggesting that some rearrangement of the O-type and P-type layers in the *Z* phase takes place, which is likely a “relaxation” of the structure. Since a small amount of Na^+^ is inserted into NMMO between C5 and D1 (approximately 0.03 equivalents), some O-type layers may transform back to become P-type, thereby increasing the fraction of the OP4 phase. As both electrodes were relaxed for at least 1 h after charging (C5) or discharging (D1), we anticipate these rearrangements to occur during discharge, rather than during the rest period.

The rest of discharge (points D2 to D4 inclusive) is described by a sloping voltage profile, suggesting single-phase Na^+^ insertion. The *ex situ* and *operando* diffraction patterns indicate a gradual increase in the P2 phase fraction at the expense of the O2 and OP4 phases, suggesting that the *Z* phase gradually transforms back into the P2 phase, presumably *via* insertion of Na^+^ into the O-type layers and subsequent slippage of these O-type layers to become P-type.

Furthermore, the *a* lattice parameter for all phases increases throughout discharge, and at point D3 (whose composition is approximately *x* = 0.66), the Mn–O scattering distance returns to almost the same length as that in the pristine material. The *c* lattice parameter for the P2, OP4, and O2 phases also increases initially on discharge due to insertion of the large Na^+^ ions; in the case of the O2 phase, this expansion is very large, presumably because the O-type layers were devoid of Na^+^ on charge but must accommodate the large Na^+^ ions on discharge—*i.e.*, the Na^+^ layers dominate the expansion in *c* for the O2 phase. In the ^23^Na NMR spectrum, a set of broad, overlapping resonances are seen between 1380 and 1650 ppm. Since the O2 and OP4 phase fractions at D3 are approximately 1.0(10) and 0.5(10)%, respectively, we do not anticipate that the O-type Na^+^ environments in these phases will contribute significantly to the ^23^Na NMR spectrum observed. We ascribe the broadening of these resonances and the low intensity in part to the low crystallinity and wide distribution of local environments and also to the relatively slow Na^+^ hopping.

By point D3, the *c* axis for the P2 (98.5(2)% phase fraction) and OP4 (1.00(10)%) phases begins to decrease, while for the O2 phase (0.51(14)%), *c* remains approximately constant. The contraction in *c* for the P2 and OP4 phases likely originates from the large number of Na^+^ ions between the TMO_2_ layers: as more Na^+^ ions are inserted between the TMO_2_ layers, the repulsive electrostatic interactions decrease, enabling the TMO_2_ layers to move closer together, in a fashion analogous to that of layered LIB cathodes.^81^ These repulsive interactions are anticipated to be greatest in the P-type layers, where O^2–^ anions are eclipsed rather than staggered; as a result, the P2 phase contracts the most, followed by OP4, while the O2 phase sees little change in *c*.

By the end of stage 3 (points D3 and D4), negligible amounts of the O2 and OP4 phases remain, suggesting that most of the *Z* phase has transformed back to a P2-like phase, with residual O-type layers “trapped” as stacking faults. These stacking faults are also captured in the (002) reflection, which is broader and lower in intensity than that of P2-NMMO during stages 1 and 2.

In addition to the residual O-type layers, the P2 phase generated on discharge is more disordered compared to the P2 phase on charge, as suggested from the broadened Bragg peaks. The (100) reflection—diagnostic of ordering over the TM sublattice—has a much lower intensity than that on charge, indicating that the partial honeycomb order in the TMO_2_ layers of pristine NMMO is lost on cycling. The origin of this loss of order is likely from residual Mg^2+^ ions in O-type layers (and therefore vacancies in the TMO_2_ layers). An additional source of disorder may also arise from inter- and intralayer Mn^*n*+^ migration, where Mn^*n*+^ cations move from their octahedral sites in the TMO_2_ layer to neighboring vacant octahedral sites also in the TMO_2_ layers, created from Mg^2+^ migration. When Mg^2+^ migrates back to the TMO_2_ layer on discharge—most likely driven by electrostatic repulsion from Na^+^ being inserted into these O-type layers—it must move into the new vacant site created by Mn^*n*+^ migration, which disrupts the partial Mn/Mg honeycomb order seen in the pristine material.

The increase in the structural disorder of NMMO during discharge is also borne out in the EXAFS data: the Mn–O, Mn–Mn, and Mn–Mg scattering distances at point D3 return to similar values to those of the pristine material but with much larger Debye–Waller factors, indicating greater variability (*i.e.*, more disorder) in these distances. The Mn–Na distance at D3, however, is significantly shorter than that in the pristine material, suggesting a change in the equilibrium position for Na^+^. This is reflected in the ^23^Na NMR spectra during stage 3, where broad, low-intensity features are observed at shifts that are slightly higher than those observed on charge—indicating different local ^23^Na environments, greater local disorder, and slower Na^+^ ion motion. By the end of the first discharge, the ^23^Na NMR spectrum becomes severely broadened due to the large distribution of local sites which are occupied.

Our *ab initio* single-ended TS searches show that the barrier for Mg^2+^ migration from Mg^2+^_tet._ back into the TMO_2_ layer is significantly larger than the barrier for migration into Mg^2+^_tet._ sites. Therefore, the thermodynamic driving force required for Mg^2+^ to migrate back to Mg^2+^_oct._ will be larger than that required for Mg^2+^ to migrate into the Mg^2+^_tet_ sites, suggesting that on discharge, Mg^2+^ will not migrate back to Mg^2+^_oct._ at the same potentials that Mg^2+^ migrated to Mg^2+^_tet._ on charge. Consequently, we expect Mg^2+^ migration to contribute to the observed voltage hysteresis.

### Implication of Results and Outlook

Recent studies on Li-rich NMC layered cathodes^96^ and on layered Li_2_Sn_*y*_Ir_1–*y*_O_3_ cathodes^91^ have suggested that metal migration and de-coordination (or undercoordination) of oxide anions during charge may promote oxygen redox and stabilize oxidized oxide ion species, for example, by forming TM–O π bonds.^91,96,98^ Our results suggest that Mg^2+^ migration likely plays a significant role in the charge–discharge behavior of NMMO: not only does it affect Na^+^ hopping kinetics, but it also influences the bulk structure of NMMO, which in turn will likely influence the electronic structure and the ability of NMMO to stabilize oxidized O species.^99^

In previous studies, the ionic bonds between Mg^2+^ and O^2–^ were proposed to generate high-energy electronic states on O which could be readily depopulated to give O redox.^28^ If Mg^2+^ migrates into the Na^+^ O-type layers, however, we anticipate the Mg–O bond to become much more covalent in character and for some of the O^2–^ ligands to become underbonded. In the regions of the material where the Mn^*n*+^ and Mg^2+^ ions are honeycomb-ordered, the migration of Mg^2+^ will generate “rings” of Mn^*n*+^ ions in the TMO_2_ layer, which may enable redox-active delocalized π systems to form, akin to those proposed by Kitchaev and co-workers as the source of high voltage capacity seen in some supposed O redox materials.^100^

The results presented here highlight that the suppression of Mg^2+^ migration through careful material design—for example, by using dopants in the TMO_2_ layers which have strong octahedral site preference (such as Ni^2+^ and Cr^3+^) or using dopants with large cationic radii (such as Sr^2+^) to encourage “pillaring” throughout the structure^101^ —may be important for decreasing the hysteresis in this promising class of high-capacity NIB cathodes.

Future ^25^Mg NMR and theoretical studies of the effect of Mg^2+^ migration on the chemical and electronic structure of NMMO will be carried out to review the ionicity and covalency of both the Mg–O and Mn–O interactions to assess the extent to which O redox behavior and Mg^2+^ migration are coupled.

## Conclusions

In this work, we have presented the bulk and local structural changes which take place during the first charge–discharge cycle of a layered NIB cathode, Na_0.67_[Mg_0.28_Mn_0.72_]O_2_, NMMO.

On charging NMMO, we observed single-phase Na^+^ extraction, followed by a two-phase reaction between the P2 and *Z* phases. The *Z* phase may be described as an intergrowth of O2 and OP4 phases and is generated by TMO_2_ layer slippage in the P2 phase to form O-type Na^+^ layers interspersed with P-type layers, with little crystallographic order from layer to layer. The *Z* phase is stabilized by Mg^2+^ migration to the tetrahedral sites in the O-type layers, as identified in the *ab initio* TS searches, the barrier for which is affected by changes in the lattice parameters, the presence of Na^+^, and the presence of stacking faults. This is the first report of *Z* phase formation and evidence for Mg^2+^ migration in this material.

During charge, we also observed a general increase in the Na^+^ mobility along the high-voltage charge plateau—due to an increase in the *c* axis (*i.e.*, an increase in the Na^+^ layer slab width) as well as an increase in the number of Na^+^ vacancies—and a gradual change in the electronic structure of NMMO.

On discharge, NMMO undergoes single-phase Na^+^ insertion into the *Z* phase. As Na^+^ vacancies are filled, layer slippage from O- to P-type layers takes place, eventually forming a P2-like phase at the end of discharge. This P2-like phase retains a few O-type layers and has a lower Na^+^ mobility compared to the material on charge, which we attribute to the presence of residual Mg^2+^ in the Na^+^ layers, giving rise to additional disorder in the P2-type phase formed on discharge.

The distinct phases that form during charge and discharge result in the large voltage hysteresis observed and likely stem from the asymmetric energy profile associated with Mg^2+^ migration. The role of Mg^2+^ migration on both the Na^+^ ion mobility and the changes in electronic structure—and potentially anion redox—will be the focus of future studies.

## Supporting Information Available

Detailed experimental, additional pristine and *ex situ* XRD Rietveld refinements, TEM results, additional electrochemical results, Mn *K*-edge EXAFS fits, additional TS search results, additional ^23^Na NMR results, details about the ^23^Na NMR shift calculations, magnetic susceptibility results, Na^+^/vacancy ordering schemes for NMMO, and GITT results (PDF)
