# Supplementary material for: Structural Origins of Voltage Hysteresis in the Na-Ion Cathode P2–Na0.67[Mg0.28Mn0.72]O2: A Combined Spectroscopic and Density Functional Theory Study
Source: Chem Mater. 2021 Jun 21;33(13):4890–906. doi: 10.1021/acs.chemmater.1c00248 (PMC8280737; doi:10.1021/acs.chemmater.1c00248)
Supplement: Supplementary file 1 — cm1c00248_si_001.pdf [file cm1c00248_si_001.pdf]

# Supporting Information

# Structural Origins of Voltage Hysteresis in the Na-ion Cathode P2-$Na_{0.67}[Mg_{0.28}Mn_{0.72}]O_2$: a Combined Spectroscopic and Density Functional Theory Study

Euan N. Bassey,[1] Philip J. Reeves,[1] Michael A. Jones,[1] Jeongjae Lee,[1,2] Ieuan D. Seymour,[3] Giannantonio Cibin,[4] Clare P. Grey[1,*]

[1]Department of Chemistry, University of Cambridge, Lensfield Road, Cambridge, CB2 1EW, United Kingdom

[2]School of Earth and Environmental Sciences, Seoul National University, Seoul 08826, Korea

[3]Department of Materials, Imperial College London, South Kensington Campus, London, SW7 2AZ, United Kingdom

[4]Diamond Light Source, Harwell Science and Innovation Campus, Didcot, OX11 0DE, United Kingdom

* To whom correspondence should be addressed:

E-mail: cpg27@cam.ac.uk

## Contents

**1 Experimental**
**2 Additional Rietveld and Pawley Refinements**
**3 Transmission Electron Microscopy Results**
**4 Additional Electrochemical Results**
**5 *Operando* X-Ray Diffraction Patterns**
**6 Rietveld Refinements of *Ex Situ* X-Ray Diffraction Patterns**
**7 Mn *K*-edge EXAFS Fits and Fitted Parameters**
**8 Additional $^{23}$Na NMR Results**
**9 *Ab initio* $^{23}$Na NMR Shift Calculations**
**10 Additional Single-ended Transition State Searches for OP4- and O2-$Na_x[Mg_{0.28}Mn_{0.72}]O_2$**
**11 Magnetic Susceptibility Measurements**
**12 Representative $Na^+$/Vacancy Ordering Schemes for P2-$Na_x[Mg_{0.28}Mn_{0.72}]O_2$**
**13 Galvanostatic Intermittent Titration Technique (GITT) Results**
**14 References**

## List of Figures


1 Expanded plots of the Rietveld refinement to NMMO
2 Pawley fit to (2×2×1) supercell of NMMO
3 TEM Images of pristine NMMO
4 Galvanostatic cycling curves for NMMO
5 Laboratory *operando* X-ray diffraction patterns
6 Rietveld refinement of *ex situ* X-ray diffraction patterns of pristine NMMO
7 Rietveld refinement of *ex situ* X-ray diffraction patterns of NMMO (point C1)
8 Rietveld refinement of *ex situ* X-ray diffraction patterns of NMMO (point C2)
9 Rietveld refinement of *ex situ* X-ray diffraction patterns of NMMO (point C3)
10 Rietveld refinement of *ex situ* X-ray diffraction patterns of NMMO (point C4)
11 Rietveld refinement of *ex situ* X-ray diffraction patterns of NMMO (point C5)
12 Rietveld refinement of *ex situ* X-ray diffraction patterns of NMMO (point D1)
13 Rietveld refinement of *ex situ* X-ray diffraction patterns of NMMO (point D2)
14 Rietveld refinement of *ex situ* X-ray diffraction patterns of NMMO (point D3)
15 Rietveld refinement of *ex situ* X-ray diffraction patterns of NMMO (point D4)
16 Rietveld refinement of *ex situ* X-ray diffraction pattern of NMMO with and without migrated $Mg^{2+}$, and with migrated octahedral $Mg^{2+}$ (point C5)
17 Fit to Mn *K*-edge EXAFS data of pristine NMMO (point C0)
18 Fit to Mn *K*-edge EXAFS data of pristine NMMO (point C2)
19 Fit to Mn *K*-edge EXAFS data of pristine NMMO (point C5)
20 Fit to Mn *K*-edge EXAFS data of pristine NMMO (point D3)
21 Fits to Mn *K*-edge EXAFS data of NMMO at point C5, with and without migrated $Mg^{2+}$, and with migrated octahedral $Mg^{2+}$
22 Two-dimensional $^{23}$Na NMR pjMATPASS spectrum for pristine NMMO
23 One-dimensional $^{23}$Na NMR pjMATPASS isotropic slice spectra for pristine NMMO at different states of charge
24 Comparisons of $^{23}$Na NMR Hahn-echo spectra acquired under 40 kHz and 60 kHz MAS rates for *ex situ* samples of NMMO at points C1, C5 and D1
25 Variable-temperature $^{23}$Na NMR spectra for pristine NMMO
26 Variable-temperature $^{23}$Na NMR spectra for *ex situ* NMMO at point C1
27 Variable-temperature NMR temperature calibration curve
28 Paramagnetic $^{23}$Na NMR shift bond pathways in P2-NMMO
29 Simulated non-exchanging $^{23}$Na NMR spectrum for P2-NMMO with a random distribution of *TM* cations
30 Simulated non-exchanging $^{23}$Na NMR spectrum for P2-NMMO with a honeycomb-ordered distribution of *TM* cations
31 $Mg^{2+}$ migration energy profiles for O2- NMMO (variable cell sizes)
32 $Mg^{2+}$ migration energy profiles for OP4- NMMO (variable cell sizes)
33 Variation in $Mg^{2+}$ migration energy barriers with distance to nearby $Na^+$ for O2-NMMO
34 $Mg^{2+}$ migration energy profiles for OP4- and O2-NMMO with stacking faults
35 Energy profile for $Mg^{2+}$ hopping between O-type tetrahedral and octahedral sites in O2-NMMO
36 Zero-field cooled and field-cooled magnetic susceptibility data for pristine NMMO
37 $Na^+$/vacancy ordering schemes in P2-NMMO
38 GITT data for NMMO during the first charge
39 GITT data for NMMO during the first discharge

## List of Tables


1 Open circuit voltages of *ex situ* half-cells after rest period
2 Fitted parameters from Mn *K*-edge EXAFS data
3 Longitudinal and transverse relaxation times ($T_1$ and $T_2$) for *ex situ* samples of NMMO
4 Calculated quadrupole-induced $^{23}$Na shifts for P2-$Na_{2/3}[Mg_{1/3}Mn_{2/3}]O_2$
5 Bond pathway-derived $^{23}$Na NMR hyperfine shifts of sites in P2-$Na_{2/3}[Mg_{1/3}Mn_{2/3}]O_2$ (with a random $Mg^{2+}$/$Mn^{4+}$ distribution) whose shifts are closest to the observed spectrum
6 Bond pathway-derived $^{23}$Na NMR hyperfine shifts of sites in honeycomb-ordered P2-$Na_{2/3}[Mg_{1/3}Mn_{2/3}]O_2$
7 Bulk magnetic susceptibility data, extracted from the Curie-Weiss law fit to the Zero-Field Cooled susceptibility data

# 1. Experimental

## Synthesis.

Stoichiometric quantities of $Na_2CO_3$ (Sigma-Aldrich, 99%), $Mn_2O_3$ (Aldrich, 99%) and MgO (Acros Organics, 99.99%) were weighed inside an Ar-filled glovebox, removed from the glovebox and ball-milled (Retsch PM 100 planetary ball mill; a zirconia jar with approximately one hundred 3 mm diameter zirconia balls was used whilst milling at 400 rpm) for two hours—consisting of 15-minute milling periods interspersed with 15-minute rests. Batches (approximately 1 – 2 g) of this mixture were heated, in an alumina crucible, at a rate of 10 K $min^{-1}$ to 1073 K under flowing $O_2$ gas (flow rate approximately 30 mL $min^{-1}$) and maintained at 1073 K for 10 hours. The powder cooled naturally in the tube furnace for eight hours and was transferred to an Ar-filled glovebox within one minute of removal from the furnace. The black powder was ground by hand in an agate pestle and mortar, removed from the glovebox and returned to the tube furnace under flowing Ar gas (approximately 30 mL $min^{-1}$). The powder was heated to 973 K at a rate of 10 K $min^{-1}$ under Ar flow and then immediately quenched by bodily shifting the tube so that the crucible was no longer inside the furnace but remained under Ar flow. Once the furnace had cooled to room temperature, the powder was transferred to an Ar-filled glovebox and the powder ground by hand using an agate pestle and mortar. $Na_{0.67}[Mg_{0.28}Mn_{0.72}]O_2$ was collected as a dark brown microcrystalline powder.

## XRD.

In all cases, laboratory powder X-ray diffraction patterns were recorded using a PANalytical Empyrean diffractometer (Cu Kα radiation, $\lambda$ = 1.541 Å). For the pristine material, a powder sample was sealed in a borosilicate glass capillary tube (outer diameter 0.5 mm) using two-component glue and then fourteen diffraction patterns over the range $2\theta$ = 5 – 90° (step size 0.02° and scanning speed 0.02° $s^{-1}$) were recorded and summed. The resulting pattern is the sum of the fourteen patterns obtained.

The *ex situ* cathode film samples were placed between two sheets of polyimide (Kapton) film inside an Ar-filled glovebox and sealed with both grease and the external sample holder. This arrangement was verified to prevent air exposure over at least a two-hour period, with no contamination of the sample from the grease (diffraction patterns were recorded within 10 minutes of removing the sample inside the sample holder from the glovebox). Diffraction patterns for the *ex situ* samples were recorded over the range $2\theta$ = 5 – 80°, with a step size 0.02° and scanning speed 0.02° $s^{-1}$.

The analysis of all diffraction data was carried out using the TOPAS Academic 6 structure refinement software package.[1,2]

## Electrochemical Characterisation.

All electrochemical measurements were conducted using stainless-steel coin cells. The cathodes used had an active material:carbon:binder mass ratio of 8:1:1, with an active material loading of 1.5 – 9.5 mg $cm^{-2}$. The active material and conductive carbon (Super P, TIMCAL) were mixed in an agate pestle and mortar in an Ar-filled glovebox. To this, polyvinylidene fluoride (PVDF, Kynar) dissolved in *N*-methyl-2-pyrrolidone (NMP; 0.3 – 0.7 $cm^3$; Sigma-Aldrich, anhydrous, 99.5%) was added and the slurry stirred until no observable solid particles remained. The resulting slurry was poured onto Al foil mounted onto a glass plate and cast

using a 500 μm doctor blade. The cast films were left to dry for at least two hours at ambient temperature inside the glovebox and then dried overnight under dynamic vacuum. Cathode discs (13 mm diameter) were punched out inside the glovebox and then heated at 120°C under dynamic vacuum for 12 hours.

The cathode discs were assembled into half-cells against Na metal foil anodes, prepared from cubes of Na metal (Sigma-Aldrich, in oil) by cutting, pressing and cleaning the cube in hexane (Sigma-Aldrich, mixture of isomers, anhydrous, 99%) or *n*-heptane (Fischer Scientific, 99+%, anhydrous). Once pressed into a thin sheet, Na metal discs were punched out by hand (13 mm diameter). The electrolyte for all experiments was 1.0 M $NaPF_6$ (Acros Organics, 98.5+%; dried at 120°C for 12 hrs under dynamic vacuum) dissolved in propylene carbonate (PC; Solvionic, anhydrous). No additives were used. The electrolyte solution was soaked into a glass microfibre separator (Whatman, Grade GF/B, 16 mm diameter). A galvanostat/potentiostat (BioLogic) with EC laboratory software was used to perform electrochemical experiments for electrochemical assessment, *ex situ* NMR and *ex situ* XRD. For *ex situ* Mn K-edge EXAFS samples, electrochemical experiments were carried out using an Arbin galvanostat/potentiostat. Unless stated otherwise, all half cells of $Na_{0.67}[Mg_{0.28}Mn_{0.72}]O_2$ against Na metal were cycled at a rate of 10 mA $g^{-1}$ (corresponding to approximately *C*/19, for a theoretical *C* rate relative to the pristine material and assuming complete desodiation of the pristine material).

Galvanostatic intermittent titration technique (GITT) experiments were also performed on half-cells of $Na_{0.67}[Mg_{0.28}Mn_{0.72}]O_2$ against Na metal at a temperature of 20(2)°C. Current pulses whose magnitudes correspond to a rate of 10 mA $g^{-1}$ were applied for 10 minutes, followed by a 6 hr rest period. The diffusion coefficient of $Na^+$, *D*, was determined using the approach detailed by Verma *et al.*,[3] using the equation:

$$D = \frac{4}{\pi}\left(\frac{IV_\mathrm{m}}{FS}\right)^2\left(\frac{\mathrm{d}U_0/\mathrm{d}y}{\mathrm{d}V/\mathrm{d}\sqrt{t}}\right)^2,$$

where $I$ is the applied current during the current pulses, $V_\mathrm{m}$ the molar volume of NMMO, $F$ Faraday's constant, $S$ the active area of the electrode, $U_0$ the open-circuit voltage (OCV) of the electrode at the end of each rest period, $y$ the $Na^+$ content of NMMO ($Na_{1-y}[Mg_{0.28}Mn_{0.72}]O_2$), $V$ the voltage during the pulse periods and $t$ the time during the pulse periods. This equation assumes $t \ll \frac{R^2}{D}$, where $R$ is the diffusion length.

Since the molar volume and active area of the electrode are difficult to accurately determine during the experiment (as both depend on the sizes of particles and pores in the electrodes, and not all of the electrode contains active material), in this work, it is assumed that $V_\mathrm{m}$ and $S$ remain constant throughout the experiment, and the relative changes in $Na^+$ mobility are instead determined using:[3]

$$D\left(\frac{S}{V_\mathrm{m}}\right)^2 = \frac{4}{\pi}\left(\frac{I}{F}\right)^2\left(\frac{\mathrm{d}U_0/\mathrm{d}y}{\mathrm{d}V/\mathrm{d}\sqrt{t}}\right)^2.$$

To determine $\mathrm{d}U_0/\mathrm{d}y$, the variation of $U_0$ with $y$ was fitted to a polynomial function which was then differentiated to obtain $\mathrm{d}U_0/\mathrm{d}y$ as a function of $y$. The values of $\mathrm{d}V/\mathrm{d}\sqrt{t}$ were determined from the slope of the linear function of $V$ *vs* $\sqrt{t}$.

## Operando XRD.

*Operando* X-ray diffraction measurements were carried out using a PANalytical Empyrean diffractometer (Cu Kα radiation, $\lambda$ = 1.541 Å). The diffraction patterns were recorded at ambient temperature in Bragg-Brentano geometry. The electrolyte and Na metal foil anode were prepared as described above; the electrolyte was soaked into a glass microfibre separator (19 mm diameter, Whatman, Grade GF/B). The cathode was prepared as above, but the Al foil current collector was peeled off before placing the cathode in the cell. The cell was left to rest for at least one hour before beginning measurements. Diffraction patterns were recorded over the range $2\theta$ = 5 – 50°, with a step size 0.02° and scanning speed 0.02° $s^{-1}$.

## Transmission Electron Microscopy (TEM).

A powder sample of pristine NMMO was mounted onto a lacey carbon-coated Cu grid and loaded into a double-tilt vacuum transfer holder (648, Gatan Inc.). TEM images were taken with a Thermo Scientific (FEI) Talos F200X G2 instrument. The microscope was operated at 200 kV whilst acquiring images; 40 μm and 10 μm apertures were used to acquire the selected area electron diffraction patterns.

## Variable-Temperature $^{23}$Na NMR.

Electrochemically cycled cathodes of NMMO were prepared by cycling a cathode to a given cutoff voltage and allowing the cell to rest for at least one hour. The cell was opened inside an Ar-filled glovebox and the cathode extracted, washed in dimethyl carbonate (DMC; approximately 1 $cm^3$; Sigma-Aldrich, 99%, anhydrous) and dried *in vacuo* for at least 20 minutes. The cathode was then scraped off the Al foil current collector and packed into a 2.5 mm diameter $ZrO_2$ MAS rotor, using polytetrafluoroethylene (PTFE) tape to fill the gap at either end of the rotor. No rotor spent longer than 10 minutes outside of the glovebox before being inserted into the magnet under a protective atmosphere of flushing nitrogen gas.

The variable temperature $^{23}$Na NMR spectra were recorded on a Bruker Avance III 11.7 T spectrometer using a Bruker 2.5 mm magic angle spinning (MAS) probe with an MAS frequency of 28 kHz and an effective $\frac{\pi}{2}$ pulse length of 1.15 μs (this corresponds to $\frac{\pi}{6}$, which is longer than the selective $\frac{\pi}{8}$ pulse to ensure all quadrupolar $^{23}$Na centres are in the quadrupolar liquid limit;[4] we therefore selected a compromise between the linear quadrupolar regime and maximising the signal intensity). The 2.5 mm $ZrO_2$ MAS rotor was chosen so that a relatively wide range of temperatures could be accessed; the spinning speed chosen was 28 kHz, as this was sufficient to separate the spinning sideband manifold from its isotropic resonance. A rotor-synchronised Hahn-echo pulse sequence (90°–$\tau$–180°–$\tau$–acquire) was used for quantitative measurements and the recycle delay (25 ms; at least $5T_1$) was set such that the bulk, paramagnetically shifted signal was recorded quantitatively, while the diamagnetic signal due to electrolyte decomposition products was suppressed. All spectra were referenced to solid NaCl at 7.21 ppm and scaled according to the mass of the sample and number of residuals recorded.

Temperature calibration measurements were performed *ex situ* using the $^{207}$Pb shift of $Pb(NO_3)_2$ (Alfa Aesar, 99%) under the same heater powers, nitrogen gas flow rates, and driving and bearing pressures used for $^{23}$Na NMR experiments [Figure S27].

## First-Principles Calculations of the $^{23}$Na Hyperfine and Quadrupolar Shifts.

The shifts of each Na site were calculated using methods described previously.[5–8] An initial geometry optimisation was performed using the VASP code,[9–11] employing the projector-augmented wave (PAW) method.[12,13] These calculations used the spin-polarised Perdew-Burke-Ernzerhof (PBE) exchange-correlation functionals, applying the Hubbard *U* model[14,15] within the rotationally invariant formalism proposed by Liechstenstein *et al.*,[16] to correct for known deficiencies of pure functionals for highly localised 3*d* states.[17] The plane-wave energy cutoff was set to 520 eV and an effective Hubbard *U* parameter for Mn, $U_{\mathrm{eff}} = U - J = 3.9$ eV, where *U* and *J* are the effective on-site Coulomb and exchange parameters ($J$ = 1 eV), respectively, was chosen in line with previous work on the parent material, $Na_xMnO_2$.[18] SCF cycles were converged with an energy tolerance of $10^{-5}$ eV. Monkhorst-Pack[19] *k*-point sampling about the gamma point of < 0.5 Å was used in the Brillouin zone.

Periodic spin-polarised DFT calculations were performed in CRYSTAL.[20] Hyperfine parameters were calculated with B3LYP[21,22] hybrid functionals containing 20% and 35% Hartree-Fock exchange, referred to as Hyb20 and Hyb35, respectively. These weights were chosen based on the success of these functionals in calculating the properties of *TM* compounds and have been previously reported to provide an upper and lower bound on experimental shifts.[5–7]

The calculations employed two basis sets: a smaller basis set for geometry optimisations (denoted BS-I) and a more extended set for the single-point hyperfine calculations (BS-II). The BS-I sets were taken—without modification—from solid-state studies by Catti *et al.*,[23–26] whilst the BS-II sets comprised bases from the Ahlrichs set for metal ions[27] and IGLO-III basis set for O.[28] The BS-I sets are of the form (15*s*7*p*)/[1*s*3*sp*] for Na, (15*s*3*p*)/[1*s*3*sp*] for Mg, (20*s*12*p*5*d*)/[1*s*4*sp*2*d*] for Mn and (14*s*6*p*)/[1*s*3*sp*] for O. The BS-II sets are of the form (11*s*7*p*)/[7*s*3*p*] for Na, (11*s*7*p*)/[6*s*3*p*] for Mg, (13*s*10*p*5*d*)/[7*s*6*p*3*d*] for Mn and (10*s*6*p*2*d*)/[6*s*5*p*2*d*] for O. In both cases, the values in parentheses denote the number of Gaussian primitives and the values in square brackets denote the contraction scheme.

An initial geometry optimisation on a spin-locked ferromagnetic configuration was run using the BS-I set, with each cell well-converged with an energy tolerance of $10^{-5}$ AU, a root-mean-square force gradient tolerance of $3 \times 10^{-4}$ AU and integral thresholds of $10^{-8}$, $10^{-8}$, $10^{-8}$, $10^{-8}$ and $10^{-16}$ for the Coulomb overlap, Coulomb penetration, exchange overlap and *g* and *n* series exchange penetration. A subsequent geometry optimisation in which the spins were unconstrained was run, using the spin-locked wavefunction and structure as initial guesses and the same energy tolerances and integral thresholds as the spin-locked calculation. This spin-relaxed geometry-optimised structure was then used in subsequent single-point hyperfine calculations, run using the BS-II basis sets.

A ferromagnetic spin-locked single-point calculation was then carried out; the total energy of each cell was well-converged with a tolerance of $10^{-7}$ AU and integral thresholds of $10^{-7}$, $10^{-7}$, $10^{-7}$, $10^{-7}$ and $10^{-14}$ for the Coulomb overlap, Coulomb penetration, exchange overlap and *g* and *n* series exchange penetration. Finally, another single-point hyperfine calculation was run, starting from the converged spin-locked wavefunction, but this time allowing the spins to relax. The energy tolerances and integral thresholds were the same as in the spin-locked case.

To determine the bond pathways, $Mn^{4+}$ spins were flipped and a single-point hyperfine calculation was then run with the total magnetisation of the cell fixed to give a ferromagnetic

state for the first ten SCF cycles, after which no spin constraints were imposed. These runs generated well-converged spin states, again using the same tolerances as before.

### Magnetic Property Measurements.

Measurements of the magnetic susceptibility were carried out on a powder sample of pristine NMMO (mass 13.2 mg), using a Quantum Design Magnetic Property Measurement System 3 (MPMS) superconducting quantum interference device (SQUID) magnetometer. The zero-field cooled (ZFC) and field cooled (FC) susceptibilities were measured in an applied field of 0.01 T over the temperature range 2 – 300 K. The small field approximation for the susceptibility $\chi(T) \simeq \frac{M}{H}$, where *M* is the magnetisation and *H* is the magnetic field intensity, was assumed valid. Data were corrected for diamagnetism of the sample using Pascal's constants.[29]

## 2. Additional Rietveld and Pawley Refinements

The Rietveld refinement to the laboratory XRD pattern obtained for pristine $Na_{0.67}[Mg_{0.28}Mn_{0.72}]O_2$ is shown in Figure S1(a). Overall, the fit to the data is adequate, but there are some peaks which could not be fit and several peaks whose intensity is poorly matched. The additional peaks can be assigned to a (2×2×1) superstructure, with the reflections at $Q$ = 1.90 Å$^{-1}$, 1.98 Å$^{-1}$, 2.60 Å$^{-1}$ and 3.11 Å$^{-1}$ corresponding to $\left(\frac{3}{2}\frac{\bar{1}}{2}\mathbf{0}\right)$, $\left(\frac{3}{2}\frac{\bar{1}}{2}\mathbf{1}\right)$, $\left(\frac{3}{2}\frac{\bar{1}}{2}\mathbf{2}\right)$ and $\left(\frac{3}{2}\mathbf{02}\right)$ (relative to the unit cell, rather than the supercell), respectively.

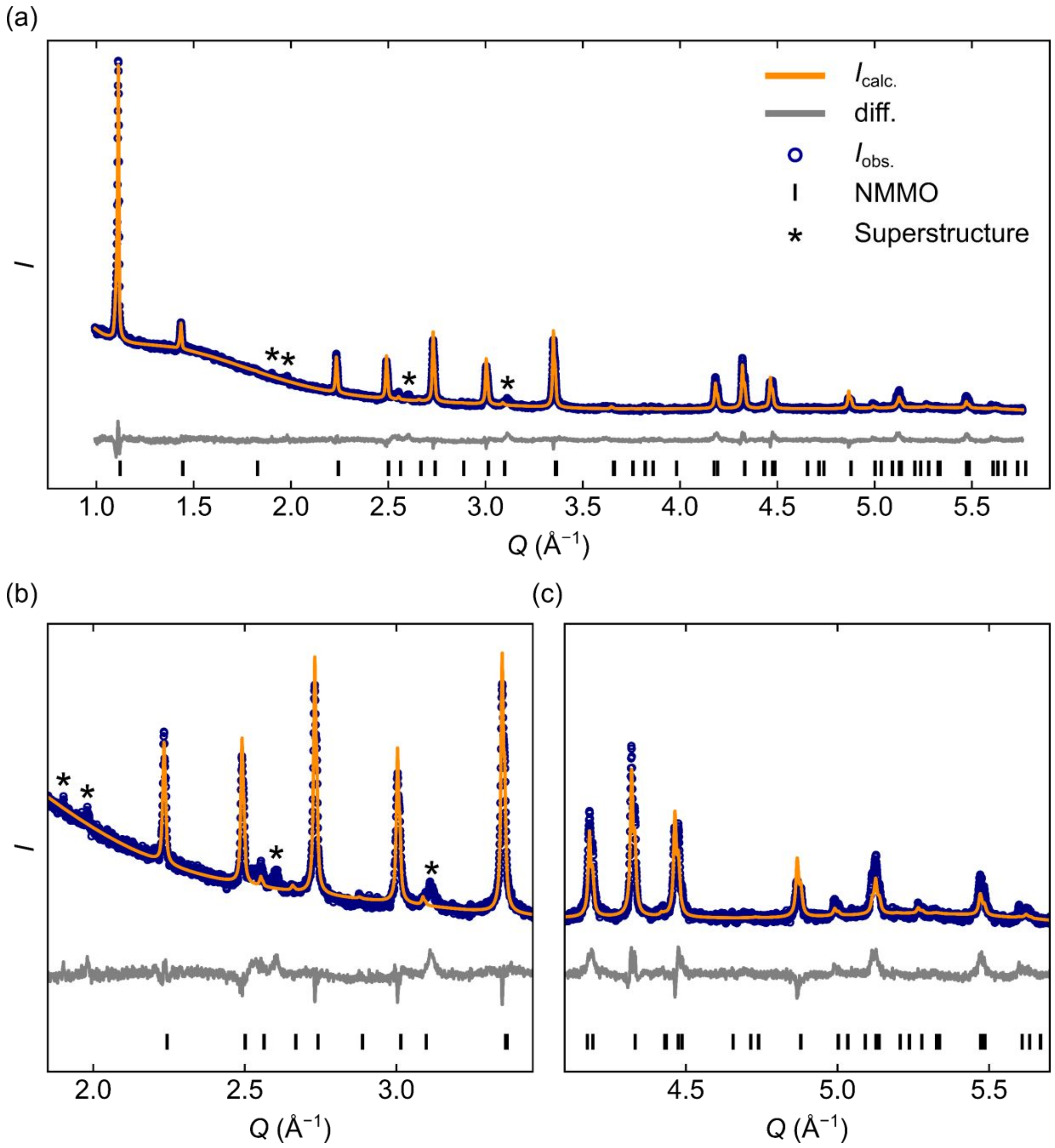


**Figure S1:** Rietveld refinement of the laboratory PXRD pattern collected for pristine NMMO at room temperature ($R_{wp}$ = 4.47%). **(a)** shows the refinement for the whole pattern, whilst **(b)** and **(c)** show expansions of the fit over the regions $Q$ = 1.85 Å$^{-1}$ to 3.45 Å$^{-1}$ and $Q$ = 4.10 Å$^{-1}$ to 5.70 Å$^{-1}$, respectively.

To identify the superstructural ordering in pristine NMMO, we carried out a Pawley refinement to a (2×2×1) supercell [Figure S2]. This accurately models the diffraction pattern observed, indicating that this is the lengthscale of ordering seen in NMMO. Additional Rietveld refinements to (2×2×1) supercells with a long zig-zag ordering of $Na^+$ ions and vacancies did not significantly improve the fit compared to the unit cell Rietveld [Figure 2 and S1]. The exact nature of ordering in NMMO is beyond the scope of this work but will be the topic of a future study.

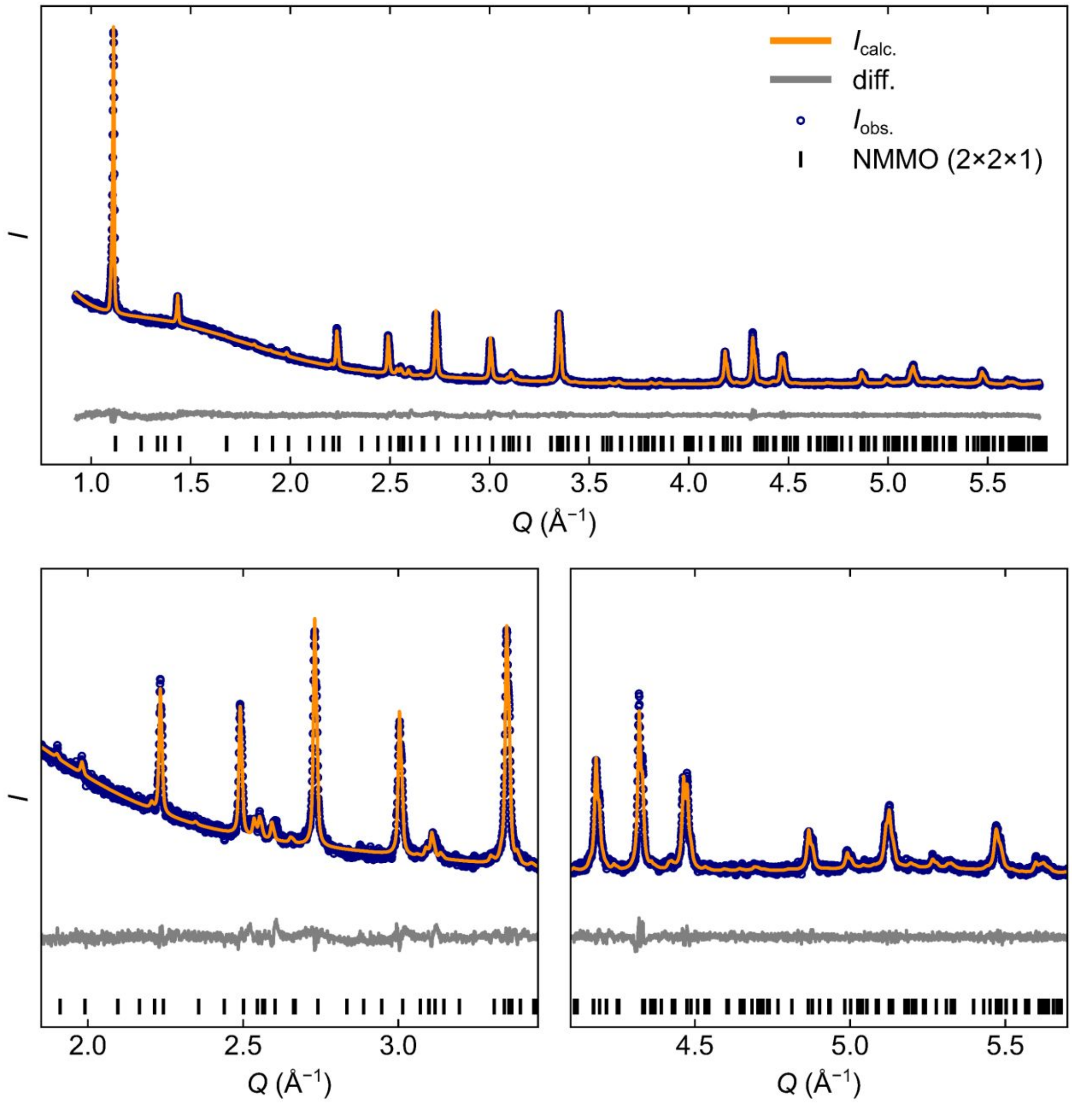


**Figure S2:** Pawley refinement of the laboratory PXRD pattern collected for pristine NMMO at room temperature ($R_{wp}$ = 2.97%). **(a)** shows the refinement for the whole pattern, whilst **(b)** and **(c)** show expansions of the fit over the regions $Q$ = 1.85 Å$^{-1}$ to 3.45 Å$^{-1}$ and $Q$ = 4.10 Å$^{-1}$ to 5.70 Å$^{-1}$, respectively.

## 3. Transmission Electron Microscopy Results

To further examine the elemental distribution in NMMO, we carried out TEM, which revealed an even distribution of Na, Mg and Mn across the particles [Figure S3(c) to (g)] and confirmed a hexagonal arrangement of Mg and Mn ions, with little contrast between the observed atoms [Figure S3(a) and (b)], consistent with the “random” Mg/Mn distribution across the *TM* sites seen in PXRD. The selected area electron diffraction (SAED) pattern comprised a set of intense spots, which were indexed to a hexagonal lattice, as well as a set of less intense spots [Figure S3(h)], which were indexed to superlattice reflections, likely due to (partial) honeycomb order across the *TM* sublattice. Additional weak spots were observed but could not be indexed; these likely arise from material with a subtly different orientation underneath the beam.

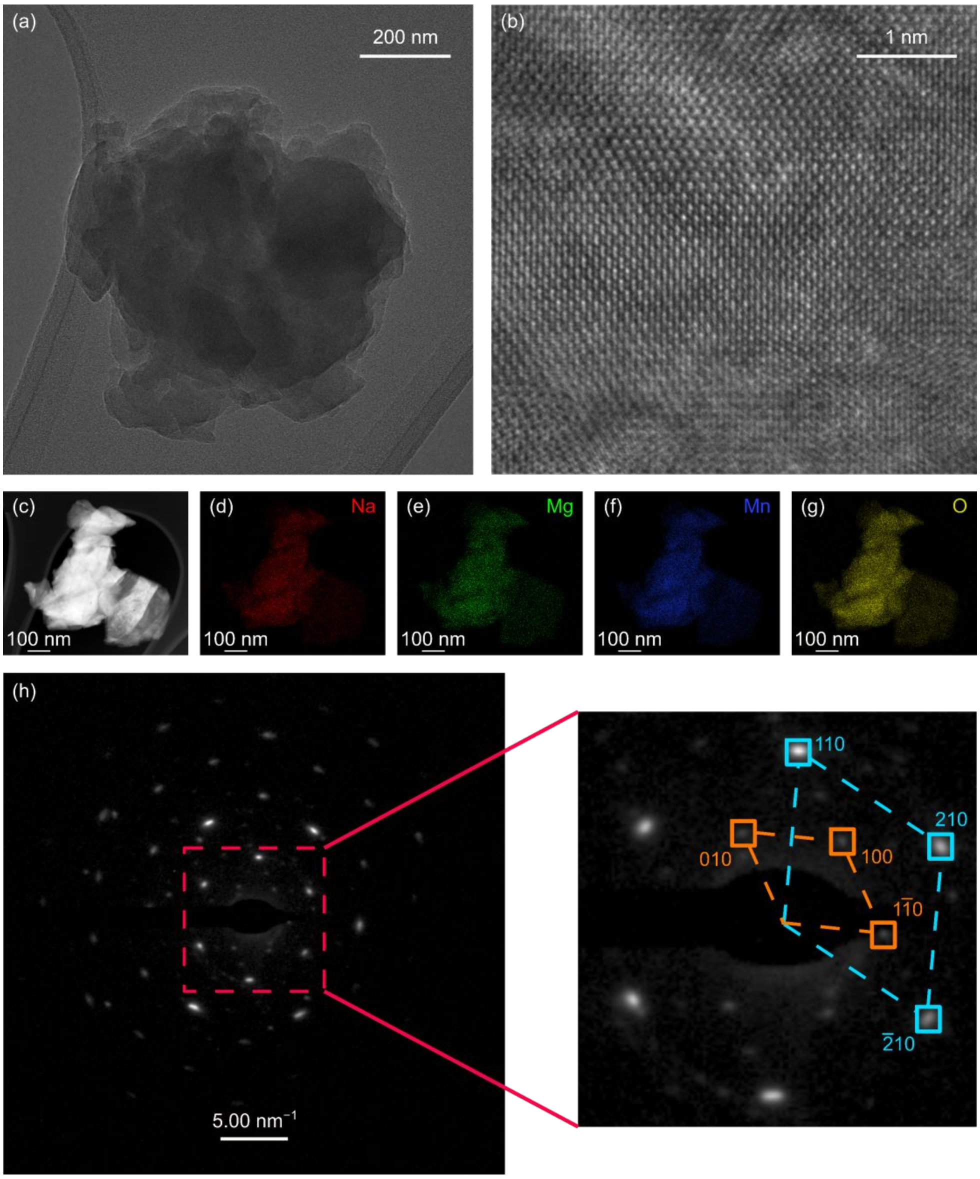


**Figure S3:** TEM images of pristine NMMO. **(a)** shows a plate-like particle, whilst **(b)** is an image of the same particle at a higher magnification. **(c)**, **(d)**, **(e)**, **(f)** and **(g)** show the high-angle annular dark field images with *Z*-contrast to reveal the Na, Mg, Mn and O distributions. **(h)** shows the selected area electron diffraction pattern of the particle in **(a)** and **(b)**, alongside an expansion to highlight the hexagonal diffraction pattern.

# 4. Additional Electrochemical Results

All *ex situ* half-cells of $Na_{0.67}[Mg_{0.28}Mn_{0.72}]O_2$ (henceforth NMMO) were allowed to rest for at least one hour after cycling to ensure that the electrochemically-generated thermodynamic products formed. The open circuit voltages (OCVs) of the cells at each stage of charge are given in Table S1.

**Table S1:** Open circuit voltages (OCVs) of cells cycled to a given cutoff voltage, after at least one hour's rest; standard errors are shown in parentheses.

| Sample label | Cutoff | OCV (V) |
|---|---|---|
| C0 | - | - |
| C1 | 2.90 V (1$^{st}$ Charge) | 2.855(2) |
| C2 | 4.22 V (1$^{st}$ Charge) | 3.830(2) |
| C3 | 4.247 V (1$^{st}$ Charge) | 4.067(2) |
| C4 | 4.265 V (1$^{st}$ Charge) | 4.118(2) |
| C5 | 4.50 V (1$^{st}$ Charge) | 3.983(2) |
| D1 | 3.50 V (1$^{st}$ Discharge) | 3.774(2) |
| D2 | 2.75 V (1$^{st}$ Discharge) | 2.972(2) |
| D3 | 2.142 V (1$^{st}$ Discharge) | 2.264(2) |
| D4 | 1.50 V (1$^{st}$ Discharge) | 1.867(2) |

The first charge-discharge cycle for NMMO displays a large voltage hysteresis; on subsequent cycles, the hysteresis becomes smaller and the voltage profile becomes more sloping [Figure S4].

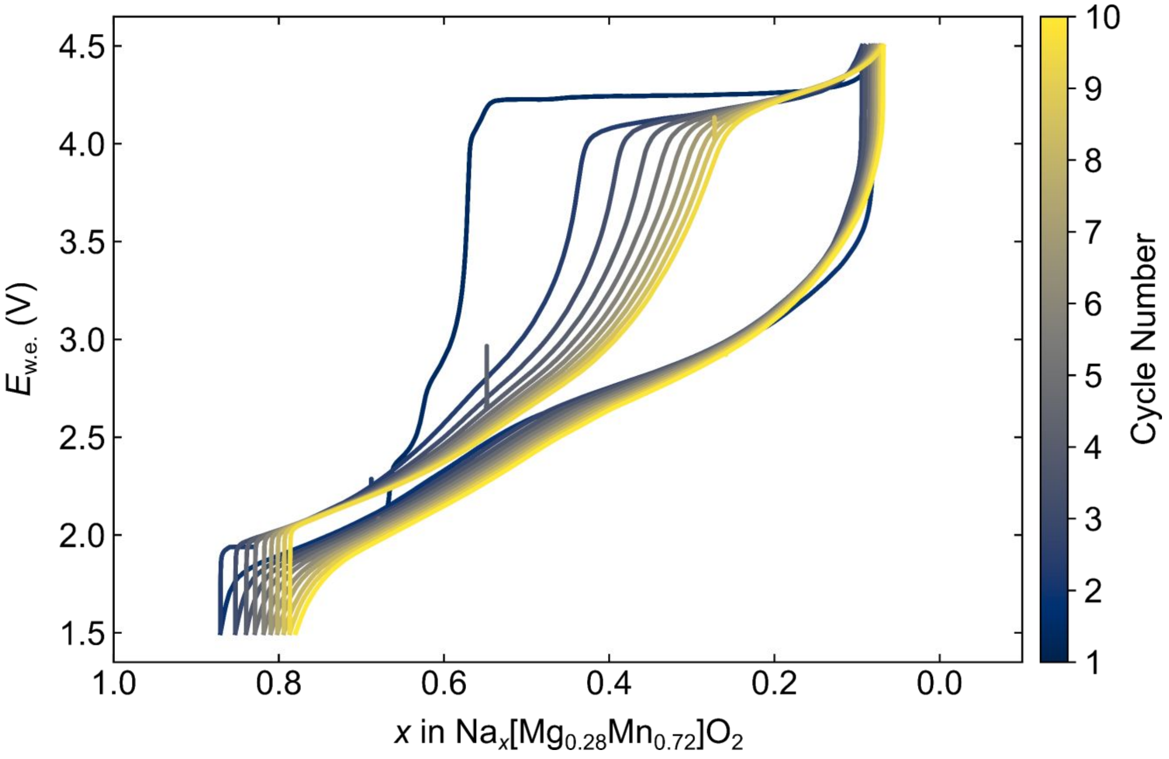


**Figure S4:** Galvanostatic cycling curves for $Na_{0.67}[Mg_{0.28}Mn_{0.72}]O_2$, cycled at a rate of 10 mA $g^{-1}$.

## 5. *Operando* X-Ray Diffraction Results

The bulk phase changes in NMMO were monitored using both *operando* and *ex situ* X-ray diffraction (XRD). For the *operando* measurements, NMMO was cycled at a rate of 10 mA $g^{-1}$, with diffraction patterns recorded continuously every 10 minutes (corresponding to a change in the Na content of $\Delta x$ = 0.006 during acquisition of each diffraction pattern). To avoid swamping the observed diffraction pattern for NMMO with diffraction from Be (a component of the *operando* cell), the diffraction pattern was recorded over the range $2\theta$ = 5 – 50° [Figure S5]. The patterns recorded have too low a resolution to accurately extract the variation in lattice parameters *via* Rietveld refinement, but nevertheless provide a good qualitative observation of the bulk phase evolution in NMMO. The low-*Q* region of the diffraction patterns [Figure S5(c)] show the change in the position of the (002) reflection for the P2 and O2 phases, as well as the (004) reflection of the OP4 phase; the reflections in this region act as useful indicators of the appearance and disappearance of each phase during charge and discharge. Figure S5(d) shows the variation in the (100) reflection of P2-NMMO, with the loss in intensity after charge and discharge indicating a decrease in Mg/Mn ordering, compared to the pristine material.

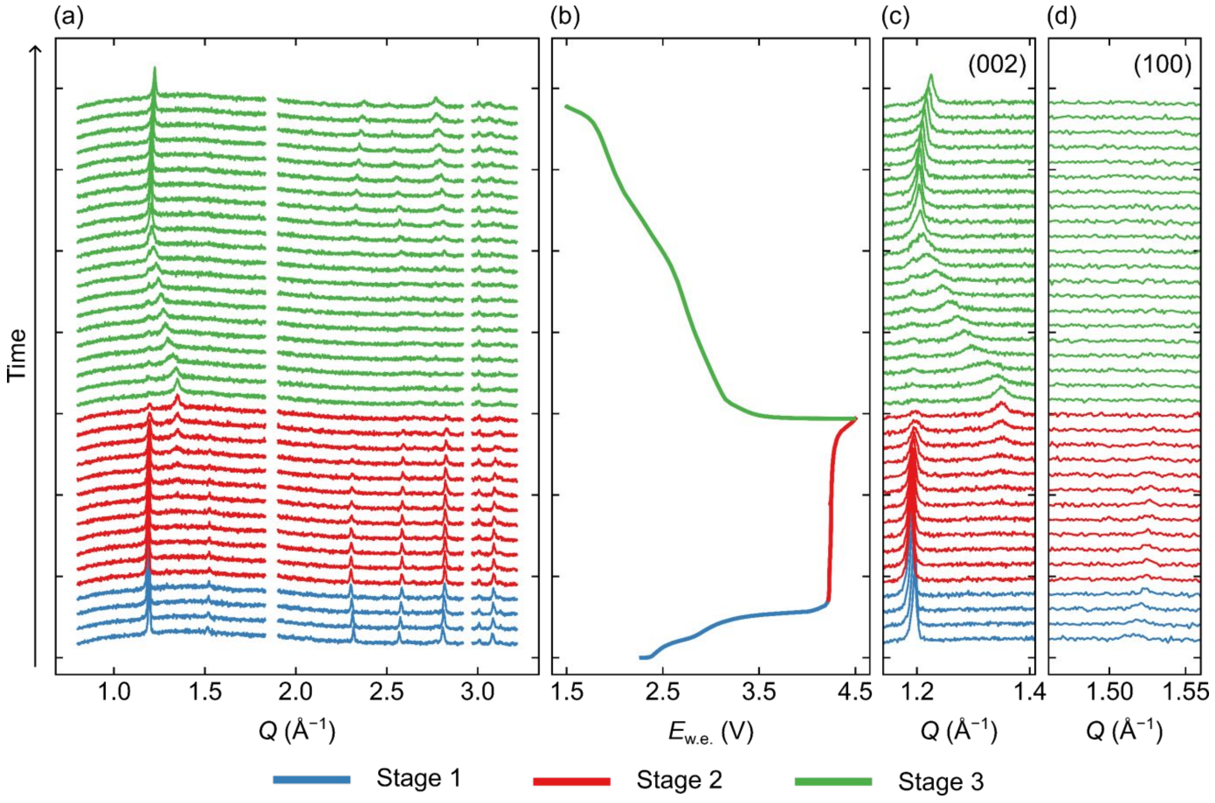


**Figure S5: (a)** *Operando* XRD patterns collected for NMMO cycling at a rate of 10 mA $g^{-1}$, with corresponding voltage profile shown in **(b)**. Expansions of the P2 phase (002) and (100) reflections are highlighted in **(c)** and **(d)**, respectively. Note that the height of the diffraction pattern is chosen so that it aligns with the start time of acquisition.

## 6. Rietveld Refinements of *Ex Situ* X-Ray Diffraction Patterns

To monitor the change in lattice parameters during charge and discharge, Rietveld refinements of the *ex situ* XRD patterns were performed in TOPAS Academic 6.0.[2,30,31] The fits to the diffraction patterns are shown in Figures S6 to S15.

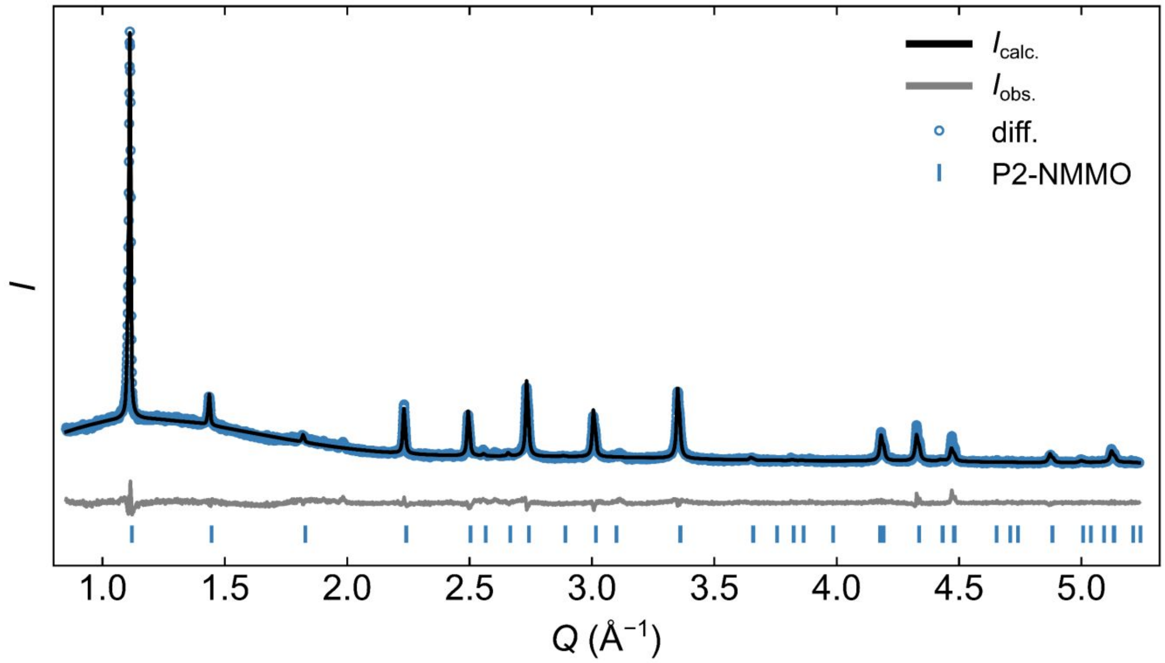


**Figure S6:** Rietveld refinement of the laboratory XRD pattern collected for a pristine cathode film (point C0) of NMMO at room temperature ($R_{wp}$ = 6.0%).

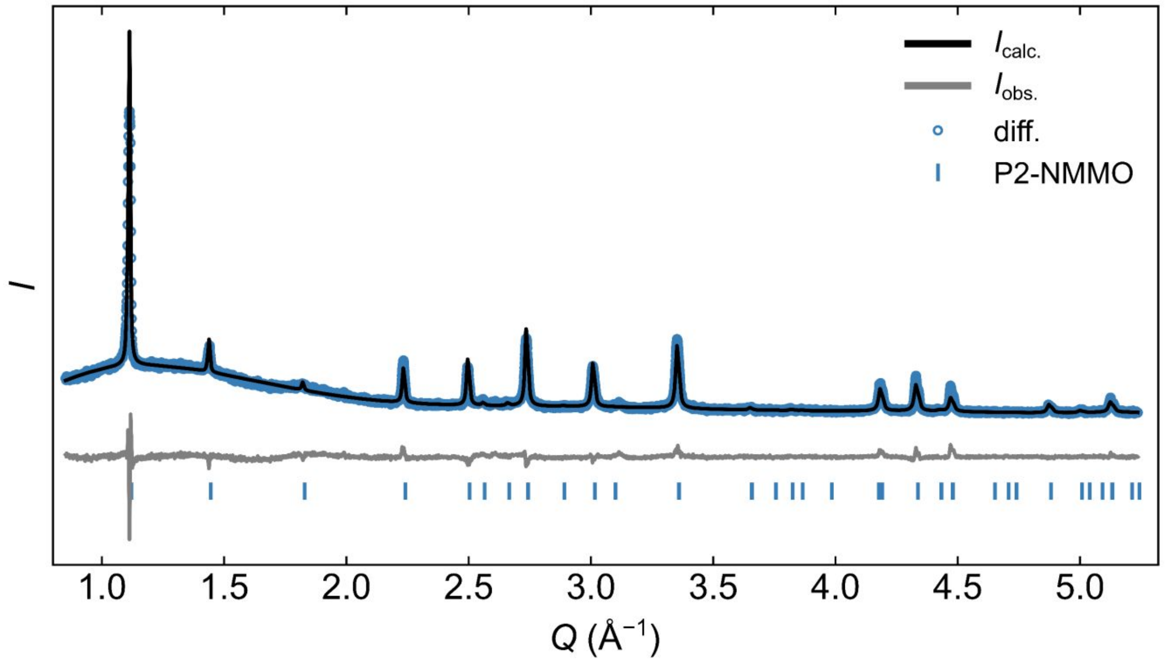


**Figure S7:** Rietveld refinement of the laboratory XRD pattern collected for an *ex situ* cathode of NMMO at point C1, recorded at room temperature ($R_{wp}$ = 6.8%).

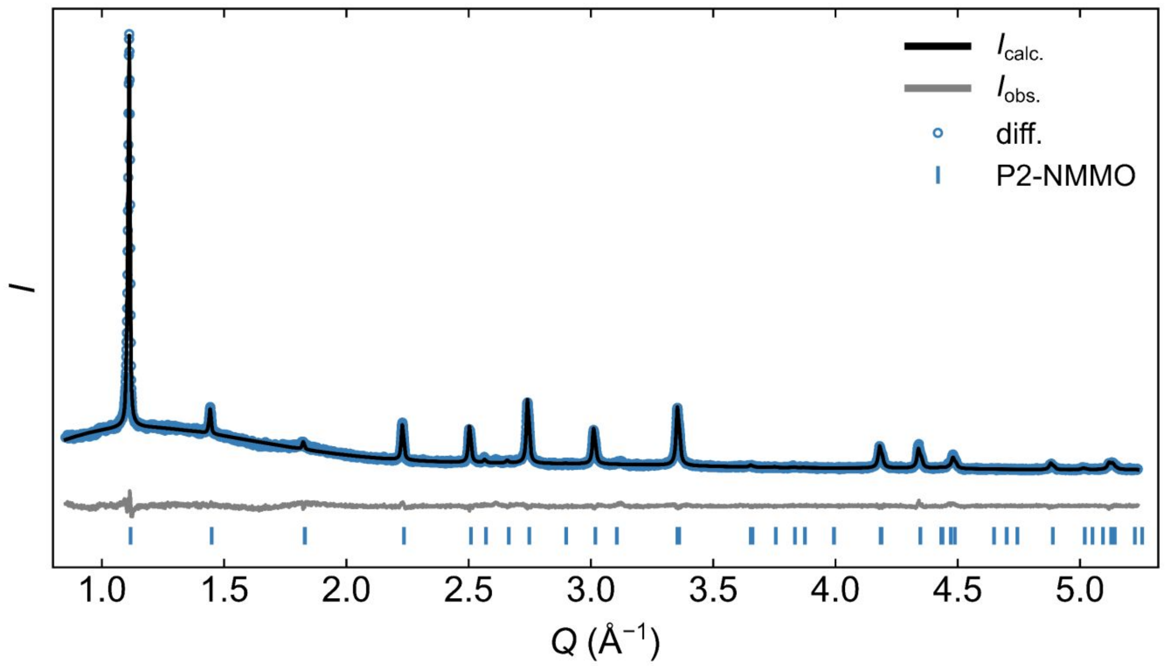


**Figure S8:** Rietveld refinement of the laboratory XRD pattern collected for an *ex situ* cathode of NMMO at point C2, recorded at room temperature ($R_{wp}$ = 5.3%).

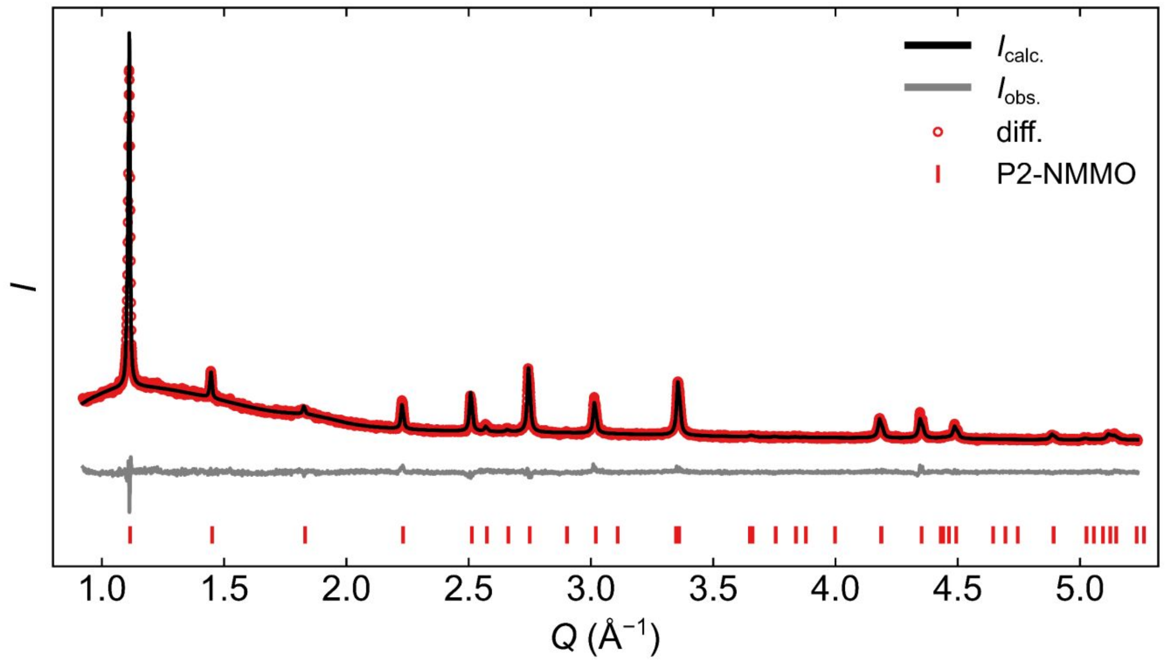


**Figure S9:** Rietveld refinement of the laboratory XRD pattern collected for an *ex situ* cathode of NMMO at point C3, recorded at room temperature ($R_{wp}$ = 4.7%).

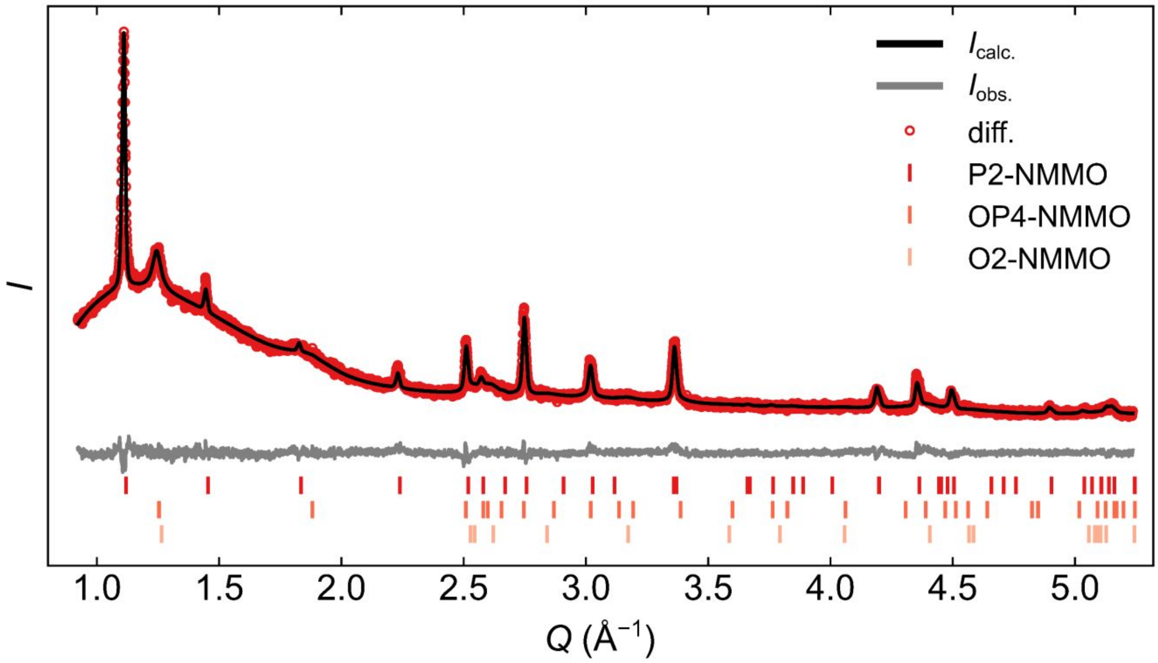


**Figure S10:** Rietveld refinement of the laboratory XRD pattern collected for an *ex situ* cathode of NMMO at point C4, recorded at room temperature ($R_{wp}$ = 4.2%).

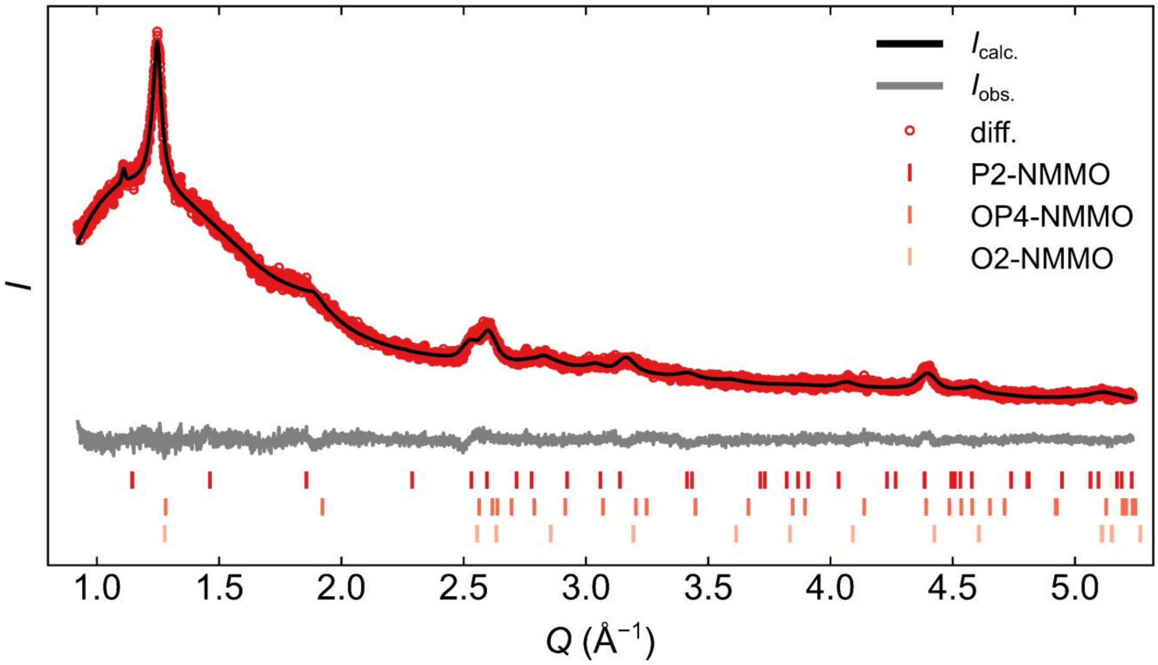


**Figure S11:** Rietveld refinement of the laboratory XRD pattern collected for an *ex situ* cathode of NMMO at point C5, recorded at room temperature ($R_{wp}$ = 4.0%).

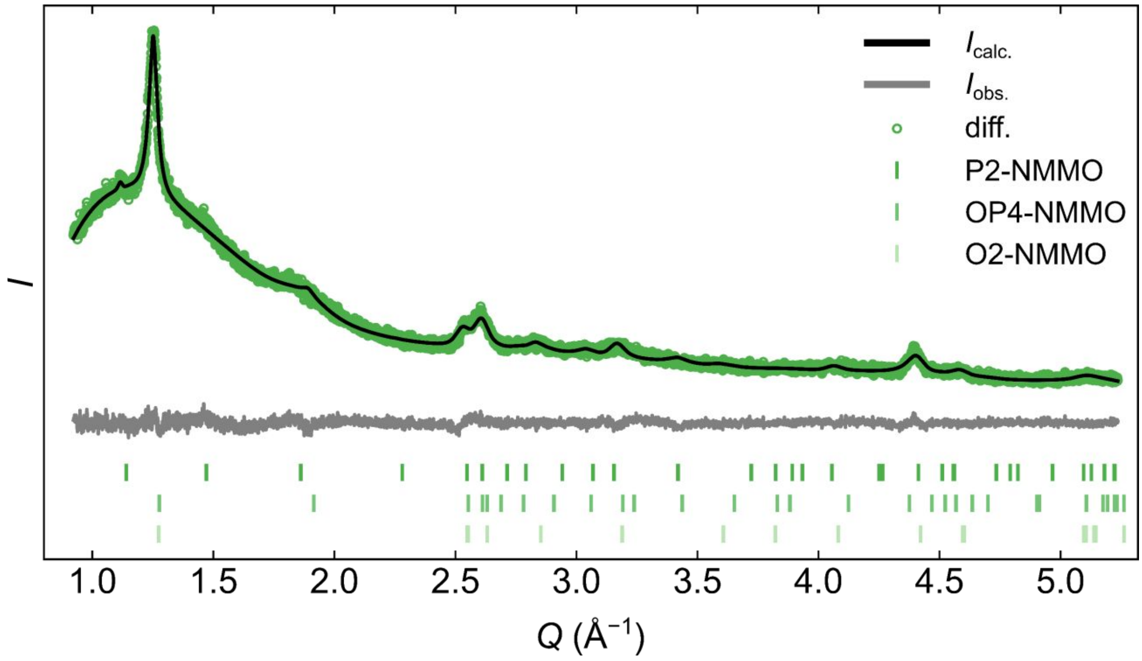


**Figure S12:** Rietveld refinement of the laboratory XRD pattern collected for an *ex situ* cathode of NMMO at point D1, recorded at room temperature ($R_{wp}$ = 3.9%).

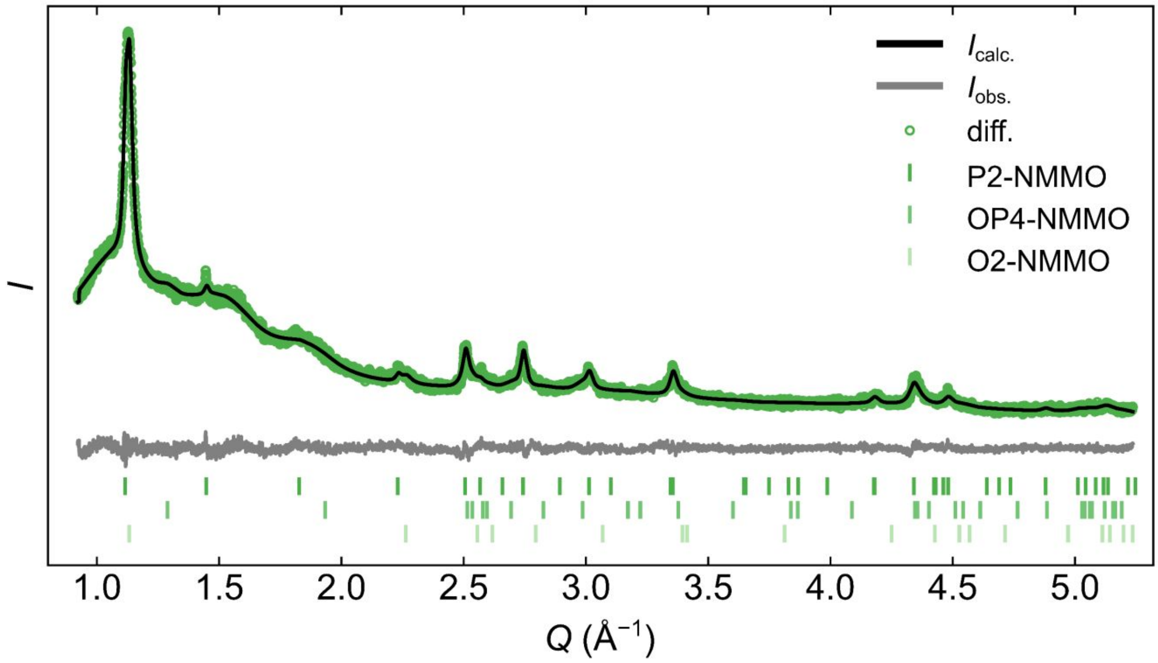


**Figure S13:** Rietveld refinement of the laboratory XRD pattern collected for an *ex situ* cathode of NMMO at point D2, recorded at room temperature ($R_{wp}$ = 4.1%).

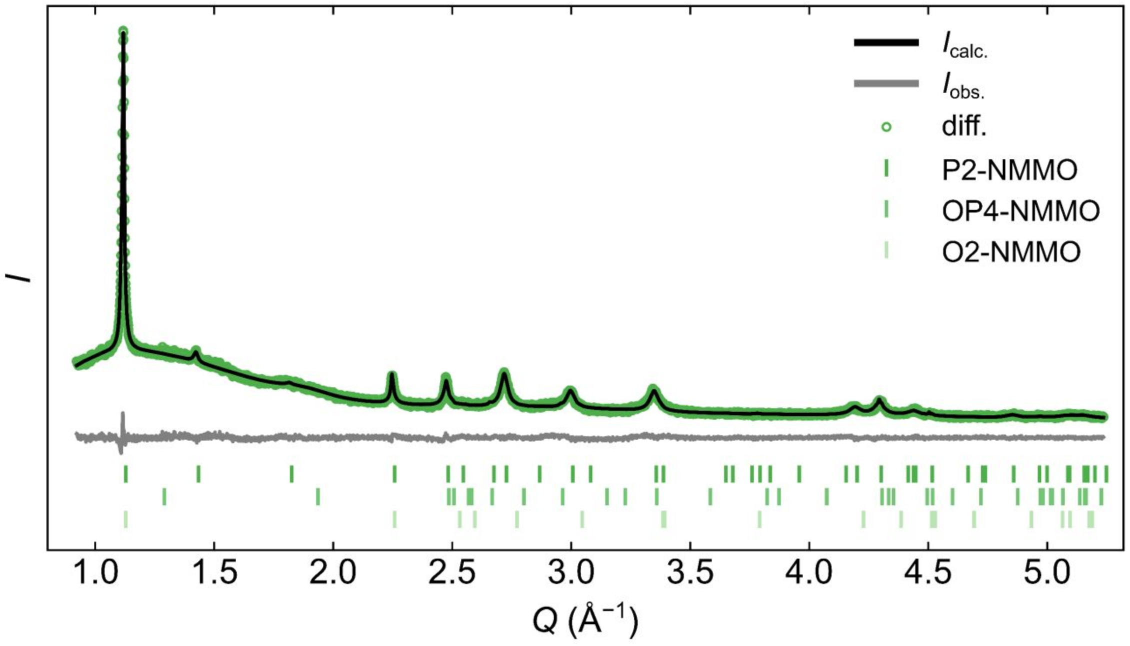


**Figure S14:** Rietveld refinement of the laboratory XRD pattern collected for an *ex situ* cathode of NMMO at point D3, recorded at room temperature ($R_{wp}$ = 4.2%). Note that the OP4 and O2 phase fractions were negligible: only 1 wt.% each.

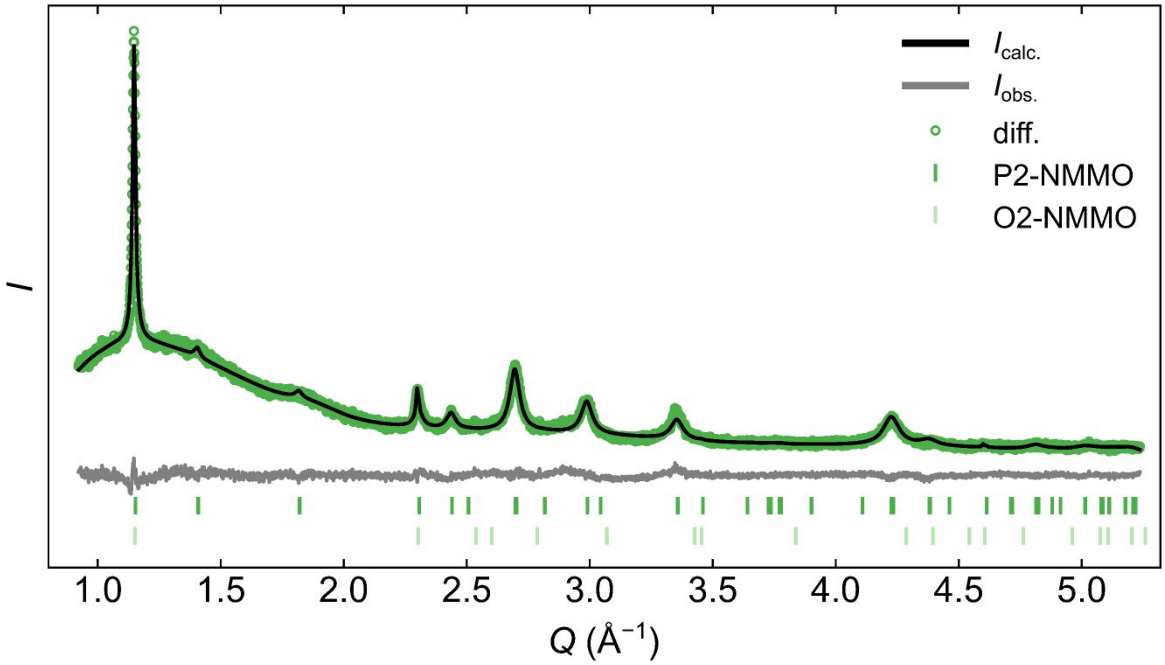


**Figure S15:** Rietveld refinement of the laboratory XRD pattern collected for an *ex situ* cathode of NMMO at point D4, recorded at room temperature ($R_{wp}$ = 4.9%). Note that the O2 phase fraction was negligible
(< 1 wt.%).

For the diffraction patterns observed from points C4 to D4 inclusive (i.e., all patterns refined against the O2 and/or OP4 phases), $Mg^{2+}$ was allowed to partially occupy tetrahedral sites in the O-type $Na^+$ layers. Inclusion of $Mg^{2+}$ in these O-type layers resulted in a small improvement of the fit ($R_{\text{w.p., Mg-migrated}}$ = 4.0%; $R_{\text{w.p., non-Mg-migrated}}$ = 4.1%; Figure S16). We also carried out refinements to structures in which $Mg^{2+}$ was allowed to occupy the vacant octahedral sites in

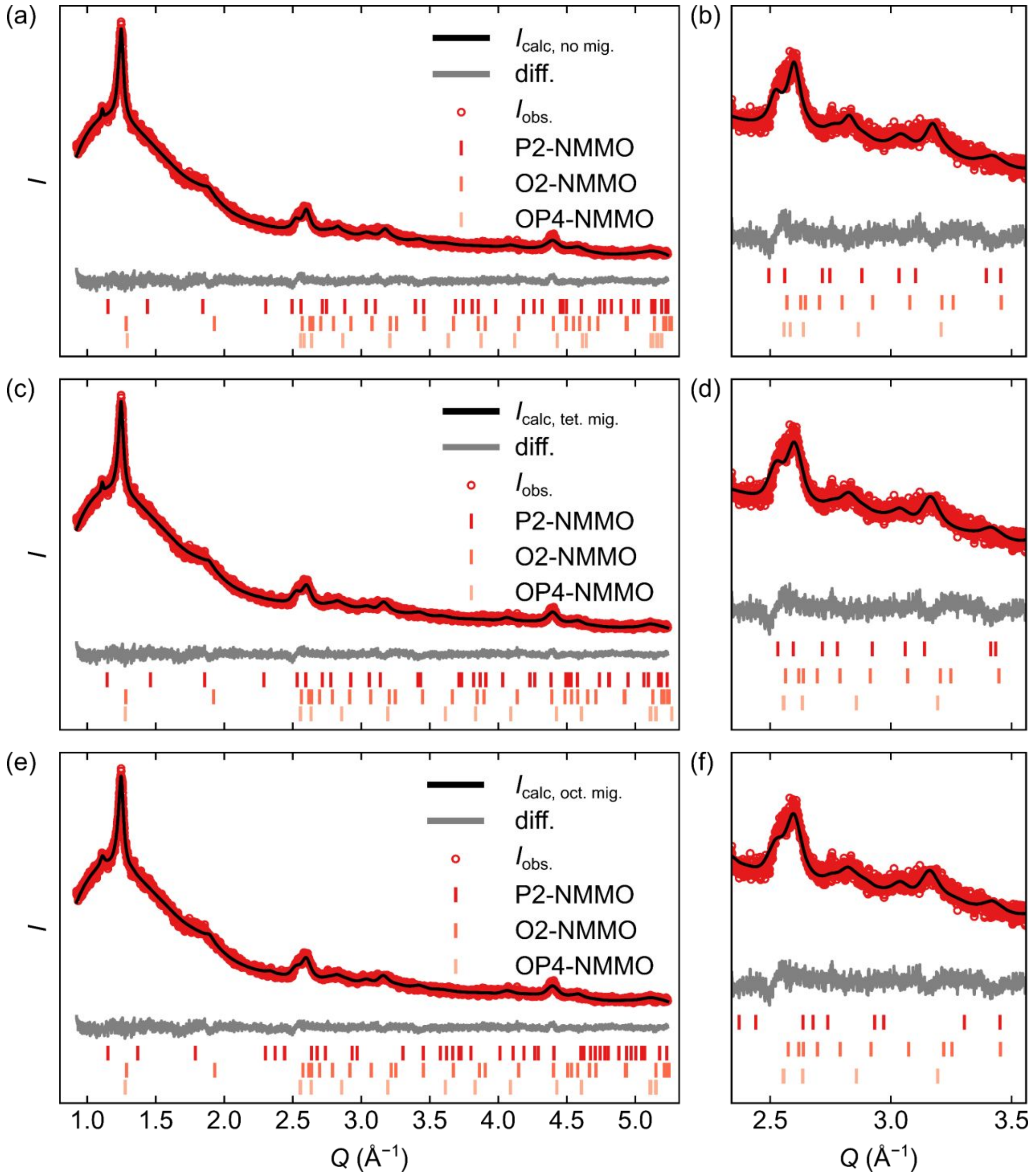


**Figure S16:** Rietveld refinements of the laboratory XRD pattern collected for an *ex situ* cathode of NMMO at point C5, recorded at room temperature. **(a)** shows the refinement against O2 and OP4 NMMO with no $Mg^{2+}$ migration (i.e., all $Mg^{2+}$ sits in the octahedral sites in the $TMO_2$ layers), whilst **(c)** shows the refinement against O2 and OP4 phases with $Mg^{2+}$ migration to the vacant tetrahedral sites in the O-type layers, and **(e)** shows the refinement against O2 and OP4 phases with $Mg^{2+}$ migration to the vacant octahedral sites in the O-type layers. To the right, **(b)**, **(d)** and **(f)** show expansions of the Rietveld fits in **(a)**, **(c)** and **(e)**, respectively, over the region $Q$ = 2.34 Å$^{-1}$ to $Q$ = 3.56 Å$^{-1}$, where the refinements show the largest differences in their fits to the data. For $Mg^{2+}$-migrated O2 and OP4, $R_{wp}$ = 4.0% (for both the tetrahedral and octahedral $Mg^{2+}$); for non-$Mg^{2+}$-migrated O2 and OP4, $R_{wp}$ = 4.1%.

the O-type layers of the O2 and OP4 phases [Figure S16(e)]; this resulted in a similar fit with $R_{w.p.}$ = 4.0%. Whilst this is not conclusive proof of $Mg^{2+}$ migration to the tetrahedral sites of the O-type layers, it does mean that our results are consistent with $Mg^{2+}$ migration.

# 7. Mn *K*-edge EXAFS Fits and Fitted Parameters

To establish the changes in the local structure around Mn during charge and discharge, Mn *K*-edge EXAFS data were recorded at the *ex situ* points C0, C2, C5 and D3. The data were fitted to scattering paths calculated from (2×2×2) supercells of the structures of the dominant phase(s) present at that state of charge (with lattice parameters from *ex situ* XRD refinements), using Feff in the Artemis software package. These fits are shown in Figures S17 to S20, with the fitted parameters (effective scattering distances and Debye-Waller factors) displayed in Table S2. In addition, fits to the data collected at the end of first charge (i.e., point C5) with and without $Mg^{2+}$ migration are shown in Figure S20.

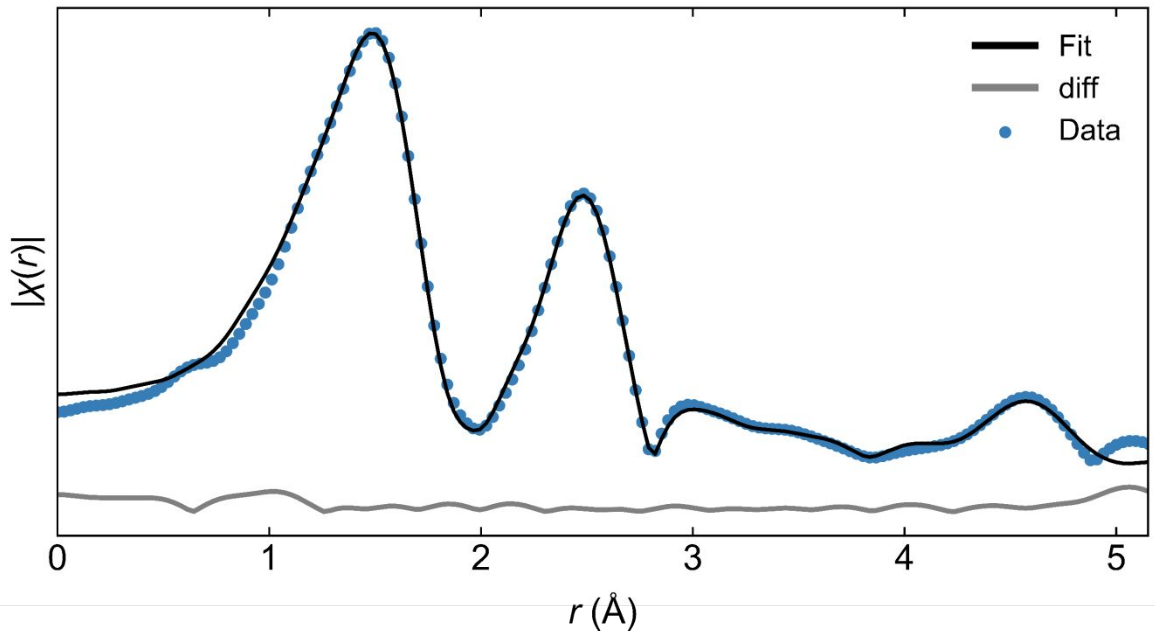


**Figure S17:** Fit to the Mn *K*-edge EXAFS data collected for pristine NMMO (point C0).

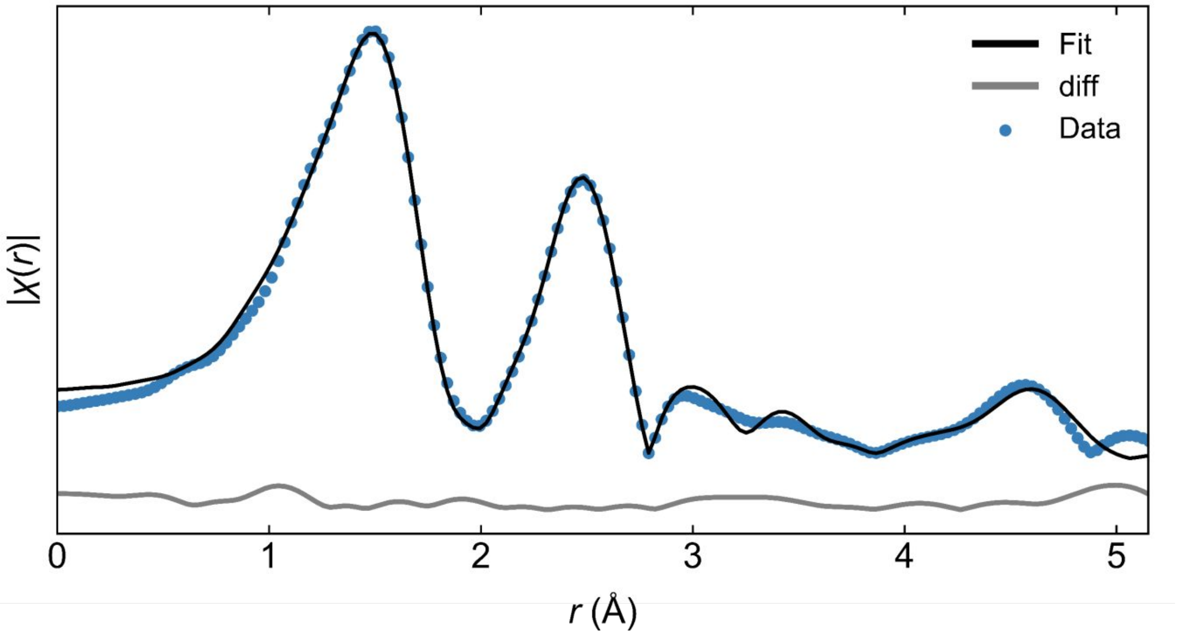


**Figure S18:** Fit to the Mn *K*-edge EXAFS data collected for NMMO at *ex situ* point C2.

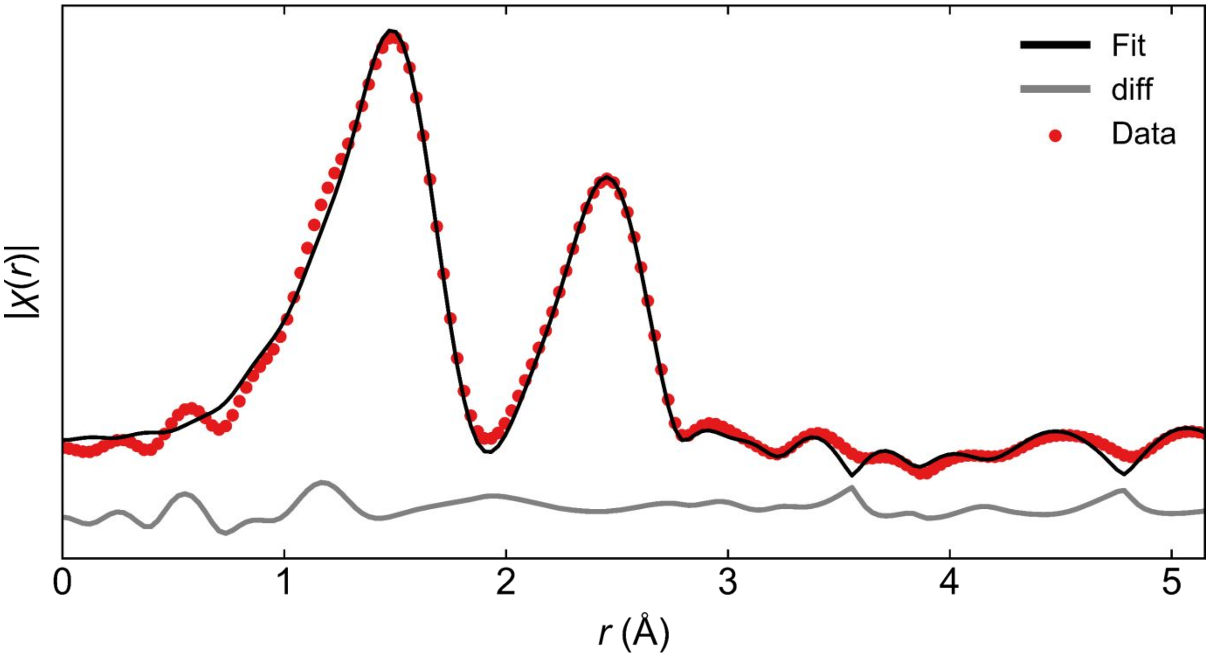


**Figure S19:** Fit to the Mn *K*-edge EXAFS data collected for NMMO at *ex situ* point C5.

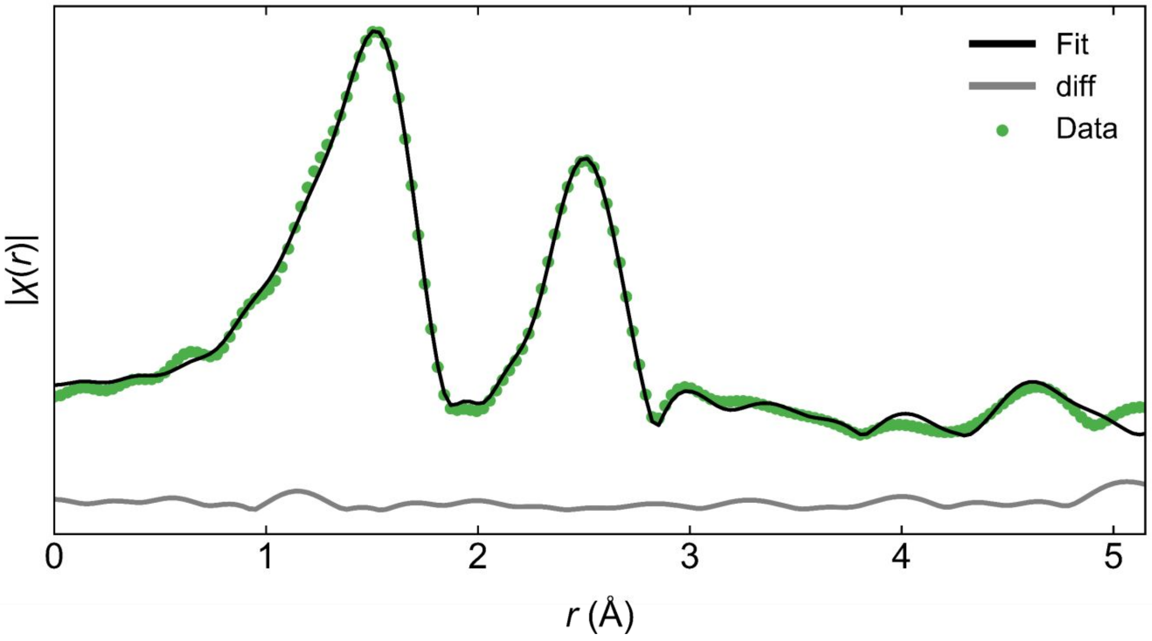


**Figure S20:** Fit to the Mn *K*-edge EXAFS data collected for NMMO at *ex situ* point D3.

**Table S2:** Scattering distances and Debye-Waller factors for the dominant single-scattering paths from each element, *X*, in NMMO (*X* = O, Mn, Mg, Na), extracted from *ex situ* EXAFS data fitting, with standard errors in parentheses. Values with asterisks indicate that the parameter was held constant during the fit. In the case of the Mn–Mg distance for sample C5, the distances to octahedral (oct.) and tetrahedral (tet.) $Mg^{2+}$ are given.

| Scattering Path | Sample | Phase | Effective Scattering Distance, $r_{Mn-X}$ (Å) | Debye-Waller Factor, $\sigma^2$ |
|---|---|---|---|---|
| Mn–O | C0 | P2 | 1.9227(11) | 0.0064(3) |
| | C2 | P2 | 1.9212(18) | 0.0062(5) |
| | C5 | O2 | 1.914* | 0.009(2) |
| | | OP4 | 1.902* | 0.007(2) |
| | D3 | P2 | 1.9221(16) | 0.0040(3) |
| Mn–Mn | C0 | P2 | 2.9056* | 0.0101(12) |
| | C2 | P2 | 2.92(2) | 0.0092(12) |
| | C5 | O2 | 2.87* | 0.0040(6) |
| | | OP4 | 2.83(1) | 0.009(11) |
| | D3 | P2 | 2.899(16) | 0.0152(17) |
| Mn–Mg | C0 | P2 | 2.836(7) | 0.0044(6) |
| | C2 | P2 | 2.823(13) | 0.0044(10) |
| | C5 | O2 | Oct.: 2.89(10) | 0.0004* |
| | | | Tet.: 3.65(2) | 0.0004* |
| | | OP4 | 2.81(2) | 0.006(2) |
| | D3 | P2 | 2.837(5) | 0.0037(4) |
| Mn–Na | C0 | P2 | 3.500(12) | 0.0115(8) |
| | C2 | P2 | 3.11(5) | 0.025(7) |
| | C5 | O2 | *No contribution* | *No contribution* |
| | | OP4 | *No contribution* | *No contribution* |
| | D3 | P2 | 3.085(17) | 0.017(2) |

As with the *ex situ* XRD Rietveld refinement of point C5, three fits were performed on the Mn *K*-edge EXAFS data at point C5: one with $Mg^{2+}$ having migrated to the vacant tetrahedral sites of the O-type layers in O2- and OP4-NMMO; one without $Mg^{2+}$ migration and one with $Mg^{2+}$ having migrated to the vacant octahedral sites of the O-type layers in O2- and OP4-NMMO [Figure S21]. Note that only O2 and OP4 phases are included in these fits, as these phases comprise the *Z*-phase which dominates at the end of first charge. The fit without $Mg^{2+}$ migration is slightly poorer than with $Mg^{2+}$ migration; between the migrated $Mg^{2+}$ fits, there is little difference between the fits. Therefore, our data is consistent with $Mg^{2+}$ migration, but is not conclusive.

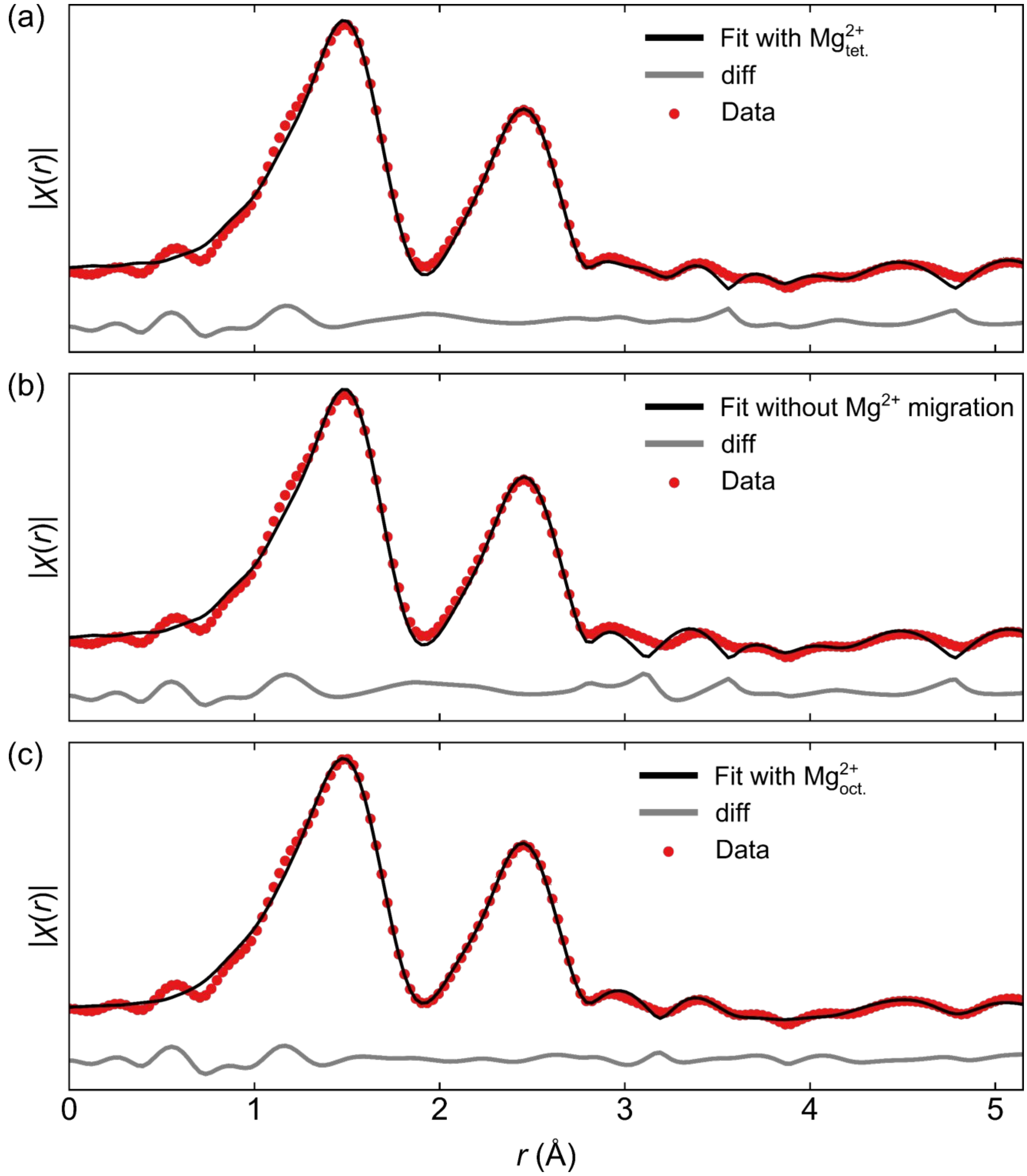


**Figure S21:** Fits to the Mn *K*-edge EXAFS data collected for NMMO at *ex situ* point C5: **(a)** with $Mg^{2+}$ migration to the tetrahedral sites in the vacant O-type layers of the O2 and OP4 phases; **(b)** without $Mg^{2+}$ migration; and **(c)** with $Mg^{2+}$ migration to the octahedral sites in the vacant O-type layers of the O2 and OP4 phases.

## 8. Additional $^{23}Na$ NMR Results

The longitudinal and transverse relaxation times, $T_1$ and $T_2$, respectively, for *ex situ* samples of NMMO were measured using saturation recovery and two-dimensional Hahn-echo pulse sequences, respectively. These relaxation times were difficult to quantitatively interpret, due to the low signal-to-noise or breadth of the resonances being probed. We anticipate that the $T_1$ times depend on the changes to electronic structure and fluctuating hyperfine interactions as $Na^+$ ions hop, whilst the $T_2$ time likely depends on the fluctuating hyperfine interaction and the rate at which $Na^+$ ions hop between sites. The $T_1$ relaxation times do not appear to correlate with the number of or spin of the paramagnetic species anticipated expected at each state of charge. The $T_2$ times broadly correlate with the $Na^+$ motion regimes seen: slow relaxation times and broad, low intensity resonances are seen for slow $T_2$ times (above approximately 150 μs), whilst sharp resonances are generally seen for faster $T_2$ times. The $T_2$ at point D3 was unusually fast; repeated measurements indicate that this is a genuine feature, but the origin of this fast relaxation is unknown. Further variable-temperature analyses of the transverse relaxation rates and the spectra observed at each state of charge will be the topic of a future study.

**Table S3:** Longitudinal and transverse relaxation times ($T_1$ and $T_2$) measured for *ex situ* NMR samples of $Na_x[Mg_{0.28}Mn_{0.72}]O_2$ at different MAS rates, alongside the average hyperfine shift under 60 kHz MAS is also listed; note that values in parentheses are estimated standard errors. Values with asterisks denote quantities which were difficult to determine, due to the low signal-to-noise ratio (C1 and C5) or breadth (D4) of the signal. Note that the relaxation times for sample D1 were not recorded but are anticipated to be very similar to those for C5.

| Sample label | $T_1$ (ms) | $T_2$ (μs) | MAS Rate (kHz) | Average Hyperfine Shift at 60 kHz MAS (ppm) |
|---|---|---|---|---|
| C0 | 1.7(2) | 60(5) | 60 | 1430(5) |
| C1 | 1.8(5)* | 190*(10) | 40 | 1390(5) |
| C2 | 1.0(2) | 70(5) | 40 | 1455(5) |
| C3 | 1.0(2) | 90(5) | 40 | 1475(5) |
| C4 | 1.0(2) | 117(5) | 60 | 1510(5) |
| C5 | 1.7(5)* | 270(10)* | 40 | 1480(5) |
| D1 | - | - | - | 1485(5) |
| D2 | 1.5(2) | 150(5) | 60 | 1475(5) |
| D3 | 1.2(2) | 20(5) | 60 | 1500(5) |
| D4 | 2.3(5)* | 120(10)* | 60 | 1360(5) |

The $^{23}Na$ NMR Hahn-echo spectra shown in Figure 5 of the main text contain spinning sidebands. In many paramagnetic NIB and LIB cathodes, these sidebands can overlap with the isotropic resonances and obscure them, making interpretation of the spectra challenging. To isolate the isotropic resonance from the spinning sideband manifold, a projected magic angle turning phase-adjusted sideband separation (pjMATPASS) was carried out on each sample. The pjMATPASS experiment is a two-dimensional experiment, with delays between pulses in the pulse sequence set according to the PASS equations, such that in each slice of the spectrum, a sideband is isolated. The intensity transferred from the isotropic resonance to each sideband can then be restored to the isotropic resonance by 'tilting' the two-dimensional array of data and summing the intensities of all rows. This produces a two-dimensional spectrum, such as that shown in Figure S22 for pristine NMMO.

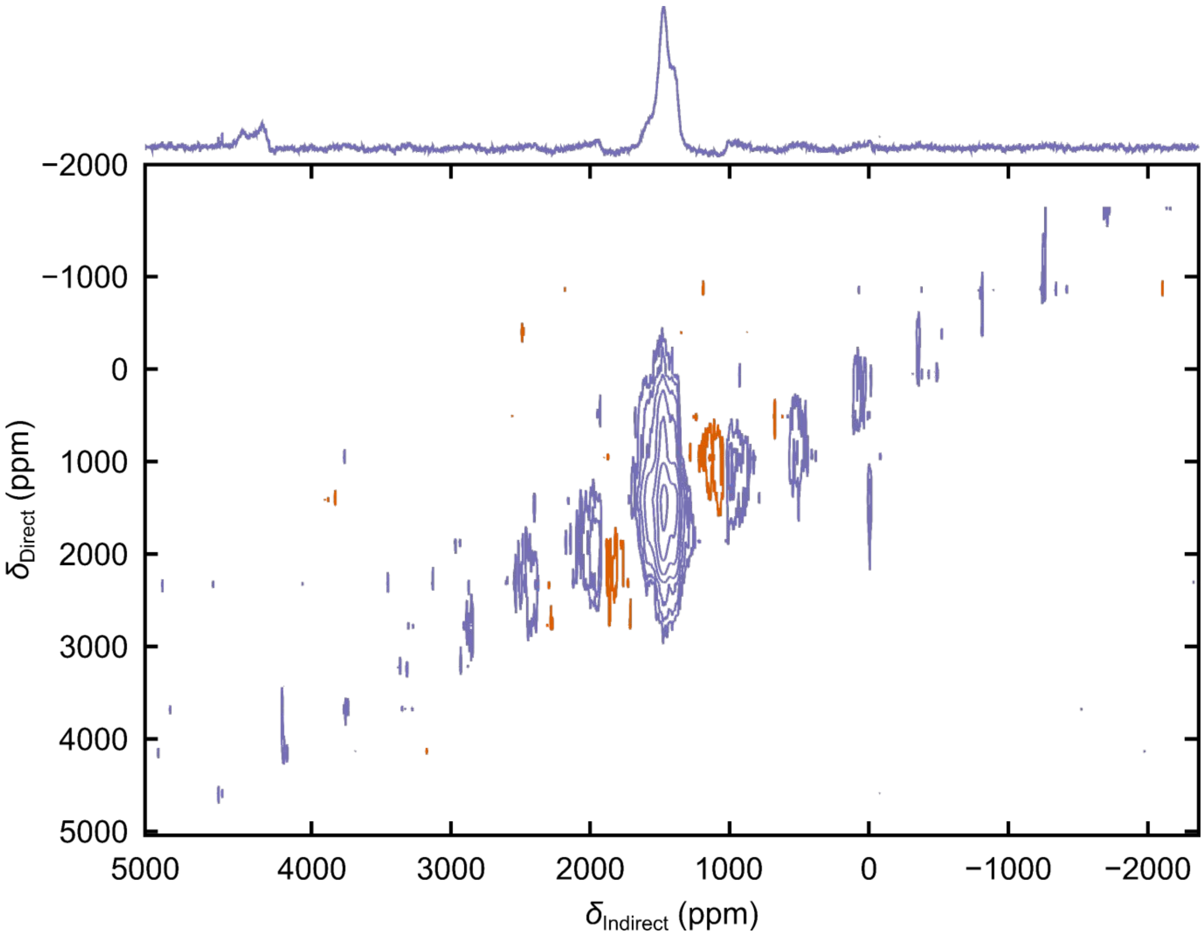


**Figure S22:** Two-dimensional $^{23}Na$ NMR pjMATPASS spectrum for pristine NMMO, acquired at 11.7 T under a MAS rate of 60 kHz with recycle delay of 7.5 ms. The sidebands seen in the skyline projection plotted above the two-dimensional spectrum, which appear to originate from incomplete sideband suppression, can clearly be seen to arise from a broadening of the sidebands in the direct dimension. The resonance at approximately 4400 ppm is due to Cu metal from the coil/probe.

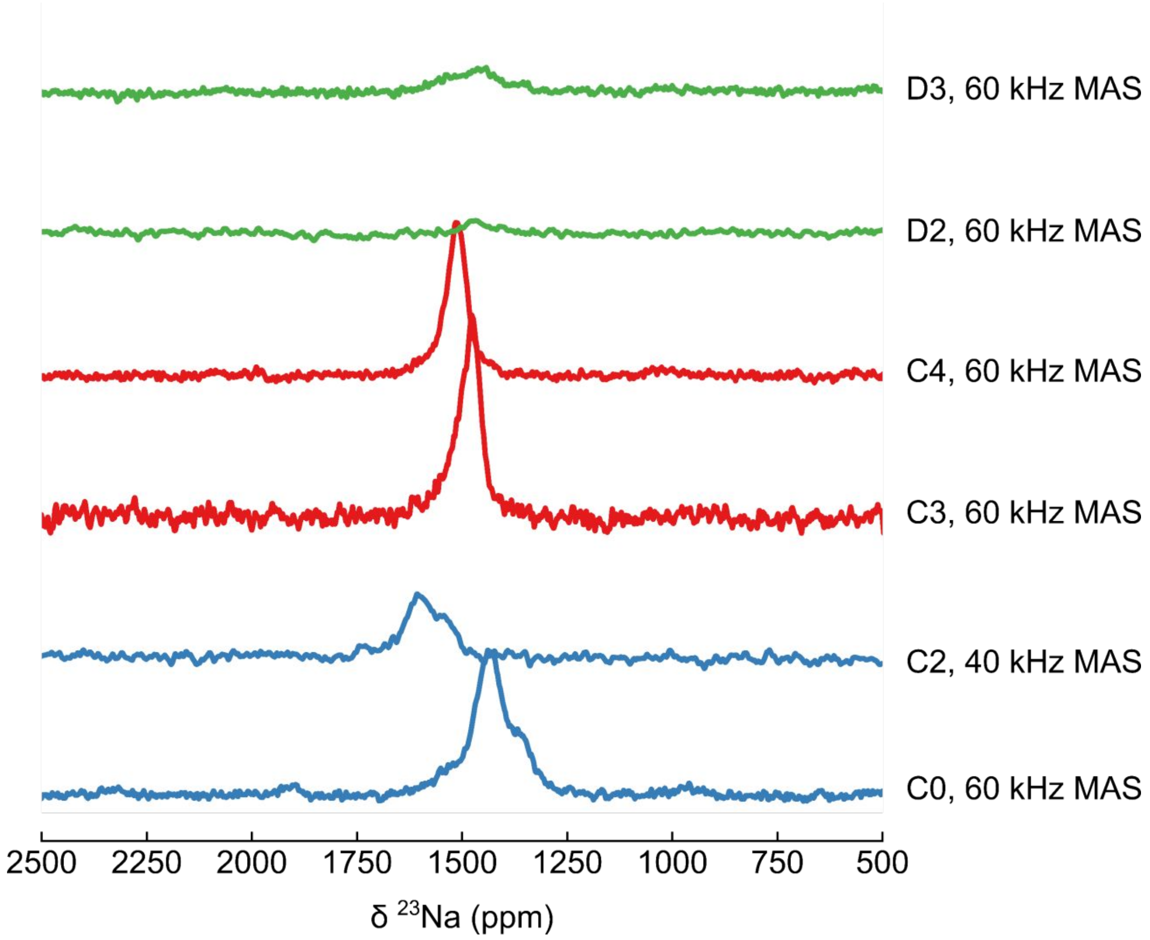


**Figure S23:** One-dimensional 'tilted' $^{23}$Na NMR pjMATPASS spectra of NMMO at different states of charge. All spectra were acquired at 11.7 T with MAS rate indicated to the right of the spectrum. The spectral intensities are scaled according to sample mass and number of scans.

The 'tilted' spectrum (shown at the top of Figure S22) can then be plotted as a conventional one-dimensional spectrum, as shown in Figure S23 for a series of *ex situ* samples of NMMO.

Unfortunately, due to the low signal intensities seen for samples C1, C5 and D1, the pjMATPASS gave a spectrum with no intensity in any region of the spectrum. We therefore assigned the isotropic resonances of these samples by changing the MAS rate to 40 kHz and comparing the spectrum with the spectrum acquired under 60 kHz MAS [Figure S24]. Note that the spectra recorded at 40 kHz have different shifts than those at 60 kHz, due to the change in sample temperature with spinning speed (a consequence of frictional heating from spinning). To compare the shifts between the spectra at different MAS rates (i.e., different temperatures), a Curie-Weiss law scaling parameter was used (see section 9), assuming that the sample is at 318 K under 60 kHz MAS rates and at 303 K for 40 kHz MAS rates.[32]

For sample D4, no spinning sidebands were observed in the Hahn-echo spectrum (other than those due to diamagnetic electrolyte decomposition products), due to the large number of environments, so we did not carry out pjMATPASS or variable spinning speed measurements.

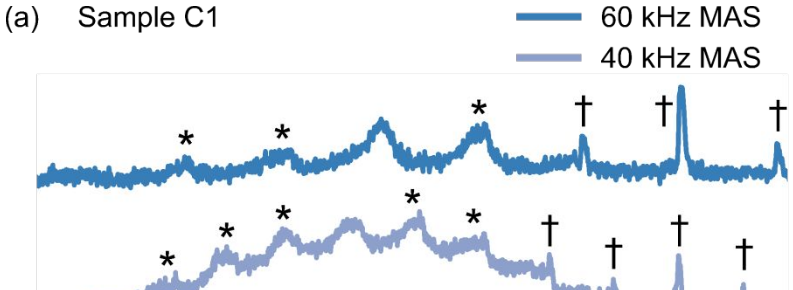

To identify whether $Na^+$ ion motion in NMMO affects the $^{23}Na$ NMR spectra observed, we carried out variable-temperature NMR experiments on pristine NMMO and at *ex situ* point C1 [Figures S25 and S26]. Note that the MAS rate for these experiments was limited to 28 kHz due to the rotor size (2.5 mm) and to ensure stable spinning whilst varying the temperature. As a result, the low-frequency resonance seen under 60 kHz MAS could not be observed

under 28 kHz (apart from at high temperature, 378 K), due to poor spatial averaging of the hyperfine tensor for the spins giving rise to this resonance.

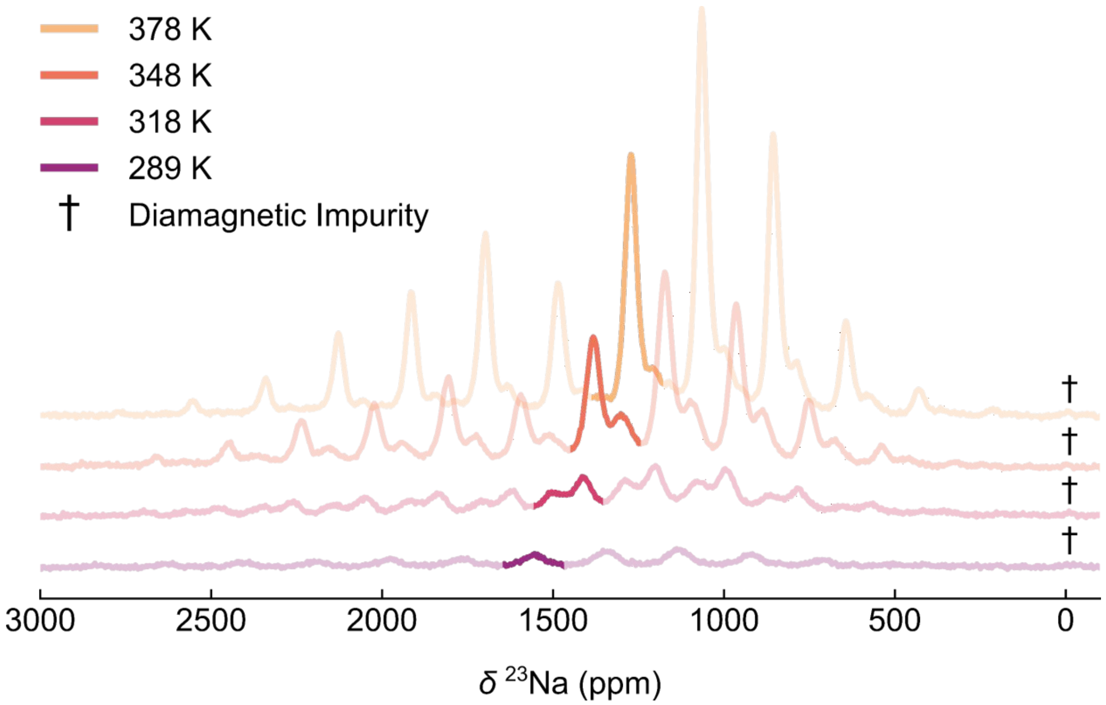


**Figure S25:** Variable-temperature $^{23}Na$ NMR spectra for pristine NMMO, recorded at a field strength of 11.7 T under a 28 kHz MAS rate. The isotropic resonances are shown in full colour, whilst the sideband manifold is shown faded, for the sake of clarity. All spectra are normalised to the number of scans.

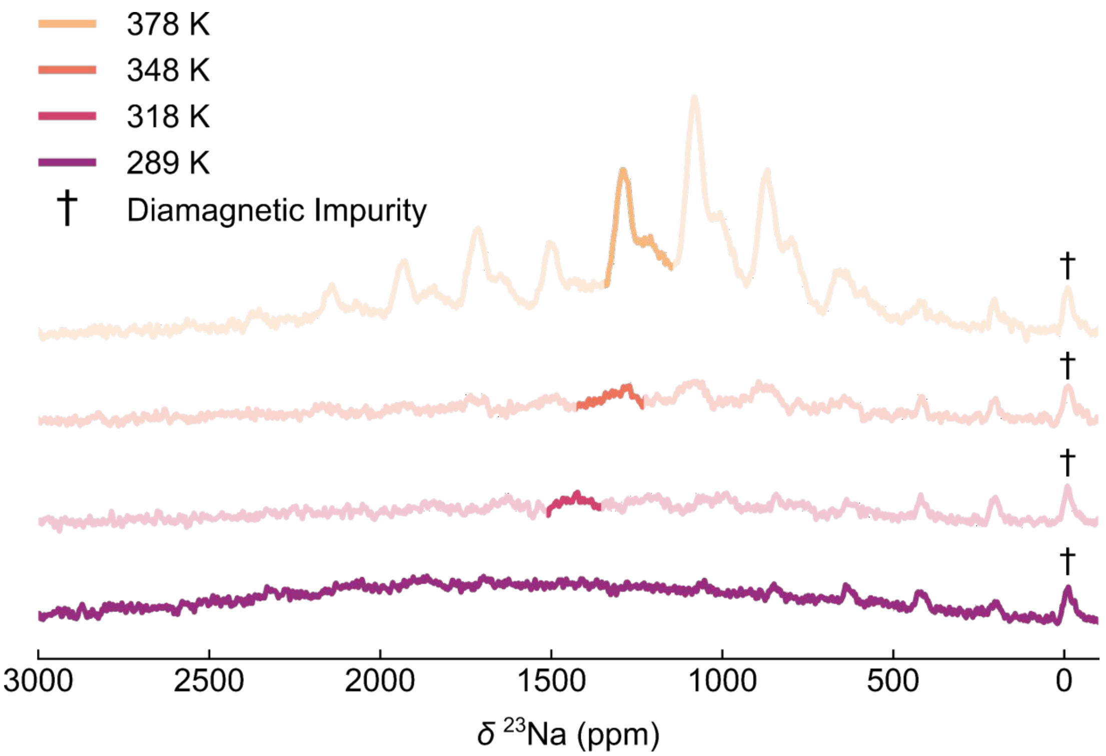


**Figure S26:** Variable-temperature $^{23}Na$ NMR spectra for an *ex situ* sample of NMMO at point C1, recorded at a field strength of 11.7 T under a 28 kHz MAS rate. The isotropic resonances are shown in full colour, whilst the sideband manifold is shown faded, for the sake of clarity. Note that the isotropic resonance for the spectrum at 286 K could not be determined, so the entire spectrum is shown in full colour. All spectra are normalised to the number of scans.

To determine the temperature of the sample, *ex situ* temperature calibration was carried out using $^{207}Pb$ shift of $Pb(NO_3)_2$ under the same heater powers, nitrogen gas flow rates, and driving and bearing pressures used for $^{23}Na$ NMR experiments. The shift was assumed to

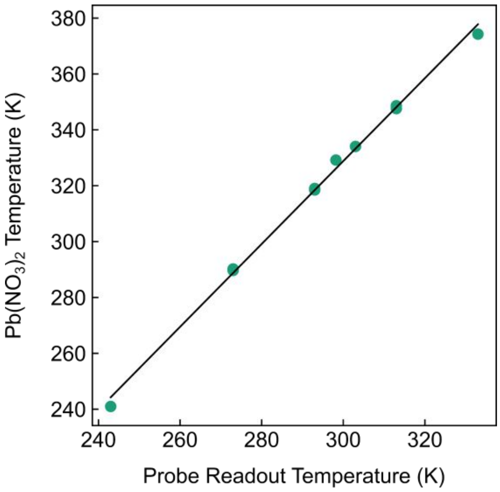


**Figure S27:** Variable-temperature NMR temperature calibration curve, obtained from the $^{207}Pb$ shift of $Pb(NO_3)_2$.

depend on temperature linearly using the curve from refs. [33,34]. The calibration curve is shown in Figure S27.

## 9. *Ab initio* $^{23}$Na NMR Shift Calculations

The hyperfine and quadrupolar contributions to the $^{23}$Na NMR shifts was calculated using the methods detailed in refs. 7 and 8. The total shift for each $^{23}$Na site is the sum of the hyperfine shift (obtained from the sum of bond pathways) and the quadrupole-induced shift.

### Hyperfine Shift Calculations

The hyperfine (Fermi contact) shift induced on a nucleus due to a nearby paramagnetic *TM* centre may be calculated from the electron spin density, $|\psi^{\alpha-\beta}(\boldsymbol{R}_{\mathrm{N}})|^2$, at the nuclear position of the nucleus of interest:

$$\delta_{\mathrm{iso}} = \frac{2SA_{\mathrm{iso}}\chi_{\mathrm{M}}}{N_{\mathrm{A}}\mu_{\mathrm{B}}\mu_0 g_{\mathrm{e}} g_{\mathrm{N}}\mu_{\mathrm{N}}},$$

where $\chi_M$ is the molar magnetic susceptibility of the material; $N_A$ is Avogadro's number; $g_e$ and $g_N$ are the electron and nuclear *g*-factors, respectively; $\mu_B$ is the Bohr magneton; $\mu_N$ is the nuclear magneton; $\mu_0$ is the permeability of free space and *S* is the total electronic spin. $A_{iso}$ is the hyperfine coupling constant:

$$A_{\mathrm{iso}} = \frac{\mu_0\mu_{\mathrm{B}}\mu_{\mathrm{N}} g_{\mathrm{e}} g_{\mathrm{N}} |\psi^{\alpha-\beta}(\boldsymbol{R}_{\mathrm{N}})|^2}{3S},$$

In practice, $\delta_{\mathrm{iso}}$ is calculated for a fictitious saturated ferromagnetic ground state at $T$ = 0 K. To account for the effect of thermal randomisation on the spin orientations, a Curie-Weiss scaling parameter, $\Phi$, is applied to the spin density:

$$\Phi = \frac{M_T}{M_{\mathrm{sat.}}},$$

where $M_{\mathrm{sat.}}$ and $M_T$ are the magnetisations when the sample is in the fictitious saturated ferromagnetic ground state and the thermally randomised state, respectively. This may be expanded using the Curie-Weiss law:

$$\Phi = \frac{B_0\mu_{\mathrm{eff.}}^2}{3k_{\mathrm{B}} g_{\mathrm{e}}\mu_{\mathrm{B}}(T-\theta)},$$

where $\mu_{\mathrm{eff.}}$ is the effective magnetic moment, $\theta$ is the Weiss constant, $B_0$ the magnetic field strength used in the NMR experiment, $g_e$ the free electron *g*-factor, $k_B$ the Boltzmann constant and $\mu_B$ the Bohr magneton. In this work, the experimentally observed Weiss constant and effective magnetic moment were used (see section 9). This scaling parameter may then be used to modify the hyperfine shift:

$$\delta_{\mathrm{iso}} = \frac{A_{\mathrm{iso}}}{h\nu_0}\Phi,$$

where *h* is Planck's constant and $\nu_0$ is the Larmor frequency (in Hz) of the observed nucleus.

The extent of spin transfer—and therefore the value of $\delta_{\mathrm{iso}}$—depends on the degree of orbital overlap along the bond pathway between the *TM* ion and *X* and is therefore highly sensitive to TM orbital occupancy and the *TM*–*X* bond angles and distances. The relative magnitudes and signs of the spin density transferred may be qualitatively rationalised using the Goodenough-Kanamori rules.[35–37] For $Na_{0.67}[Mg_{0.28}Mn_{0.72}]O_2$, spin density is transferred from the $Mn^{3+}$ and $Mn^{4+}$ *d* orbitals ($e_g$ and $t_{2g}$ for $Mn^{3+}$; $t_{2g}$ only for $Mn^{4+}$), to the $Na^+$ 3*s* orbital, *via* an intermediary 2*p* orbital on $O^{2-}$.

To simplify calculations and account for the partial Na occupancies in NMMO, a model all-$Mn^{4+}$, Mg/Mn honeycomb-ordered system, P2-$Na_{2/3}[Mg_{1/3}Mn_{2/3}]O_2$, was constructed to determine the approximate shifts expected for NMMO. Throughout these calculations, a (2×1×2) supercells were used to account for as many exchange interactions between $Mn^{4+}$ centres as possible. Attempts to dope $Mn^{3+}$ into the cell—either by doping a polaron onto a single Mn centre, adding a $Na^+$ to the cell or swapping one $Mg^{2+}$ for $Mn^{3+}$ and removing one $Na^+$, were unsuccessful, due to SCF convergence problems.

The Fermi contact shifts for Na–O–$Mn^{4+}$ bond pathways in the P(2d) and P(2b) sites are shown in Figure S28. From this, the total Fermi contact shifts for all possible $Na^+$ sites were computed by iterating through all possible bond pathway combinations [Table S4].

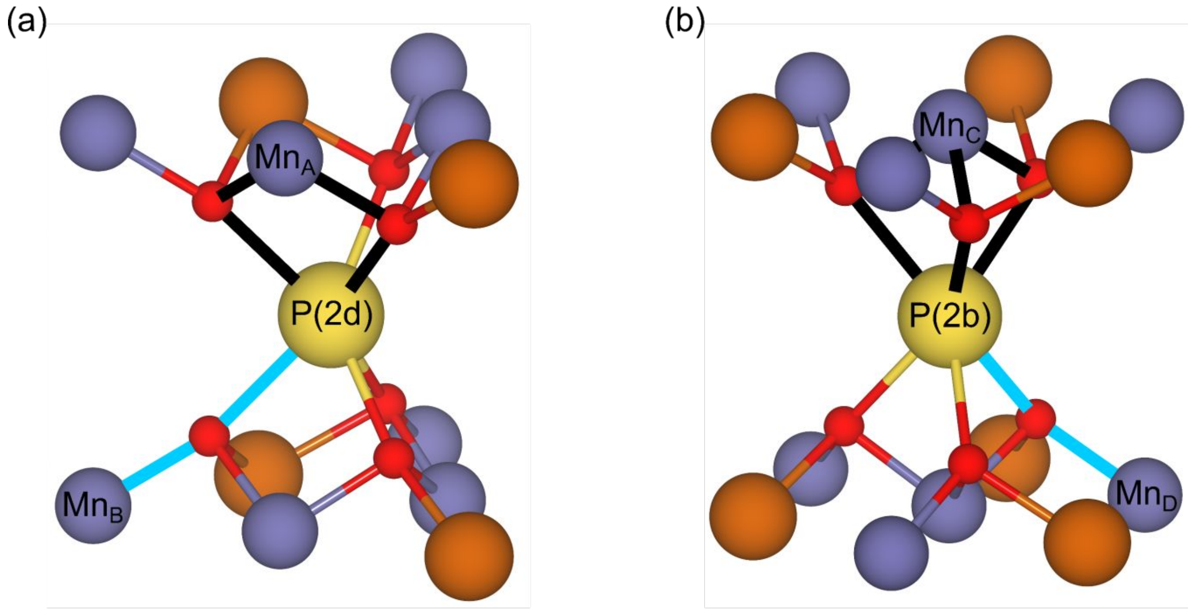


| | Hyb35 | Hyb20 |
|---|---|---|
| $Mn_A^{4+}$ | +459 ppm | +542 ppm |
| $Mn_B^{4+}$ | −12 ppm | −15 ppm |

| | Hyb35 | Hyb20 |
|---|---|---|
| $Mn_C^{4+}$ | −268 ppm | −222 ppm |
| $Mn_D^{4+}$ | +95 ppm | +104 ppm |

**Figure S28:** First-principles Na–O–$Mn^{4+}$ bond pathway contributions to the $^{23}Na$ Fermi contact shift, calculated for P2-$Na_{2/3}[Mg_{1/3}Mn_{2/3}]O_2$ ($a \approx 10.20$ Å, $b \approx 5.10$ Å, $c \approx 22.86$ Å), using hybrid 35 and hybrid 20 functionals. **(a)** shows the bond pathways for a P(2d) $Na^+$ site, whilst **(b)** shows the bond pathways for a P(2b) $Na^+$ site.

## Quadrupolar Shift Calculations

The quadrupolar interactions for $^{23}Na$ are significant in NMMO (see Table S4 for calculated quadrupolar parameters), leading to a quadrupole-induced shift, $\delta_{QIS}$. The principal components of the electric field gradient at each $^{23}Na$ nucleus were computed alongside the spin densities, using the method detailed above. The principal axes were ordered such that $|V_{22}| \leq |V_{11}| \leq |V_{33}|$.

From the principal components electric field gradient tensor, the quadrupolar asymmetry parameter, $\eta_Q$, was computed:

$$\eta_Q = \frac{V_{22} - V_{33}}{V_{11}}$$

From this, the value of $\delta_{QIS}$ was computed using:

$$\delta_{QIS} = -\frac{\nu_Q^2}{30\omega_0}\left[I(I+1) - \frac{3}{4}\right]\left(1 + \frac{\eta_Q^2}{3}\right),$$

where $\omega_0$ is the nuclear Larmor frequency in rad $s^{-1}$ and $\nu_Q$ is the quadrupolar frequency (in Hz):

$$\nu_Q = \frac{3C_Q}{2I(2I-1)},$$

**Table S4:** *Ab initio* calculated quadrupolar coupling constants, quadrupolar asymmetries and quadrupole-induced shifts for $Na_{2/3}[Mg_{1/3}Mn_{2/3}]O_2$ ($a \approx 10.20$ Å, $b \approx 5.10$ Å, $c \approx 22.86$ Å), using hybrid 35 and hybrid 20 functionals. Note that these parameters are averages across all sites (P(2d) and P(2b)), as there was essentially no difference between the values of $C_Q$, $\eta_Q$ and $\delta_{QIS}$ for the two sites.

| | Hyb35 | Hyb20 |
|---|---|---|
| $C_Q$ (MHz) | 3.21 | 3.25 |
| $\eta_Q$ | 0.051 | 0.047 |
| $\delta_{QIS}$ (ppm) | −9.5 | −9.7 |

and $C_Q$ is the quadrupolar coupling constant in Hz.

## Simulating $^{23}Na$ NMR Spectra

The hyperfine $Mn^{4+}$–O–$Na^+$ bond pathways calculated using the honeycomb-ordered model structure enable the isotropic hyperfine shift of any local environment in P2-$Na_{2/3}[Mg_{1/3}Mn_{2/3}]O_2$ to be determined by summing the bond pathway shifts created by all nearest and next-nearest neighbours (n.n. and n.n.n., respectively) to $Na^+$ in the *TM*$O_2$ layers adjacent to the $Na^+$ layer. Below, we have simulated the $^{23}Na$ NMR spectrum of P2-$Na_{2/3}[Mg_{1/3}Mn_{2/3}]O_2$ assuming: (i) a random arrangement of $Mg^{2+}$ and $Mn^{4+}$ ions over the *TM* sublattice (i.e., any site can be occupied by $Mg^{2+}$ and $Mn^{4+}$, with the probability of occupation being 1/3 and 2/3, respectively; Figure S29) and (ii) a honeycomb arrangement of $Mn^{4+}$ and $Mg^{2+}$ ions [Figure S30]. In both cases, we have assumed $Na^+$ ions are fixed and do not move from their sites during the experiment.

The random arrangement of $Mn^{4+}$ and $Mg^{2+}$ ions generates 49 possible Na P(2d) sites and 39 possible Na P(2b) sites. The probability (and therefore the intensities) of each site occurring is given by the product of:

- the probabilities that there are *n* $Mg^{2+}$ ions and *m* $Mn^{4+}$ ions are in the n.n. and n.n.n. coordination shells,
- the multiplicity of these arrangements (for P(2d), the multiplicity is ${}^{6}C_m$ for the n.n. and n.n.n. shells; for P(2b), the multiplicities are ${}^{2}C_m$ for the n.n. shell and ${}^{12}C_m$ for the n.n.n. shell),
- and the probability of occupying one of the crystallographic Na sites (for P(2d), this is 0.6 and for P(2b), this is 0.4; these probabilities are based on the multiplicities of the P(2d) and P(2b) sites in the crystal structure of pristine NMMO).

Calculating these probabilities for all P(2d) and P(2b) sites and weighting the intensities of the peaks in the simulated spectrum by these probabilities gives the spectrum seen in Figure S29. Here, a range of shifts from 3000 ppm to −190 ppm are seen. The most probable sites to be occupied are:

- for P(2d), four $Mn^{4+}$ n.n., two $Mg^{2+}$ n.n., four $Mn^{4+}$ n.n.n. and two $Mg^{2+}$ n.n.n., with Hyb35 shift of 1785 ppm and Hyb20 shift of 2108 ppm and overall probability 0.065;
- for P(2b): either (i) two $Mn^{4+}$ n.n., zero $Mg^{2+}$ n.n., eight $Mn^{4+}$ n.n.n. and four $Mg^{2+}$ n.n.n., Hyb35 shift of 228 ppm and Hyb20 shift of 387 ppm, or (ii) one $Mn^{4+}$ n.n., one $Mg^{2+}$ n.n., eight $Mn^{4+}$ n.n.n. and four $Mg^{2+}$ n.n.n., Hyb35 shift of 496 ppm and Hyb20 shift of 609 ppm. Both have probability 0.042.

When combined with the calculated quadrupole-induced shifts, none of the calculated shifts match those observed. The shifts which are closest are P(2d) sites with three $Mn^{4+}$ n.n., three

**Table S5:** *Ab initio* calculated hyperfine shifts of the P(2d) sites for $Na_{2/3}[Mg_{1/3}Mn_{2/3}]O_2$ (with a random distribution of $Mn^{4+}$ and $Mg^{2}$ cations) which were closest to the isotropic shifts seen for pristine NMMO. The shifts were obtained by summing the bond pathway shifts (calculated using hybrid 20 and hybrid 35 functionals) for each site. Note: n.n. = nearest neighbour and n.n.n. = next-nearest neighbour.

| Site type | No. of $Mn^{4+}$ n.n. | No. of $Mg^{2+}$ n.n. | No. of $Mn^{4+}$ n.n.n. | No. of $Mg^{2+}$ n.n.n. | Hyb35 Shift (ppm) | Hyb20 Shift (ppm) | Probabilit y |
|---|---|---|---|---|---|---|---|
| P(2d) | 3 | 3 | 6 | 0 | 1302 | 1537 | 0.0193 |
| P(2d) | 3 | 3 | 5 | 1 | 1314 | 1552 | 0.0578 |
| P(2d) | 3 | 3 | 4 | 2 | 1327 | 1566 | 0.0723 |
| P(2d) | 3 | 3 | 3 | 3 | 1339 | 1581 | 0.0482 |
| P(2d) | 3 | 3 | 2 | 4 | 1351 | 1596 | 0.0181 |
| P(2d) | 3 | 3 | 1 | 5 | 1364 | 1610 | 0.0036 |
| P(2d) | 3 | 3 | 0 | 6 | 1376 | 1625 | 0.0003 |

$Mg^{2+}$ n.n. and between zero and six $Mn^{4+}$ n.n.n. [Table S5].

**Table S6:** *Ab initio* calculated hyperfine shifts and assigned probabilities of the Na P(2d) and P(2b) sites for honeycomb-ordered $Na_{2/3}[Mg_{1/3}Mn_{2/3}]O_2$. The shifts were obtained by summing the bond pathway shifts (calculated using hybrid 20 and hybrid 35 functionals) for each site. Note: n.n. = nearest neighbour and n.n.n. = next-nearest neighbour.

| Site type | No. of $Mn^{4+}$ n.n. | No. of $Mg^{2+}$ n.n. | No. of $Mn^{4+}$ n.n.n. | No. of $Mg^{2+}$ n.n.n. | Hyb35 Shift (ppm) | Hyb20 Shift (ppm) | Probabilit y |
|---|---|---|---|---|---|---|---|
| P(2d) | 4 | 2 | 4 | 2 | 1785 | 2108 | 0.6 |
| P(2b) | 2 | 0 | 6 | 6 | 37 | 179 | 0.133 |
| P(2b) | 1 | 1 | 9 | 3 | 591 | 713 | 0.133 |
| P(2b) | 0 | 2 | 12 | 0 | 1145 | 1247 | 0.133 |

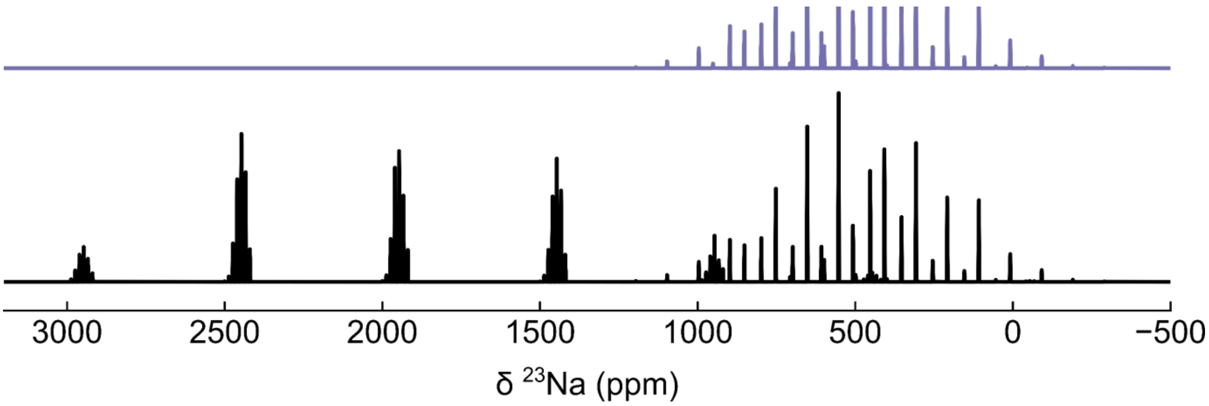


**Figure S29:** Calculated isotropic $^{23}Na$ NMR spectra for pristine $Na_{2/3}[Mg_{1/3}Mn_{2/3}]O_2$, where all $Na^+$ are assumed to remain in one site only during the NMR experiment and the distribution of $Mn^{4+}$ and $Mg^{2+}$ is random. Note that the average of the Hyb35 and Hyb20 shifts have been used to calculate the spectrum, with 1 ppm full width at half maxima. The simulations use 1 ppm linewidths, so that the isotropic resonance shifts may be clearly identified.

The honeycomb arrangement of $Mn^{4+}$ and $Mg^{2+}$ ions in the *TM*$O_2$ layer produces only one P(2d) environment and three P(2b) environments [Figure S30(b)], whose nearest neighbours and calculated shifts are presented in Table S6, alongside the estimated probabilities of each site. The probability for the P(2d) site was set to the probability of occupying this crystallographic site (i.e., 0.6); for the P(2b) sites, each environment was set with equal probability (0.133), so that the total probability of occupying P(2b) site was 0.4 (i.e., the probability of occupying this crystallographic site). These probabilities are only estimates, as it assumes that AA and AB honeycomb stacking of the *TM*$O_2$ layers is equally likely, which, in reality, may be unlikely.

Regardless of the estimated probabilities, these honeycomb-ordered shifts also do not match those observed in pristine NMMO. As a result, we assign the pristine NMMO spectrum to $Na^+$ ions exchanging on the timescale of the NMR experiment.

This results in a shift which is the dynamic average of the shifts of the sites between which $Na^+$ hops, $\delta_{\mathrm{avg.}}$:[38]

$$\delta_{\mathrm{avg.}} = \frac{\delta_{\mathrm{A}} + K\delta_{\mathrm{B}}}{1 + K},$$

(a)

| Site Type: | P(2d) | P(2b) | P(2b) | P(2b) |
|---|---|---|---|---|
| Environment: | 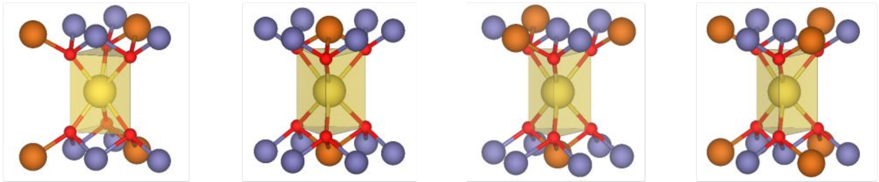 | | | |
| Hyb35 Shift: | 1785 ppm | 1145 ppm | 591 ppm | 37 ppm |
| Hyb20 Shift: | 2108 ppm | 1247 ppm | 713 ppm | 179 ppm |
| Probability: | 0.6 | 0.133 | 0.133 | 0.133 |

(b)

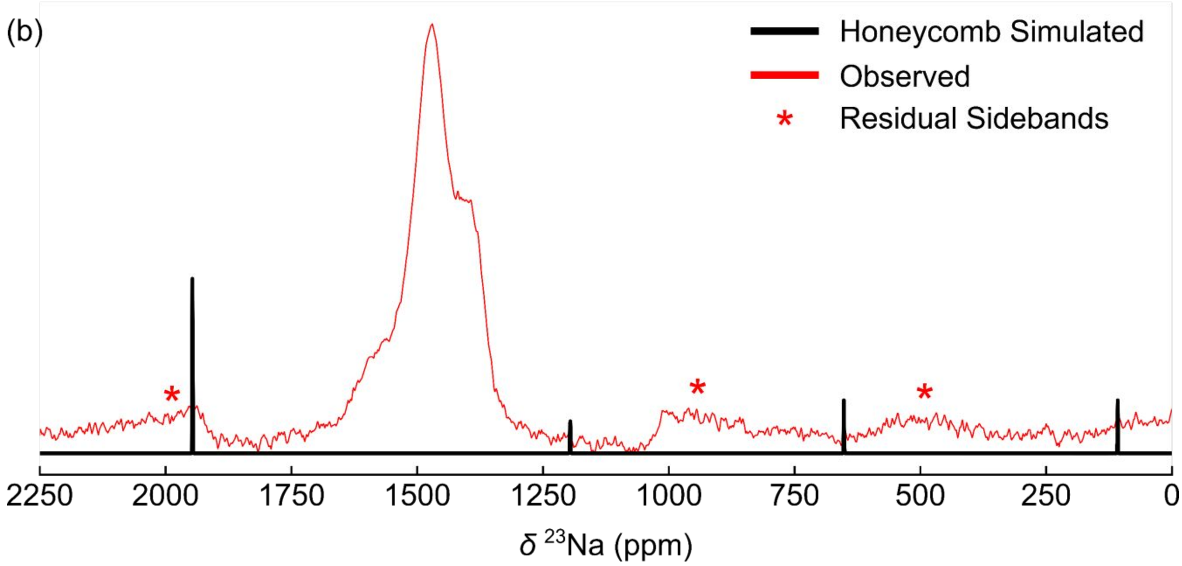


**Figure S30: (a)** shows the local Na P(2d) and P(2b) sites for honeycomb-ordered P2-$Na_{2/3}[Mg_{1/3}Mn_{2/3}]O_2$, alongside the calculated hyperfine shifts (calculated using hybrid 20 and hybrid 35 functionals) obtained from bond pathway analysis and the probabilities assigned to these sites in the simulated spectrum.
**(b)** shows the simulated spectrum for the sites shown in **(a)**, with 1 ppm linewidths for each site (chosen to clearly identify the isotropic shift).

where $\delta_A$ and $\delta_B$ are the shifts of the sites between which $Na^+$ hops and $K$ is an equilibrium constant which accounts for the relative rates of hopping between sites A and B:

$$K = \frac{k_{A\rightarrow B}}{k_{B\rightarrow A}},$$

where $k_{A\rightarrow B}$ is the rate constant for hopping from A to B and $k_{B\rightarrow A}$ the rate constant for hopping from B to A.

In the limit of infinitely fast exchange between all P(2d) sites and all P(2b) sites in the 'random' model, the average shift is 881 – 1058 ppm (where the ranges result from the values generated when using different hybrid functionals in the calculations); in the honeycomb-ordered model, this average is 890 – 1062 ppm.

Determination of the rate constants (and hence the equilibrium constants) is beyond the scope of this work, as it requires variable-temperature $^{23}Na$ NMR experimental data and detailed calculations of the $Na^+$ ion hopping energy barriers and attempt frequencies for several local environments and at several $Na^+$ compositions.

Qualitatively, however, changes in the shift can be interpreted as a change in $Na^+$ mobility: if the rates of hopping between A and B change (due to a change in the energy barriers of hopping brought about by a change in the local structure of NMMO), the equilibrium constant and hence the dynamically-averaged shift will necessarily change.

## 10. Additional Single-ended Transition State Searches for OP4- and O2-$Na_x[Mg_{0.28}Mn_{0.72}]O_2$

Note that all TS search calculations in this work were performed on charge balanced cells, in which all Mn sites were in the +4 oxidation state and all O sites were in the 2− oxidation state. Whilst the true oxidation states of these ions at the states of charge where $Mg^{2+}$ migration is seen is anticipated to be different, the examination of the electronic structures of these materials is beyond the scope of this work.

To assess the thermodynamic feasibility of $Mg^{2+}$ migration, we carried out single-ended transition state (TS) searches on the O-type layers of O2- and OP4-NMMO. Figure 5 in the

main text shows the energy profiles observed, alongside how the energy barriers to migration into and out of the tetrahedral sites in the O-type layers, $Mg^{2+}_{tet.}$, vary as a function of the lattice parameters of the cell. Figure S31 below shows the energy profiles observed for the different cell sizes of the O2 phase.

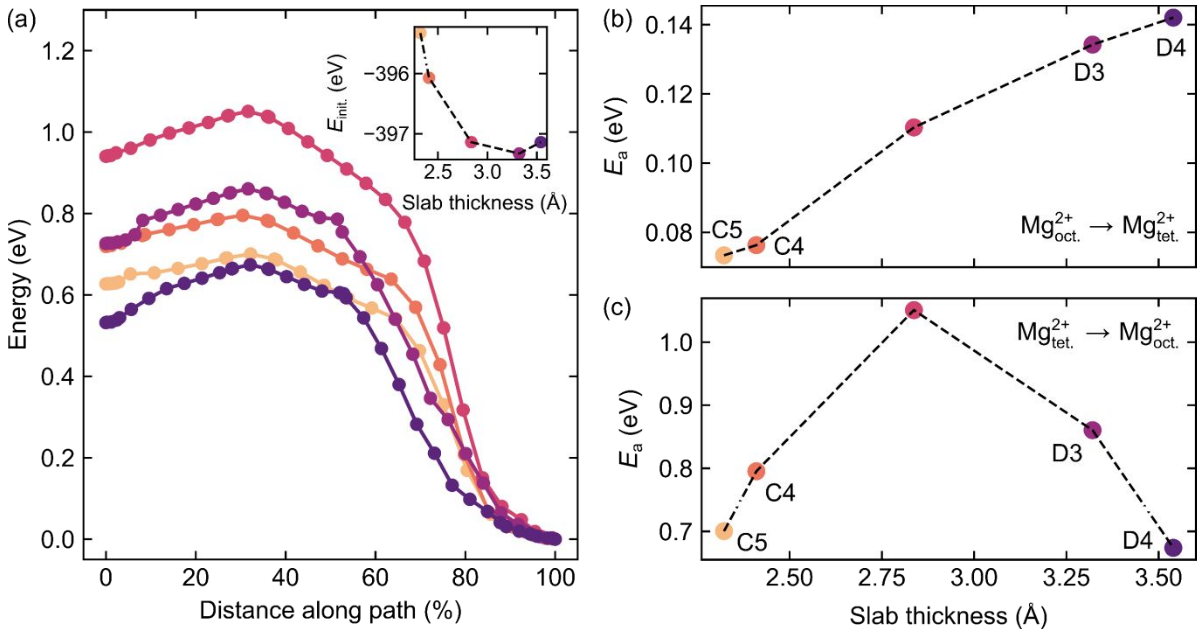


**Figure S31: (a)** shows the energy profiles for $Mg^{2+}$ migration in O2-NMMO cells (composition $Na_8Mg_4Mn_{14}O_{36}$) with different *a* and *c* lattice parameters (shown here as a change in the $Na^+$ layer slab thickness); inset shows the variation in the absolute energies of the final states. **(b)** and **(c)** show how the activation energy barriers for the migration of $Mg^{2+}$ in O2-NMMO from $Mg^{2+}_{oct.}$ to $Mg^{2+}_{tet.}$ and from $Mg^{2+}_{tet.}$ to $Mg^{2+}_{oct.}$, respectively, vary as the $Na^+$ layer slab thickness varies.

In addition, we carried out similar TS searches on OP4-NMMO [Figure S32]: as the $Na^+$ layer slab thickness increased, the barrier to migration in either direction (from $Mg^{2+}_{oct.}$ to $Mg^{2+}_{tet.}$ or from $Mg^{2+}_{tet.}$ to $Mg^{2+}_{oct.}$) decreased. The origin of the decrease observed likely stems from the absolute thickness of the $Na^+$ layer: in OP4 NMMO, the O-type layers are much thinner than in O2-NMMO, resulting in greater repulsion between $Mg^{2+}$ and the axial $O^{2-}$ ligand. Therefore, on increasing the slab thickness, the barrier to migration decreases.

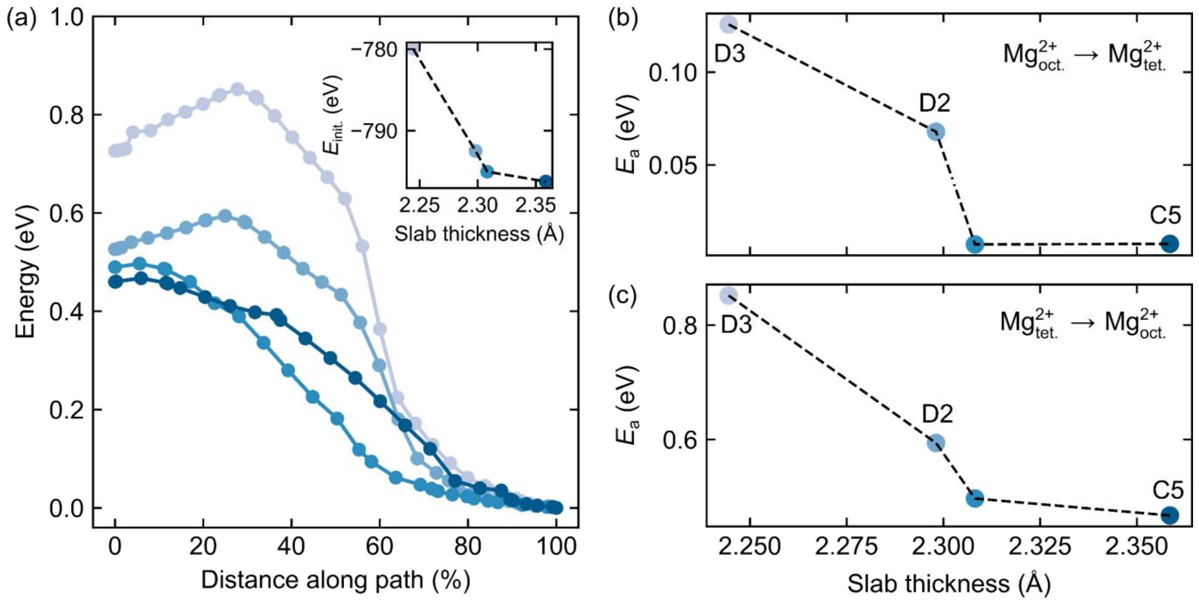


**Figure S32: (a)** shows the energy profiles for $Mg^{2+}$ migration in OP4-NMMO cells (composition $Na_{18}Mn_{27}Mg_9O_{72}$) with different *a* and *c* lattice parameters (shown here as a change in the $Na^+$ layer slab thickness); inset shows the variation in the absolute energies of the final states. **(b)** and **(c)** show how the activation energy barriers for the migration of $Mg^{2+}$ in OP4-NMMO from $Mg^{2+}_{oct.}$ to $Mg^{2+}_{tet.}$ and from $Mg^{2+}_{tet.}$ to $Mg^{2+}_{oct.}$, respectively, vary as the $Na^+$ layer slab thickness varies.

As well as the cell size, the presence of $Na^+$ in the O-type layer into which $Mg^{2+}$ migrates affects the migration barrier size. To investigate this, one $Na^+$ was placed in the O-type layer into which $Mg^{2+}$ migrates and a series of TS searches with different $Na^+$--$Mg^{2+}$ distances were carried out [Figure S33]. Note that the distance plotted in Figure S33 is the average $Na^+$--$Mg^{2+}$ distance of the two nearest-neighbour $Na^+$ centres to $Mg^{2+}$. As anticipated, as the $Na^+$--$Mg^{2+}$ distance decreased, the barrier increased significantly. At longer distances, however, the barriers increased slightly. The origin of this increase is not entirely clear, but likely arises from small changes in the $Na^+$ position as $Mg^{2+}$ migrates.

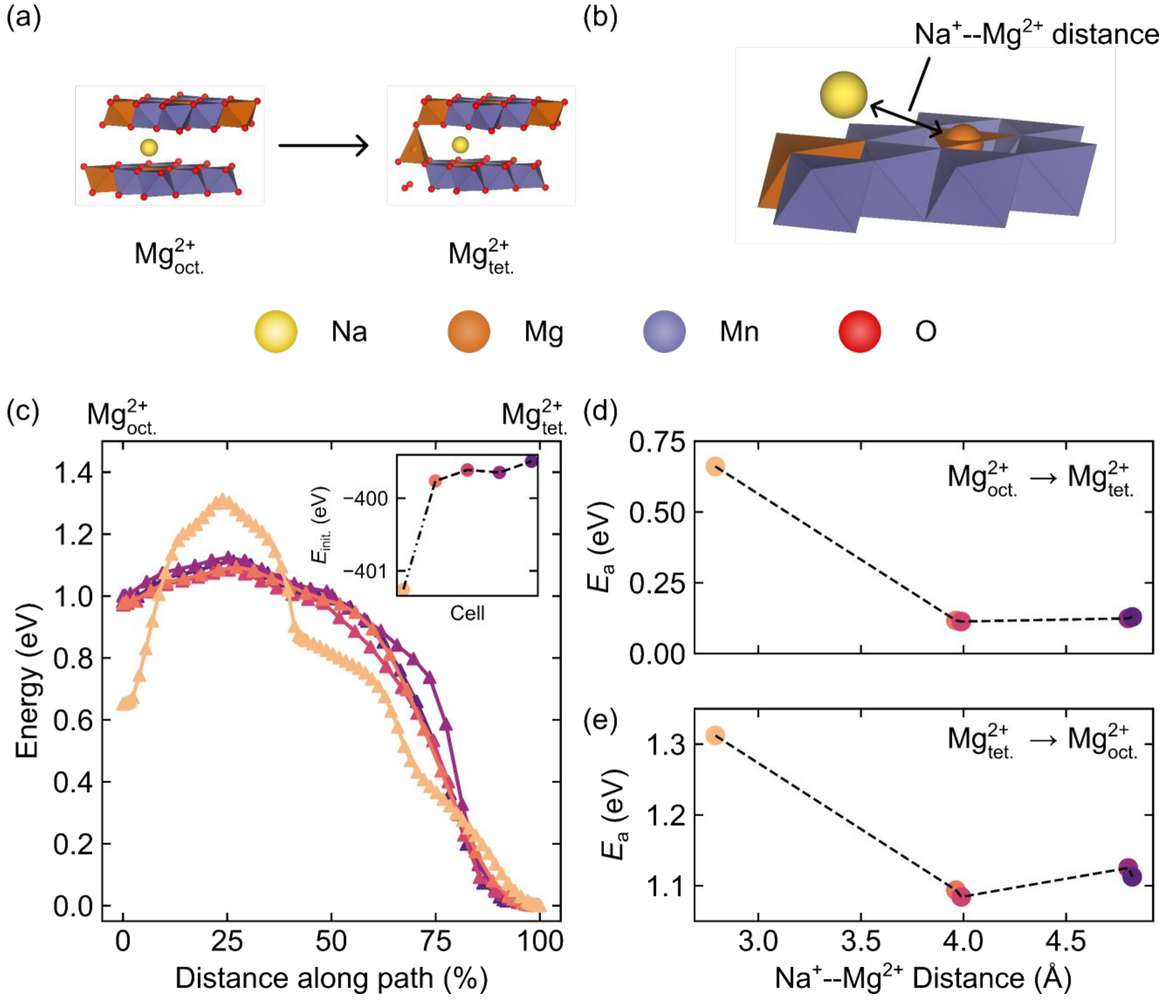


**Figure S33: (a)** shows a schematic of the $Mg^{2+}$ migration mechanism as $Mg^{2+}$ moves from the octahedral site in the $TMO_2$ layer to a tetrahedral site in the O-type $Na^+$ layer; inset shows the variation in the absolute energy of each of the final states. **(b)** highlights the TS for $Mg^{2+}$ migration, with the $Na^+$--$Mg^{2+}$ distance highlighted. **(c)** shows the energy profiles for $Mg^{2+}$ migration in O2-NMMO cells (composition $Na_{10}Mg_5Mn_{13}O_{36}$) with different *a* and *c* lattice parameters. **(d)** and **(e)** show how the activation energy barriers for the migration of $Mg^{2+}$ in O2-NMMO from $Mg^{2+}{}_{oct.}$ to $Mg^{2+}{}_{tet.}$ and from $Mg^{2+}{}_{tet.}$ to $Mg^{2+}{}_{oct.}$, respectively, vary as the average distance between $Na^+$ and the migrating $Mg^{2+}$ in the transition state varies.

We also investigated the effect of stacking faults on the migration energy profile. By ‘stacking fault’, we refer to the relative position of the $Mg^{2+}$ and $Mn^{4+}$ ions in the $TMO_2$ layers adjacent to the O-type layer into which $Mg^{2+}$ migrates (here, the O-type layer into which $Mg^{2+}$ migrates contained no $Na^+$). We have (arbitrarily) denoted the scenario where $Mg^{2+}$ and $Mn^{4+}$ occupy the same relative position in the layers (so-called AA ordering) as having no stacking faults [Figure S34(b)], whilst the scenario where $Mg^{2+}$ and $Mn^{4+}$ occupy different positions in the layers (known as AB ordering) are stacking faults [Figure S34(c)]. The energy profiles for the O2 and OP4 phases are highlighted in Figure S34(a). The general shape of the energy profiles for both phases remains approximately the same, whilst the activation energies for migration increase in the O2 phase and remain approximately constant in the OP4 phase.

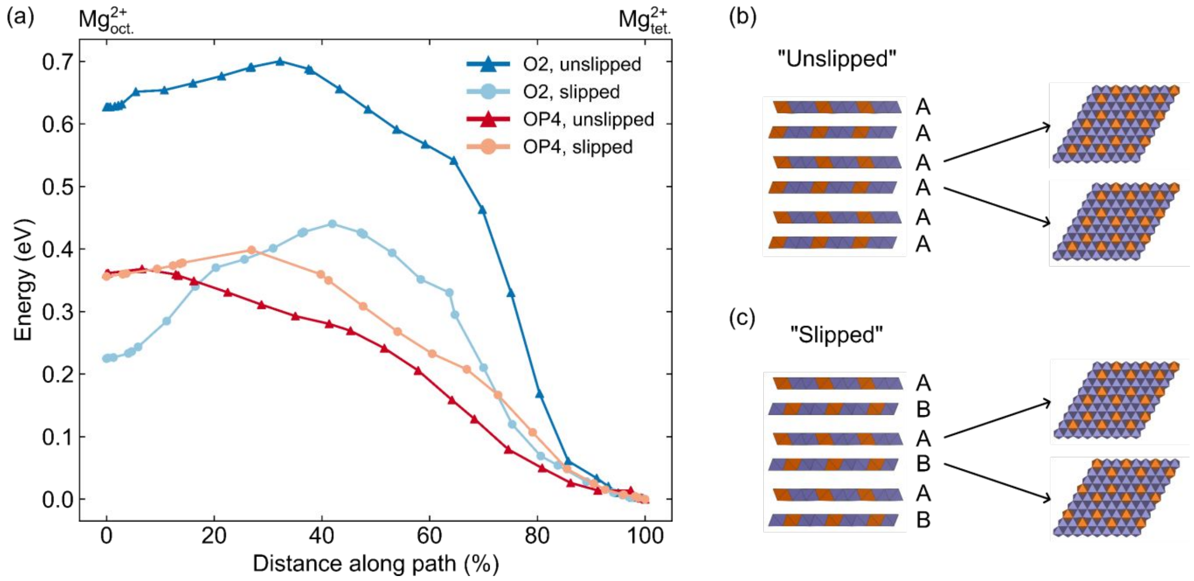


**Figure S34: (a)** Energy profiles for $Mg^{2+}$ migration for O2- and OP4-NMMO (compositions $Na_8Mg_4Mn_{14}O_{36}$ and $Na_{18}Mn_{27}Mg_9O_{72}$, respectively) with and without stacking faults, with schematics of the AA/AB stacking faults shown in **(b)** and **(c)** for O2-NMMO. The charge-balancing $Na^+$ ions in the structure have been omitted for clarity.

To identify whether $Mg^{2+}$ migration into the vacant octahedral sites of the O-type layers of the *Z*-phase was thermodynamically feasible, we investigated the energy profile for O2-NMMO with $Mg^{2+}$ migrating from a tetrahedral site in the $Na^+$ O-type layer, $Mg^{2+}_{tet.}$, to a neighbouring octahedral site, $Mg^{2+}_{Na,\ oct.}$, *via* TS in which $Mg^{2+}$ sits in the face shared between the tetrahedron and octahedron [Figure S35(a)]. The energy profiles are highlighted in Figure S35(b); these show that the $Mg^{2+}_{tet.}$ configuration is a lower energy state than the octahedral site, with a barrier to migration from $Mg^{2+}_{tet.}$ to $Mg^{2+}_{Na,\ oct.}$ of approximately 900 meV; the reverse process has a barrier of approximately 50 meV. This lies above the range of overpotentials applied and we do not anticipate that this barrier will be readily overcome—i.e., we anticipate $Mg^{2+}$ to preferentially sit in the tetrahedral sites of the O-type $Na^+$ layers. In other words, the tetrahedral sites of the vacant O-type $Na^+$ layers are thermodynamically more favourable and kinetically easier to access, compared to the vacant octahedral sites in the same layer.

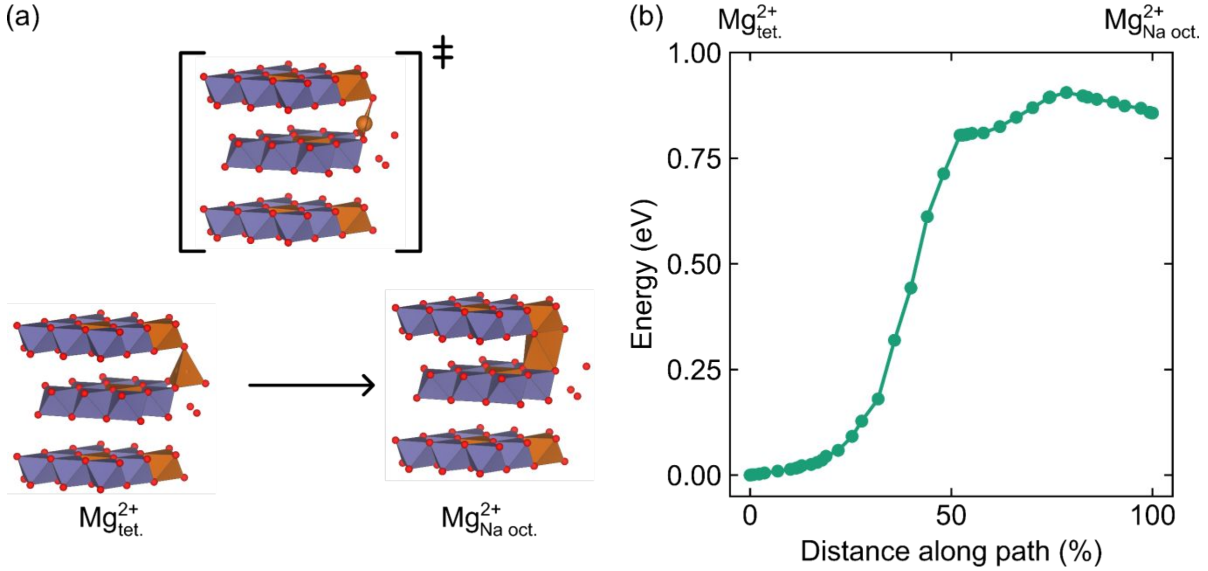


**Figure S35: (a)** shows a schematic of the $Mg^{2+}$ migration mechanism, where $Mg^{2+}$ moves from a tetrahedral site in the O-type $Na^+$ layer, $Mg^{2+}_{tet.}$, to a nearby octahedral site in the same O-type layer, $Mg^{2+}_{Na\ oct.}$.
**(b)** shows the energy profile for this process in an O2 cell with composition [illegible]

## 11. Magnetic Susceptibility Measurements

To accurately scale the Fermi contact shifts calculated for all $Na^+$ sites in NMMO, the effective magnetic moment, $\mu_{eff.}$, and the Weiss constant, $\theta$, are required. These were obtained by fitting the experimentally measured zero-field-cooled (ZFC) magnetic susceptibilities [Figure S36] to the Curie-Weiss Law. The fitted parameters are given in Table S7.

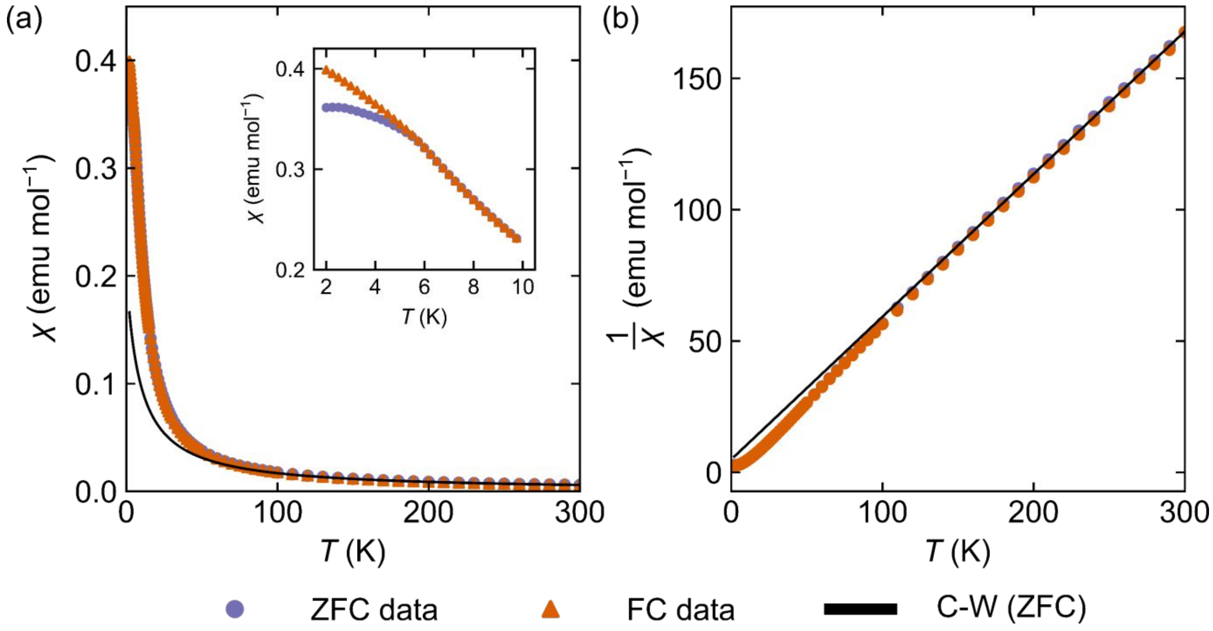


**Figure S36:** Zero-field cooled (ZFC) and field-cooled (FC) magnetic susceptibility data collected for pristine NMMO in a constant magnetic field strength of $H$ = 0.01 T. **(a)** shows the variation in magnetic susceptibility as a function of temperature, with inset showing an expansion of the low-temperature ($T$ < 10 K) region. **(b)** shows the inverse of the magnetic susceptibility, used to examine the Curie-Weiss law fit.

**Table S7:** Bulk magnetic susceptibility parameters, extracted from the raw ZFC magnetic susceptibility data ($T_{ZFC\text{-}FC}$) and high-temperature Curie-Weiss law fits ($\theta$, $\mu_{eff}$ and $g$). Experimental errors are given in parentheses.

| | |
|---|---|
| $T_{ZFC\text{-}FC}$ (K) | 6.5(25) |
| $\theta$ (K) | −9.1(5) |
| $\mu_{eff}$ ($\mu_B$) | 3.8(5) |
| $g$ | 1.9(2) |

## 12. Representative $Na^+$/Vacancy Ordering Schemes for P2-$Na_x[Mg_{0.28}Mn_{0.72}]O_2$

The *ex situ* $^{23}$Na NMR spectra of NMMO showed significant changes in line shape (i.e., peak breadth and intensity) during charge and discharge. In particular, a significant decrease in peak intensity was observed for the composition $x$ = 0.625 (point C1)—despite very little change in the $Na^+$ content compared to the pristine material—followed by a sharpening of the resonances for $x \leq 0.54$. Since the $^{23}$Na NMR spectra for NMMO arise from $Na^+$ exchanging between P(2d) and P(2b) sites over a timescale shorter than the NMR experiment, the loss of signal intensity with little change in $Na^+$ content suggests slower (less frequent) $Na^+$ hopping, perhaps due to $Na^+$/vacancy ordering. Figure S37 highlights some of the possible $Na^+$/vacancy ordering schemes for $x$ = 0.625 and $x$ = 0.54; whilst we only consider ordering over a (2×2×1) supercell, we anticipate that these are representative of the possible ordering schemes adopted.

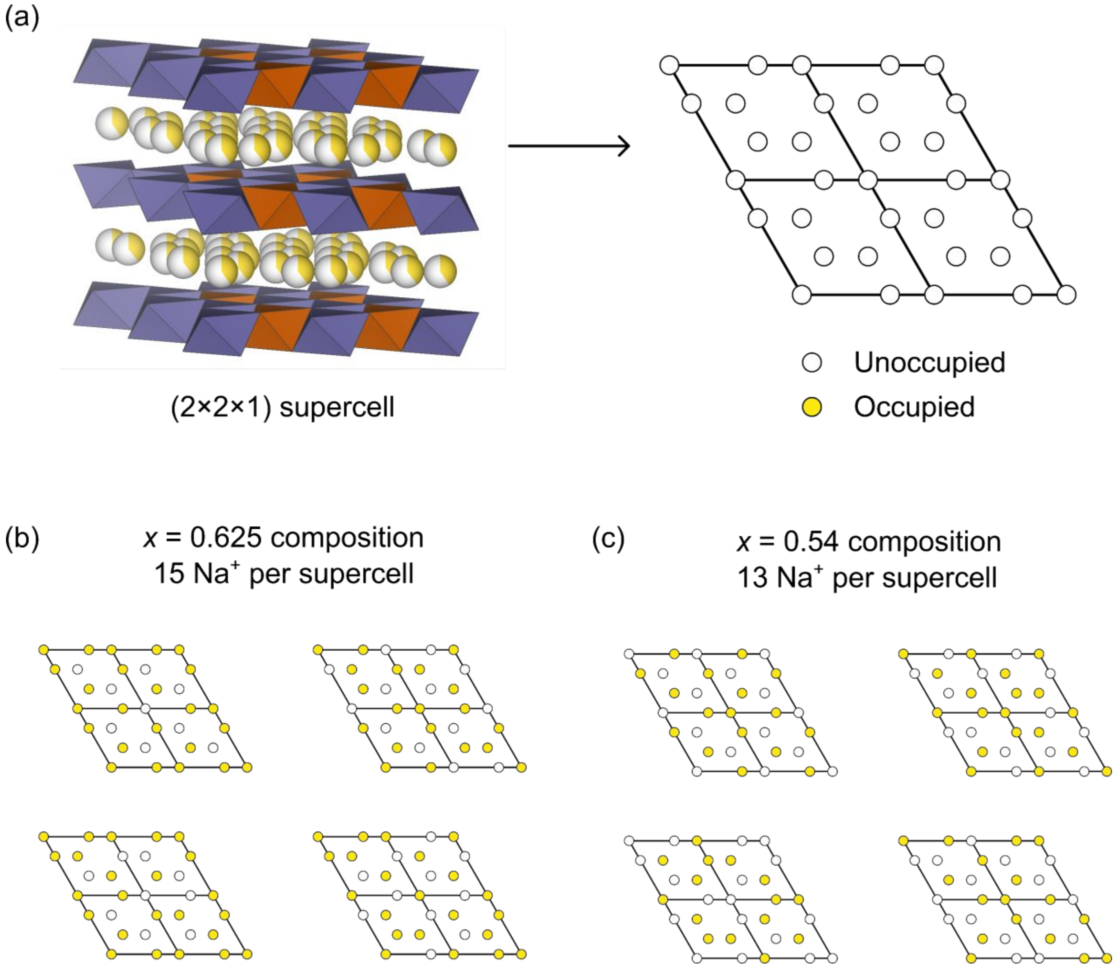


**Figure S37:** Schematic $Na^+$/vacancy orderings in a (2×2×1) supercell of P2-NMMO. **(a)** shows the simplified lattice used to describe the $Na^+$ and vacancy positions in a single P-type $Na^+$ layer. **(b)** and **(c)** show representative $Na^+$/vacancy arrangements for the compositions $x$ = 0.625 and $x$ = 0.54, respectively.

For the $x = 0.625$ composition, we anticipate the formation of an ordered array of $Na^+$ and vacancies, similar to the ordering seen in $Na_xMnO_2$.[39] If one $Na^+$ in the proposed ordering schema (for $x = 0.625$) hops to a neighbouring vacancy, the net $Na^+/Na^+$ repulsion increases, thereby increasing the energy barrier to hopping and slowing $Na^+$ hopping. In contrast, for $x = 0.54$, the increase in the number of neighbouring vacancies enables faster $Na^+$ motion, as $Na^+$ hopping does not lead to a large increase in $Na^+/Na^+$ repulsion.

## 13. Galvanostatic Intermittent Titration Technique (GITT) Results

To assist our understanding of the variation in $Na^+$ ion mobility in NMMO during the first charge-discharge cycle, we performed GITT experiments [Figures S38 and S39]. In a GITT experiment, a brief current pulse is applied to the cell, followed by a long rest period where no current passes through the cell. Measurement of the voltage of the cell throughout both the current pulse and resting periods gives valuable information about the kinetics of the charge/discharge mechanisms—i.e., the diffusivity and/or rate of phase transformations. The absolute diffusivity of $Na^+$ ions, $D$, however, cannot be assessed, due to difficulties in obtaining the values of the area, $S$ and molar volumes, $V_{\mathrm{m}}$, of the active material during charge and discharge. Instead, relative changes in the diffusivity are measured with the surrogate quantity $D\left(\frac{S}{V_{\mathrm{m}}}\right)^2$ (which will henceforth be referred to as the relative diffusivity), which assumes that both $S$ and $V_{\mathrm{m}}$ are fixed throughout charge and discharge. This quantity is only meaningful for single-phase regions of the voltage profile; two-phase regions are more challenging to treat, as the value of $D\left(\frac{S}{V_{\mathrm{m}}}\right)^2$ contains contributions from both $Na^+$ diffusivity and the rate of phase transformation. Analytical methods to extract the diffusivity in each phase have been developed[40] but are beyond the scope of this work.

On charge, the variation of the OCV (which reflects the free energy of the phases present) with $Na^+$ content does not match the voltage profile seen, whilst the variation in overpotentials (a measure of the additional driving force needed to continue the electrochemical reaction) with $Na^+$ content does [Figures S38(a)]. This suggests that, on charge, $Na^+$ ion extraction is kinetically limited, either due to slow $Na^+$ ion motion or slow charge transfer processes. Based on the NMR data, where relatively sharp resonances due to rapidly-exchanging $Na^+$ species were observed, we anticipate that slow $Na^+$ ion motion makes little contribution to the overpotentials seen, up until the end of the first charge. Therefore, we anticipate that the energy barrier to $Na^+$ extraction stems from the barrier required to remove an electron from the material, perhaps because this process is coupled with $Mg^{2+}$ migration and has a high-energy transition state.

The relative diffusivity of $Na^+$ ions on charge goes through a series of peaks and troughs [Figure S38(b)]: the peaks likely correspond to a 'disordered' arrangement of $Na^+$ ions and vacancies, while the troughs likely correspond to $Na^+$/vacancy ordering schemes. At relatively high $Na^+$ contents, there are few vacancies, so the effect of $Na^+$ ions hopping is to increase the total energy by increasing the number of repulsive $Na^+$-$Na^+$ interactions. At lower $Na^+$ contents (i.e., higher vacancy concentrations), the effect of $Na^+$ ions hopping on the overall energy of the system is smaller, as $Na^+$ ions are further apart and experience fewer repulsive interactions. Consequently, the resonances seen in $^{23}$Na NMR remain sharp for points C2, C3 and C4, but the resonances at point C1 are broadened due to the additional $T_2$ relaxation effect associated with $Na^+$ motion in the intermediate or slow regimes.

On discharge, the OCV and overpotentials match more closely for $Na^+$ contents $0.25 < x < 0.75$; outside of this range, the overpotential significantly diverges from the OCV [Figure S39(a)]. For the low $Na^+$ content region ($x < 0.25$), the overpotentials likely stem from $Mg^{2+}$ migration back into the $TMO_2$ layers, whilst at the end of discharge, these overpotentials likely stem from slow $Na^+$ motion (due to the large number of $Na^+$ ions and significant $Na^+$-$Na^+$ repulsion).

The relative diffusivity on discharge similarly goes through a series of peaks and troughs, again presumably due to $Na^+$/vacancy ordering [Figure S39(b)]. The relative diffusivity on

discharge is also greater than on charge. We ascribe this to the fact that, on charge, the relative diffusivity parameter accounts for the rate of the two-phase reaction between P2 and the *Z*-phase, whilst on discharge, only a single-phase reaction occurs. The relative diffusivity on charge is therefore a convolution of the $Na^+$ diffusivity and the rate of the two-phase reaction, leading to an apparently lower relative diffusivity.

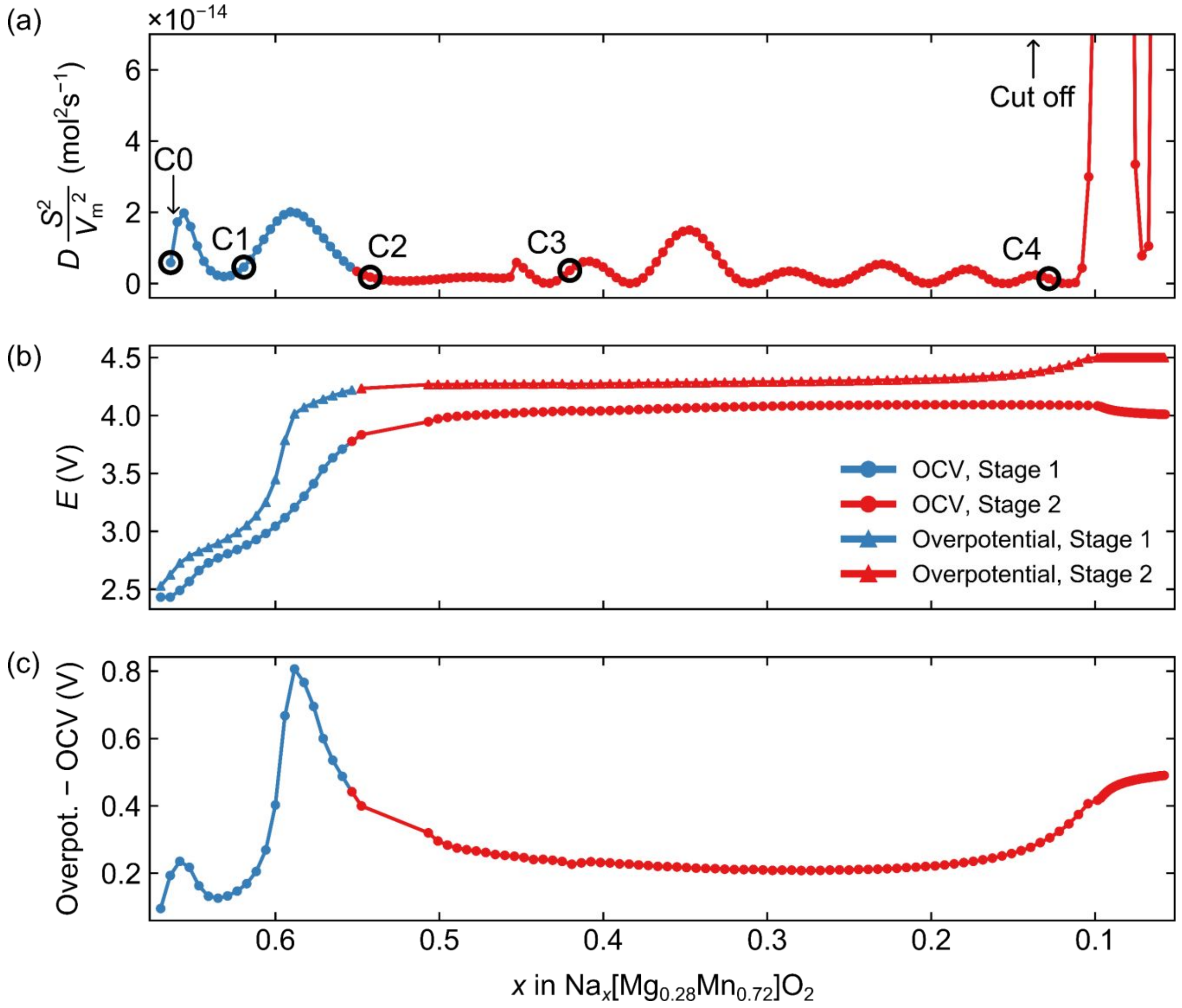


**Figure S38:** GITT data for the first charge half-cycle of NMMO. As in the main text, data in blue represents Stage 1, whilst data in red represents Stage 2. **(a)** shows the variation in the relative diffusivity, $D\left(\frac{S}{V_{\mathrm{m}}}\right)^2$, as a function of the $Na^+$ content of NMMO, with *ex situ* points C0, C1, C2, C3 and C4 highlighted. Note that point C5 is cut off, due to the sudden jump in relative diffusivity; also note that the relative diffusivities for the first eight points overestimate the $Na^+$ ion mobility, as the cell was not fully relaxed when the next current pulse was applied. **(b)** shows the variation in the open circuit voltage (OCV) and overpotential of NMMO as a function of $Na^+$ content. **(c)** shows the difference in the overpotential and OCV as a function of $Na^+$ content.

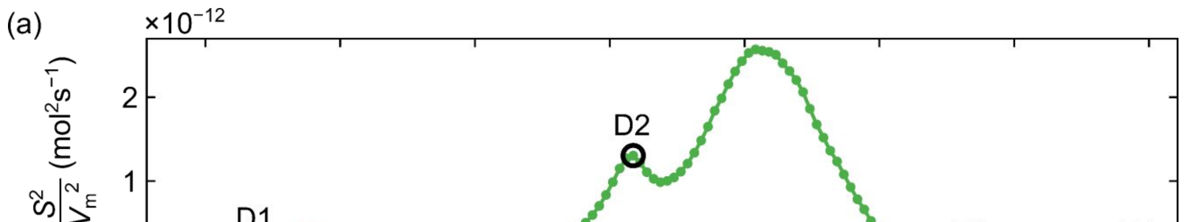

## 14. References

(1) Coelho, A. TOPAS-Academic: General Profile and Structure Analysis Software for Powder Diffraction Data. Brisbane, Australia 2007.

(2) Coelho, A. A. *TOPAS* and *TOPAS-Academic* : An Optimization Program Integrating Computer Algebra and Crystallographic Objects Written in C++. *J. Appl. Crystallogr.* **2018**, *51* (1), 210–218.

(3) Verma, A.; Smith, K.; Santhanagopalan, S.; Abraham, D.; Pierre Yao, K.; Mukherjee, P. P. Galvanostatic Intermittent Titration and Performance Based Analysis of LiNi 0.5 Co 0.2 Mn 0.3 O 2 Cathode. *J. Electrochem. Soc.* **2017**, *164* (13), 3380–3392.

(4) Wasylishen,R.; Ashbrook, S.; Wimperis, S. Vega, A. J., Quadrupolar Nuclei in Solids. In *NMR of Quadrupolar Nuclei in Solid Materials*; Wiley, 2012; pp 17–44.

(5) Clément, R. J.; Pell, A. J.; Middlemiss, D. S.; Strobridge, F. C.; Miller, J. K.; Whittingham, M. S.; Emsley, L.; Grey, C. P.; Pintacuda, G. Spin-Transfer Pathways in Paramagnetic Lithium Transition-Metal Phosphates from Combined Broadband Isotropic Solid-State MAS NMR Spectroscopy and DFT Calculations. *J. Am. Chem. Soc.* **2012**, *134* (41), 17178–17185.

(6) Middlemiss, D. S.; Ilott, A. J.; Clément, R. J.; Strobridge, F. C.; Grey, C. P. Density Functional Theory-Based Bond Pathway Decompositions of Hyperfine Shifts: Equipping Solid-State NMR to Characterize Atomic Environments in Paramagnetic Materials. *Chem. Mater.* **2013**, *25* (9), 1723–1734.

(7) Kim, J.; Middlemiss, D. S.; Chernova, N. A.; Zhu, B. Y. X.; Masquelier, C.; Grey, C. P. Linking Local Environments and Hyperfine Shifts: A Combined Experimental and Theoretical 31 P and 7 Li Solid-State NMR Study of Paramagnetic Fe(III) Phosphates. *J. Am. Chem. Soc.* **2010**, *132* (47), 16825–16840.

(8) Kim, J.; Ilott, A. J.; Middlemiss, D. S.; Chernova, N. A.; Pinney, N.; Morgan, D.; Grey, C. P. 2H and 27Al Solid-State NMR Study of the Local Environments in Al-Doped 2-Line Ferrihydrite, Goethite, and Lepidocrocite. *Chem. Mater.* **2015**, *27* (11), 3966–3978.

(9) Kresse, G.; Hafner, J. Ab Initio Molecular Dynamics for Liquid Metals. *Phys. Rev. B* **1993**, *47* (1), 558–561.

(10) Kresse, G.; Furthmüller, J. Efficiency of Ab-Initio Total Energy Calculations for Metals and Semiconductors Using a Plane-Wave Basis Set. *Comput. Mater. Sci.* **1996**, *6* (1), 15–50.

(11) Kresse, G.; Hafner, J. Ab Initio Molecular-Dynamics Simulation of the Liquid-Metalamorphous-Semiconductor Transition in Germanium. *Phys. Rev. B* **1994**, *49* (20), 14251–14269.

(12) Blöchl, P. E. Projector Augmented-Wave Method. *Phys. Rev. B* **1994**, *50* (24), 17953–17979.

(13) Kresse, G.; Joubert, D. From Ultrasoft Pseudopotentials to the Projector Augmented-Wave Method. *Phys. Rev. B* **1999**, *59* (3), 1758–1775.

(14) Anisimov, V. I.; Zaanen, J.; Andersen, O. K. Band Theory and Mott Insulators: Hubbard U Instead of Stoner I. *Phys. Rev. B* **1991**, *44* (3), 943–954.

(15) Anisimov, V. I.; Solovyev, I. V.; Korotin, M. A.; Czyyk, M. T.; Sawatzky, G. A. Density-Functional Theory and NiO Photoemission Spectra. *Phys. Rev. B* **1993**, *48* (23), 16929–16934.

(16) Liechtenstein, A. I.; Anisimov, V. I.; Zaanen, J. Density-Functional Theory and Strong Interactions: Orbital Ordering in Mott-Hubbard Insulators. *Phys. Rev. B* **1995**, *52* (8), R5467.

(17) Zhou, F.; Cococcioni, M.; Marianetti, C. A.; Morgan, D.; Ceder, G. First-Principles Prediction of Redox Potentials in Transition-Metal Compounds with LDA + U. *Phys. Rev. B* **2004**, *70* (23), 235121-1-235121–235128.

(18) Li, X.; Ma, X.; Su, D.; Liu, L.; Chisnell, R.; Ong, S. P.; Chen, H.; Toumar, A.; Idrobo, J.-C.; Lei, Y.; Bai, J.; Wang, F.; Lynn, J. W.; Lee, Y. S.; Ceder, G. Direct Visualization of the Jahn–Teller

Effect Coupled to Na Ordering in Na5/8MnO2. *Nat. Mater.* **2014**, *13* (6), 586–592.

(19) Monkhorst, H. J.; Pack, J. D. Special Points for Brillouin-Zone Integrations. *Phys. Rev. B* **1976**, *13* (12), 5188–5192.

(20) Dovesi, R.; Erba, A.; Orlando, R.; Zicovich-Wilson, C. M.; Civalleri, B.; Maschio, L.; Rérat, M.; Casassa, S.; Baima, J.; Salustro, S.; Kirtman, B. Quantum-Mechanical Condensed Matter Simulations with CRYSTAL. *Wiley Interdiscip. Rev. Comput. Mol. Sci.* **2018**, *8* (4), e1360.

(21) Becke, A. D. Density-functional Thermochemistry. III. The Role of Exact Exchange. *J. Chem. Phys.* **1993**, *98* (7), 5648–5652.

(22) Lee, C.; Yang, W.; Parr, R. G. Development of the Colle-Salvetti Correlation-Energy Formula into a Functional of the Electron Density. *Phys. Rev. B* **1988**, *37* (2), 785–789.

(23) Catti, M.; Sandrone, G.; Dovesi, R. Periodic Unrestricted Hartree-Fock Study of Corundumlike Ti2O3 and V2O3. *Phys. Rev. B* **1997**, *55* (24), 16122–16131.

(24) Catti, M.; Valerio, G.; Dovesi, R.; Causà, M. Quantum-Mechanical Calculation of the Solid-State Equilibrium MgO+α-Al2O3 ⇄ MgAl2O4 (Spinel) versus Pressure. *Phys. Rev. B* **1994**, *49* (20), 14179–14187.

(25) Catti, M.; Sandrone, G.; Valerio, G.; Dovesi, R. Electronic, Magnetic and Crystal Structure of Cr2O3 by Theoretical Methods. *J. Phys. Chem. Solids* **1996**, *57* (11), 1735–1741.

(26) Schäfer, A.; Horn, H.; Ahlrichs, R. Fully Optimized Contracted Gaussian Basis Sets for Atoms Li to Kr. *J. Chem. Phys.* **1992**, *97* (4), 2571–2577.

(27) Kutzelnigg, W.; Fleischer, U.; Schindler, M. The IGLO-Method: Ab-Initio Calculation and Interpretation of NMR Chemical Shifts and Magnetic Susceptibilities; Springer, Berlin, Heidelberg, 1990; pp 165–262.

(28) Lee, J.; Seymour, I. D.; Pell, A. J.; Dutton, S. E.; Grey, C. P. A Systematic Study of 25 Mg NMR in Paramagnetic Transition Metal Oxides: Applications to Mg-Ion Battery Materials. *Phys. Chem. Chem. Phys.* **2017**, *19* (1), 613–625.

(29) Bain, G. A.; Berry, J. F. Diamagnetic Corrections and Pascal's Constants. *J. Chem. Educ.* **2008**, *85* (4), 532–536.

(30) Rietveld, H. M. Line Profiles of Neutron Powder-Diffraction Peaks for Structure Refinement. *Acta Crystallogr.* **1967**, *22* (1), 151–152.

(31) Rietveld, H. M. A Profile Refinement Method for Nuclear and Magnetic Structures. *J. Appl. Crystallogr.* **1969**, *2* (2), 65–71.

(32) Hope, M. A. Solid-State NMR of Metallic and Paramagnetic Systems, University of Cambridge, 2019.

(33) Bielecki, A.; Burum, D. P. Temperature Dependence Of207Pb MAS Spectra of Solid Lead Nitrate. An Accurate, Sensitive Thermometer for Variable-Temperature MAS. *J. Magn. Reson. Ser. A* **1995**, *116* (2), 215–220.

(34) Beckmann, P. A.; Dybowski, C. A Thermometer for Nonspinning Solid-State NMR Spectroscopy. *Journal of Magnetic Resonance*. Academic Press Inc. October 1, 2000, pp 379–380.

(35) Goodenough, J. B. An Interpretation of the Magnetic Properties of the Perovskite-Type Mixed Crystals La(1-x)Sr(x)CoO(3-Y). *J. Phys. Chw. Solids Pergamon Press* **1958**, *6*, 287–297.

(36) Kanamori, J. Theory of the Magnetic Properties of Ferrous and Cobaltous Oxides, I. *Prog. Theor. Phys.* **1957**, *17* (2), 177–196.

(37) Kanamori, J. Theory of the Magnetic Properties of Ferrous and Cobaltous Oxides, II. *Prog. Theor. Phys.* **1957**, *17* (2), 197–222.

(38) Levitt, M. H. *Spin Dynamics*, 2nd ed.; John Wiley & Sons Ltd: Southampton, 2007.

(39) Toumar, A. J.; Ong, S. P.; Richards, W. D.; Dacek, S.; Ceder, G. Vacancy Ordering in O3 - Type Layered Metal Oxide Sodium-Ion Battery Cathodes. *Phys. Rev. Appl.* **2015**, *4* (6), 064002.

(40) Zhu, Y.; Wang, C. Galvanostatic Intermittent Titration Technique for Phase-Transformation Electrodes. *J. Phys. Chem. C* **2010**, *114* (6), 2830–2841.
